# Caledonian reactivation and reworking of Timanian thrust systems and implications for latest Mesoproterozoic to mid-Paleozoic tectonics and magmatism in northern Baltica

**DOI:** 10.12688/openreseurope.17033.2

**Published:** 2024-10-15

**Authors:** Jean-Baptiste P. Koehl, Eirik Stokmo

**Affiliations:** 1 Earth and Planetary Sciences, McGill University, Montreal, Québec, H3A 0E8, Canada; 2 Geosciences, Universitetet i Oslo, Oslo, Oslo, 0371, Norway; 3 Brønnøy Kalk, Velfjord, Nordland, 8960, Norway

**Keywords:** Baltica, Neoproterozoic, Paleozoic, Timanides, Timanian Orogeny, Caledonides, Caledonian Orogeny, thrust, shear zone, seismic reflection, magnetic, bathymetric, Porsanger Orogeny, Kalak Nappe Complex, Iapetus Ocean, Grenvillian Orogeny, Seiland Igneous Province, Trollfjorden–Komagelva Fault Zone

## Abstract

**Background:**

The Trollfjorden–Komagelva Fault Zone is the southernmost thrust fault of the Timanian Orogen and extends for thousands of kilometers from northwestern Russia to northern Norway. Though there is little about its location onshore northeastern Norway, where it is mapped as a major fault system dominantly comprised of NNE-dipping thrust faults, its continuation to the west below Caledonian nappes and offshore post-Caledonian sedimentary basins remains a matter of debate.

**Methods:**

The present study provides a more definitive answer about the continuation of Trollfjorden–Komagelva Fault Zone west of the Varanger Peninsula by using seismic reflection, bathymetric, topographic, and magnetic data onshore Finnmark and offshore on the Finnmark Platform.

**Results:**

The present study demonstrates that the Sørøya–Ingøya shear zone represents a portion of the Trollfjorden–Komagelva Fault Zone that was folded into a NE–SW orientation and reactivated as a top-southeast thrust during the Caledonian Orogeny, while other portions of the Trollfjorden–Komagelva Fault Zone (e.g., on the Varanger Peninsula) were reactivated as strike-slip faults. The study also documents the presence of another major, NNE-dipping Timanian shear zone with a similar geometry to the Trollfjorden–Komagelva Fault Zone north of the Varanger Peninsula.

**Conclusions:**

The Trollfjorden–Komagelva Fault Zone may continue offshore as a NE–SW-striking folded structure. This has the following implications: (1) the Seiland Igneous Province likely formed in a backarc setting, (2) metasedimentary rocks of the Kalak Nappe Complex deposited along the Baltican margin of the Iapetus Ocean, possibly in a late–post-Grenvillian collapse basin, (3) the Iapetus Ocean was much narrower than the several thousands of kilometers width commonly proposed, and (4) early Neoproterozoic magmatism in northern Norway is possibly related to the initial breakup of Rodinia.

## Introduction

The Timanian Orogeny is an episode of NNE–SSW-oriented contraction (all the structural directions are defined relative to present-day coordinates) that occurred in the latest Neoproterozoic (ca. 650–550 Ma) during which oceanic crust was possibly subducted under Baltica ( Pease *et al.*, 2004). Though footprints of this tectonic episode were found all over the Arctic (e.g., Estrada *et al.*, 2018a; Estrada *et al.*, 2018b; Rekant *et al.*, 2019), actual Timanian structures only crop out in northwestern Russia ( Olovyanishnikov *et al.*, 2000), northeasternmost Norway ( Siedlecka & Siedlecki, 1967; Siedlecka, 1975), and southwestern Spitsbergen ( Faehnrich *et al.*, 2020; Mazur *et al.*, 2009). Related structures also occur offshore in the Barents Sea and are buried deeply in Svalbard ( Eldholm & Ewing, 1971, their Figure 4 profile C–D; Koehl, 2020; Koehl *et al.*, 2022a; Koehl *et al.*, 2023; Korago *et al.*, 2004; Lopatin *et al.*, 2001).

**Figure 1.**
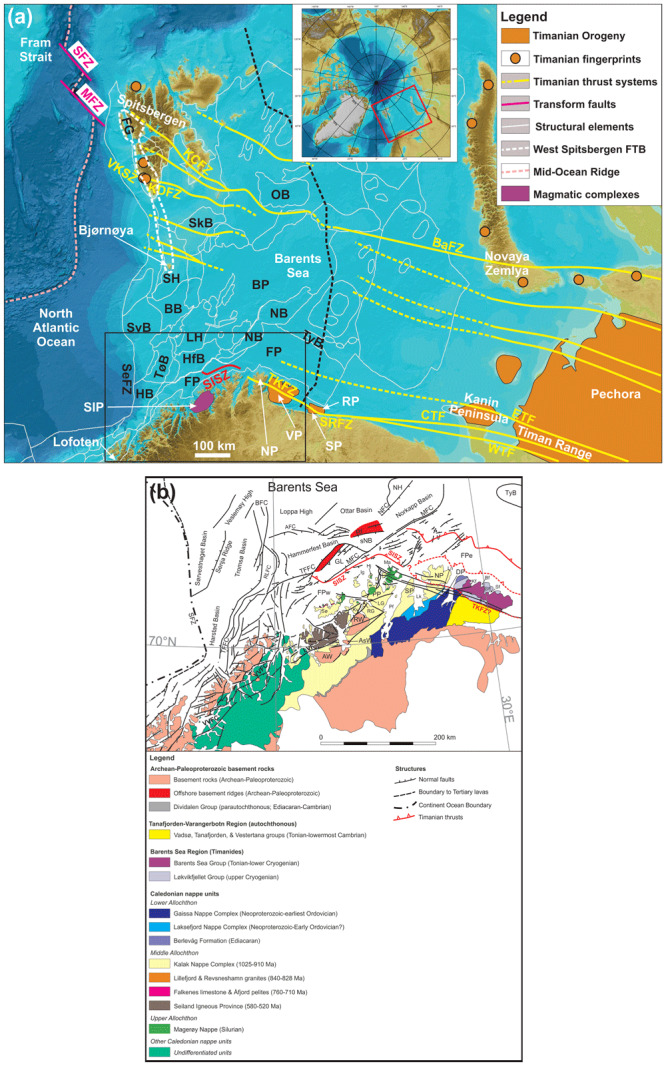
( **a**) Elevation map of the Barents Sea, northern Norway and northwestern Russia showing major structural elements from the Norwegian Offshore Directorate (thin white lines) and major fault trends in the Barents Sea (Timanian; yellow lines). The location of ( **a**) is shown as a red rectangle in the upper inset map and the location of ( **b**) as a black rectangle. The basemap and the upper inset are from Jakobsson *et al.* (2012). A Polar Stereographic projection was used (datum: WGS84). Abbreviations: AFC: Asterias Fault Complex; AKTW: Alta–Kvænangen tectonic window; BaFZ: Baidaratsky Fault Zone; BB: Bjørnøya Basin; BFC: Bjørnøyrenna Fault Complex; BP: Bjarmeland Platform; CTF: Central Timan Fault; ETF: East Timan Fault; FG: Forlandsundet Graben; FP: Finnmark Platform; FSB: Fingerdjupet Sub-Basin; FTB: Fold-and-Thrust Belt; HB: Harstad Basin; HfB: Hammerfest Basin; HFC: Hoop Fault Complex; JFC: Jason Fault Complex; KCFZ: Kongsfjorden–Cowanodden fault zone; KDFZ: Kinnhøgda–Daudbjørnpynten fault zone; LH: Loppa High; MB: Maud Basin; MFC: Måsøy Fault Complex; MFZ: Molloy Fracture Zone; MH: Mercurius High; NB: Nordkapp Basin; ND: Norsel Dome; NH: Norsel High; OB: NP: Nordkinn Peninsula; Olga Basin; PSP: Polhem Sub-Platform; RFC: Ringvassøya Fault Complex; RP: Rybachi Peninsula; SaD: Samson Dome; SeFZ: Senja Fracture Zone; SD: Sredni Peninsula; SFZ: Spitsbergen Fracture Zone; SH: Stappen High; SIP: Seiland Igneous Province; SISZ: Sørøya–Ingøya shear zone; SkB: Sørkapp Basin; SR: Senja Ridge; SRFZ: Sredni–Rybachi Fault Zone; SvB: Sørvestnaget Basin; SvD: Svalis Dome; TFFC: Troms–Finnmark Fault Complex; TKFZ: Trollfjorden–Komagelva Fault Zone; TyB: Tiddlybanken Basin; TøB: Tromsø Basin; VH: Veslemøy High; VKSZ: Vimsodden–Kosibapasset Shear Zone; VP: Varanger Peninsula; WTF: West Timan Fault. ( **b**) Geological map of northern Norway and the southern Barents Sea showing the main onshore and offshore structures and tectonostratigraphic units. The map is after Koehl *et al.* (2019) with updates after Siedlecka and Siedlecki (1967), Siedlecki (1980), Townsend *et al.* (1986), Rice (1994), Kirkland *et al.* ( 2005; 2006a; 2007a; 2007b; 2008a; 2008b), Indrevær *et al.* (2013), Corfu *et al.* (2014), Koehl *et al.* (2018a), Faber (2018, manuscript 3), and Roberts and Siedlecka (2022). Abbreviations: AFC – Asterias Fault Complex; AsW: Altenes tectonic window; AW: Alta–Kvænangen tectonic window; Bf: Båtsfjorden; BFC: Bjørnøyrenna Fault Complex; Bj: Bjørnøya; Bn: Båsnæringsfjellet; Bv: Berlevåg; DP: Digermulen Peninsula; GL: Gjesvær Low; Hj: Hjelmsøya; Ig: Ingøya;Kf: Kongsfjorden; Kv: Kvaløya; LG: Lillefjord Granite; Lk: Laksefjorden; Ln: Langfjorden; LVF: Langfjorden–Vargsundet fault; Ma: Magerøya; MFC: Måsøy Fault Complex; NFC: Nysleppen Fault Complex; NP: Nordkinn Peninsula; Pf: Porsangerfjorden; PP: Porsanger Peninsula; RA: Ragnarokk Anticline; Rf: Rolvsøya fault; Rk: Reinøykalven; RG: Revsneshamn Granite; RLFC: Ringvassøya–Loppa Fault Complex; Rv: Rolvsøya; RW: Repparfjord–Komagfjord tectonic window; Sf: Syltefjorden; Sff: Syltefjordfjellet; SFZ: Senja Fracture Zone; SISZ: Sørøya–Ingøya shear zone; Sk: Stikonjargga Peninsula; sNB – southwesternmost Nordkapp basin; SP: Sværholt Peninsula; Sø: Sørøya; TFFC: Troms–Finnmark Fault Complex; TKFZ: Trollfjorden–Komagelva Fault Zone; Tn: Tanafjorden; TyB: Tiddlybanken Basin; VP: Varanger Peninsula; VVFC: Vestfjorden–Vanna Fault Complex.

In northeasternmost Norway, the Timanian thrust front, the Trollfjorden–Komagelva Fault Zone ( Figure 1a), crops out on the Varanger Peninsula. In the east, this major fault boundary continues as the Sredni-Rybachi Fault Zone between the Sredni and Rybachi peninsulas, and as the West Timan Fault or Central Timan Fault farther east in the Timan Range and Kanin Peninsula ( Olovyanishnikov *et al.*, 2000; Figure 1a). In the west, the Trollfjorden–Komagelva Fault Zone continues as a series of WNW–ESE-striking brittle faults that merge with a NW–SE-striking segment of the Troms–Finnmark Fault Complex ( Gabrielsen, 1984; Gabrielsen & Færseth, 1989; Lea, 2016; Roberts *et al.*, 2011). However, recent studies suggest that tracking the western continuation of the Trollfjorden–Komagelva Fault Zone is not straightforward. New models involve a possible truncation of the Trollfjorden–Komagelva Fault Zone by a kilometer-thick, top-southeast Caledonian shear zone ( Koehl *et al.*, 2018a) or a splaying and dying out geometry ( Koehl *et al.*, 2019). The present study presents a new, more realistic interpretation of the Trollfjorden–Komagelva Fault Zone off northwestern Norway.

Previous work on the Varanger Peninsula ( Roberts, 1996; Siedlecka & Siedlecki, 1971; Figure 1a–b) and ongoing work in Svalbard and the northern Barents Sea ( Klitzke *et al.*, 2019; Koehl, 2020; Koehl *et al.*, 2022a; Koehl *et al.*, 2023) show that Timanian folds, thrusts and shear zones were refolded by NNE–SSW-trending Caledonian folds. The aim of this contribution is to explore the Finnmark Platform (i.e., the nearshore portion of the southwestern Barents Sea; Figure 1a–b) for traces of similarly folded Timanian structures and track their potential continuation onshore and in nearshore fjords.

The results of the present study challenge previous models proposed for the western, offshore continuation of the Trollfjorden–Komagelva Fault Zone onto the Finnmark Platform. These models involve a continuation of the Trollfjorden–Komagelva Fault Zone as a WNW–ESE-striking brittle fault (e.g., Gabrielsen, 1984; Gabrielsen & Færseth, 1989; Roberts *et al.*, 2011), a truncation of the Trollfjorden–Komagelva Fault Zone by a major northwest-dipping Caledonian shear zone (the Sørøya–Ingøya shear zone; Koehl *et al.*, 2018a), and that the Trollfjorden–Komagelva Fault Zone dies out westwards in Magerøya ( Koehl *et al.*, 2019).

The present study has implications for the onshore–offshore correlation of tectonic structures in integrated studies and for the interpretation of major thrusts consisting of ductile shear zones and brittle faults on seismic data. Other implications include a reevaluation of the meaning of “affinities” of specific nappe units with specific plates, the tectonic setting of formation of magmatic intrusions in northern Norway, and the relationship between the opening of the Iapetus Ocean and the Timanian Orogeny in the late Neoproterozoic. Notably, the present work discusses the origin of the Seiland Igneous Province and of the Kalak Nappe Complex ( Figure 1a–b), which were formerly thought to represent intrusions related to the rifting of the Iapetus Ocean and an exotic terrane with Laurentian affinities respectively. The present work also briefly discusses the Porsanger Orogeny and the Finnmarkian event. The former is a poorly constrained Neoproterozoic episode of contraction in northern Norway, whereas the latter was previously believed to be an early phase of early Cambrian of Caledonian deformation. We summarize our findings and their implications in a model detailing the geological evolution of northern Norway from the latest Mesoproterozoic to the mid-Paleozoic. The present work may be used as a framework for new plate tectonic reconstructions for the Neoproterozoic–Paleozoic and for tectonic models in regions deformed by two or more orogenic events.

## Geological setting

### Grenvillian-Sveconorwegian and Porsanger orogenies

**Figure 2.**
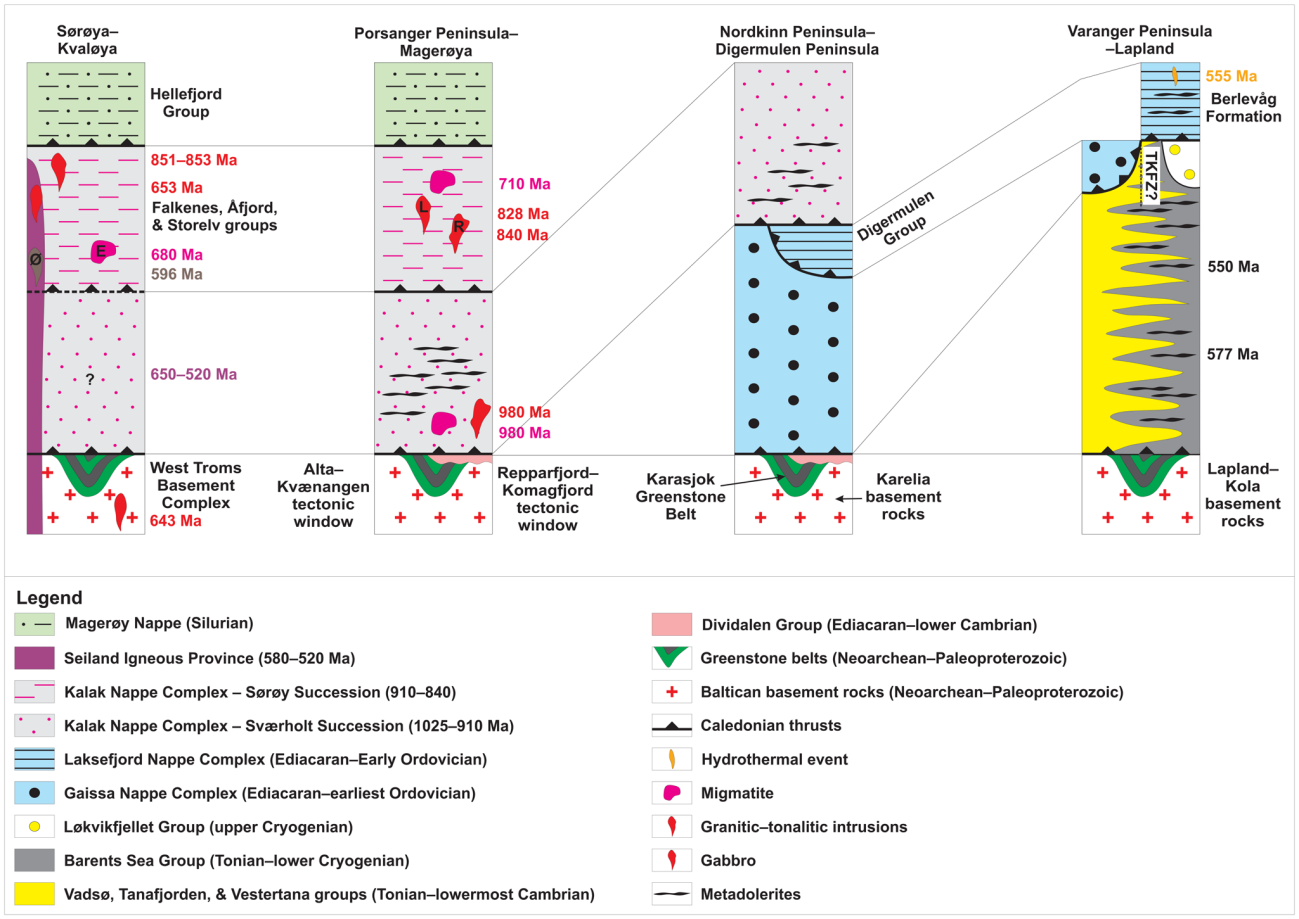
Tectonostratigraphic chart and regional correlations in the study area. Abbreviations: E: Eidvågeid migmatite; L: Lillefjord Granite; R: Revsneshamn Granite; Ø: Øksfjord Gabbro.

Tectonothermal and magmatic activity of latest Mesoproterozoic–earliest Neoproterozoic age was recorded in all three of Svalbard’s basement terranes through various geochronological studies of granites, gneisses, and schists and suggests that Svalbard was involved in the Grenvillian Orogeny ( Hellmann *et al.*, 1998; Johansson & Gee, 1999; Johansson *et al.*, 2000; Johansson *et al.*, 2001; Johansson *et al.*, 2004; Johansson *et al.*, 2005; Lorenz *et al.*, 2012; McClelland *et al.*, 2018; Ohta & Larionov, 1998; Ohta *et al.*, 2003; Pettersson *et al.*, 2009a; Pettersson *et al.*, 2009b; Sirotkin & Evdokimov, 2022). A similar record in southern Norway and southern Sweden indicate that southern Scandinavia was involved in a comparable event, the Sveconorwegian Orogeny ( Andersen *et al.*, 2007; Bingen *et al.*, 2008; Slagstad *et al.*, 2013; Viola *et al.*, 2011). In northern Norway however, this tectonothermal and magmatic activity is recorded exclusively in blocks and terranes thought to be exotic to Baltica ( Agyei-Dwarko *et al.*, 2012; Augland *et al.*, 2013), e.g., Kalak Nappe Complex ( Daly *et al.*, 1991; Kirkland *et al.*, 2006a; Figure 1b), whose deposition dated to > 980 Ma (lower part; Kirkland *et al.*, 2006a) and ca. 920–910 ± 15–20 Ma (upper part; Kirkland *et al.*, 2007a) overlaps with the Grenvillian–Sveconorwegian event ( Figure 2).

The Porsanger Orogeny is a contractional event in northern Norway ( Corfu *et al.*, 2007; Daly *et al.*, 1991; Kirkland *et al.*, 2006a) and is connected to the intrusion of granites (e.g., Lillefjord and Revsneshamn granites; Figure 1b) and pegmatites dated respectively to 840± 6 Ma and 828 ± 4 Ma through U–Pb geochronological analysis of zircon ( Kirkland *et al.*, 2006a; Figure 2). The granites intrude Mesoproterozoic–Tonian metasedimentary rocks of the Kalak Nappe Complex, which show Laurentian affinities ( Corfu *et al.*, 2007; Kirkland *et al.*, 2007a; Slagstad *et al.*, 2006; Figure 2). Note that similar 870–840 Ma ages were recently obtained for granitic and gabbroic magmatic suites in northern Sweden, but that these were correlated with the early breakup of Rodinia ( Callegari *et al.*, 2023).

### Timanian Orogeny

The Timanian Orogeny is a major episode of SSW-dipping subduction and continental accretion that occurred on the northern rim of the Baltican craton in the late Neoproterozoic (ca. 650–550 Ma; Dovzhikova *et al.*, 2004; Gee *et al.*, 2000; Glodny *et al.*, 2004; Larionov *et al.*, 2004; Olovyanishnikov *et al.*, 2000; Pease *et al.*, 2004; Remizov & Pease, 2004; Remizov, 2006; Figure 1a). The orogeny led to a succession of magmatic and tectonic events, which resulted in the formation of a major fold-and-thrust belt characterized dominantly by NNE-dipping thrusts and SSW-verging folds (e.g., Central and West Timan faults; Korago *et al.*, 2004; Kostyuchenko *et al.*, 2006; Lopatin *et al.*, 2001; Lorenz *et al.*, 2004; Olovyanishnikov *et al.*, 2000; Figure 1a), deformation reaching blueschist and eclogite facies metamorphism ( Beckholmen & Glodny, 2004; Glodny *et al.*, 2004; Remizov & Pease, 2004; Remizov, 2006), and the intrusion of various magmatic suites, including subduction-related island- to continental-arc suites ( Dovzhikova *et al.*, 2004; Glodny *et al.*, 2004; Remizov & Pease, 2004; Remizov, 2006), and syn–late-orogenic/subduction calc-alkaline suites (ca. 700–515 Ma; Dovzhikova *et al.*, 2004; Gee *et al.*, 2000; Larionov *et al.*, 2004; Pease *et al.*, 2004) and elongated, post-orogenic alkaline suites (ca. 565–500 Ma; Kuznetsov *et al.*, 2007; Larionov *et al.*, 2004).

In northern Norway, remnants of the Timanian Orogeny are found on the Varanger Peninsula in easternmost Finnmark ( Figure 1a–b). There, a major, WNW–ESE-striking, NNE-dipping brittle–ductile fault, the Trollfjorden–Komagelva Fault Zone, represents the southernmost Timanian thrust fault ( Herrevold *et al.*, 2009; Siedlecka, 1975; Figure 1a–b). This fault is thought to have accommodated top-SSW, reverse-sinistral movements in the latest Neoproterozoic (transpression; Herrevold *et al.*, 2009). Later on, it was reactivated during the Caledonian Orogeny (dextral strike-slip to dextral-reverse oblique-slip movements; Herrevold *et al.*, 2009; Roberts, 1972), during post-Caledonian Devonian–Mississippian collapse (normal-dextral strike-slip movements; Koehl *et al.*, 2018a; Koehl *et al.*, 2019; Roberts *et al.*, 2011), and potentially during further episodes of rifting ( Herrevold *et al.*, 2009). Dextral displacement along the Trollfjorden–Komagelva Fault Zone was estimated to 207 kilometers ( Rice, 2014).

East of Finnmark, the Trollfjorden–Komagelva Fault Zone merges with the Sredni–Rybachi Fault Zone on the Sredni and Rybachi peninsulas in northwestern Russia, and continues farther east into the Timan Range as the Central Timan Fault and/or West Timan Fault ( Olovyanishnikov *et al.*, 2000; Figure 1a). West of the Varanger Peninsula, the Trollfjorden–Komagelva Fault Zone is thought to proceed between the mainland and the Nordkinn Peninsula and off the northern tip of the Sværholt Peninsula.

Farther west, the continuation of the Trollfjorden–Komagelva Fault Zone is more debated and several models were proposed. Interpretation of seismic data along the northern coast of Finnmark by Gabrielsen (1984) and Roberts *et al.* (2011) suggests that the Trollfjorden–Komagelva Fault Zone continues north of Magerøya and merges with the NW–SE-striking, northeast-dipping segment of the Troms–Finnmark Fault Complex, a major post-Caledonian, overall northwest-dipping fault that bounds Paleozoic–Cenozoic sedimentary basins ( Gabrielsen *et al.*, 1990; Figure 1b). However, recent analysis of 2D and 3D seismic data suggest that the Trollfjorden–Komagelva Fault Zone does not merge with the Troms–Finnmark Fault Complex and is, instead, truncated by a major, several kilometers thick, northwest-dipping Caledonian shear zone, the Sørøya–Ingøya shear zone ( Koehl *et al.*, 2018a; Figure 1b). Another model based on the interpretation of high-resolution bathymetric and magnetic data in northern Finnmark suggests that the Trollfjorden–Komagelva Fault Zone splays into multiple minor fault segments westwards on the island of Magerøya and dies out just west of the coastline ( Koehl *et al.*, 2019).

### Late Neoproterozoic–Cambrian magmatism in Northern Norway

Mafic–ultramafic and alkaline rocks of the Seiland Igneous Province ( Krauskopf, 1954; Robins & Gardner, 1975; Speedyman, 1983; Sturt & Ramsay, 1965; Figure 1a–b) intruded rocks of the Kalak Nappe Complex at ca. 580–560 Ma and 530–520 Ma ( Krill & Zwaan, 1987; Roberts *et al.*, 2006; Roberts *et al.*, 2010; Figure 2). Initially, folds crosscut by these intrusions were thought to reflect early Caledonian contraction (Finnmarkian Orogeny; e.g., Kirkland *et al.*, 2006a; Kirkland *et al.*, 2007a; Kirkland *et al.*, 2008a; Sturt *et al.*, 1978). The folds are now known to be of magmatic origin and related to dyke intrusion (e.g., Krill & Zwaan, 1987). The proximity of the Seiland Igneous Province with the Iapetus paleo-margin and reconstruction of intruded rocks of the Kalak Nappe Complex within the Iapetus Ocean suggest that the Seiland Igneous Province is related to rifting of Iapetus ( Bergström & Gee, 1985; Elvevold *et al.*, 1994; Kirkland *et al.*, 2008b; Krill & Zwaan, 1987; Larsen *et al.*, 2018). However, geochemical analyzes indicate that (back) arc and collisional settings are also possible, as also suggested by earlier petrochemical analyses ( Roberts, 1975; Roberts *et al.*, 2006; Roberts *et al.*, 2010; Robins & Gardner, 1975; Speedyman, 1983).

In Varangerhalvøya, the Berlevåg Formation, which is commonly thought to be of Baltican origin, was affected by a 555 ± 15 Ma hydrothermal event ( Kirkland *et al.*, 2008b; Figure 1b and Figure 2). This event is potentially related to the intrusion of 577 ± 14 Ma and 550 ± 7.3 Ma metadolerite dykes in northern Varangerhalvøya ( Andersen & Sundvoll, 1995; Rice *et al.*, 2004). These dykes were ascribed to the opening of the Iapetus Ocean, though a backarc setting is equally as possible (Alexander Hugh Rice pers. comm., 2022; Rice *et al.*, 2004). They are depicted by recent aeromagnetic data on the Varanger and Nordkinn peninsulas ( Nasuti *et al.*, 2015a), and therefore intrude both rocks of the Barents Sea Group and of the Kalak Nappe Complex ( Figure 1b). Farther west, on the Porsanger Peninsula, the Kalak Nappe Complex is intruded by (boudinaged) dolerite sills, which were metamorphosed during the Caledonian Orogeny, thus suggesting a Precambrian, possibly Ediacaran, age for the intrusions (David Roberts pers. comm., 2022; Roberts, 1987; Figure 2).

### Caledonian Orogeny

In the early Paleozoic, the Caledonian Orogeny led to the closing of the Iapetus Ocean and the collision of Baltica (including Svalbard and the Barents Sea; Koehl *et al.*, 2022a; Koehl *et al.*, 2023) and Laurentia ( Corfu *et al.*, 2014). In northern Norway (Troms and Finnmark), this orogeny resulted in the formation of overall top-southeast thrusts and southeast-verging folds ( Gayer *et al.*, 1985; Townsend *et al.*, 1986; Townsend, 1987), which thrust Caledonian nappes onto Neoarchean–Paleoproterozoic basement rocks ( Bergh & Torske, 1988; Bergh *et al.*, 2010; Pharaoh *et al.*, 1982; Pharaoh *et al.*, 1983; Reitan, 1963; Zwaan, 1995; Figure 1b). In places, Caledonian metamorphism reached eclogite facies (e.g., Tromsø Nappe; Corfu *et al.*, 2003; Ravna & Roux, 2006).

In northern Norway, the Caledonian Orogeny was initially thought to have initiated with a first event at ca. 540–480 Ma, the Finnmarkian Orogeny ( Roberts, 2003; Sturt *et al.*, 1978; Torsvik & Rehnström, 2001). This event was later revised and downgraded to a short-lived accretion event because of inconsistencies in structural relationships around the intrusion of the Seiland Igneous Province ( Krill & Zwaan, 1987), lack of corresponding major structures, and partial resetting of geochronometers ( Kirkland *et al.*, 2006a; Kirkland *et al.*, 2008b).

In the latest Ordovician to Silurian, the collision of Greenland and Norway during the Scandian phase of the Caledonian Orogeny resulted in intense deformation and up to eclogite-facies metamorphism ( Augland *et al.*, 2013; Corfu *et al.*, 2003; Corfu *et al.*, 2006; Faber *et al.*, 2019; Gaidies *et al.*, 2021; Gasser *et al.*, 2015; Kirkland *et al.*, 2006b; Kirkland *et al.*, 2007b; Ravna & Roux, 2006; Slagstad *et al.*, 2020; Ziemniak *et al.*, 2019). Coevally, Silurian sedimentary rocks of the Magerøy Nappe ( Henningsmoen, 1961) were deposited, thrust, and metamorphosed mostly to greenschist facies ( Andersen, 1981; Andersen, 1984; Figure 1b; Figure 2). Deformation culminated at ca. 425–420 Ma prior to the collapse of the Caledonian Orogen ( Kirkland *et al.*, 2006b).

During Caledonian deformation, Timanian thrusts and folds were reworked into dominantly NNE-plunging, 3D dome- and trough-shaped structures, both in the Barents Sea and Svalbard ( Koehl *et al.*, 2022a; Koehl *et al.*, 2023), and in the Varanger Peninsula ( Gabrielsen *et al.*, 2022; Siedlecka & Siedlecki, 1971). In addition, the arrangement of fault segments in duplex structures in map view suggests that the segment of the Trollfjorden–Komagelva Fault Zone on the Varanger Peninsula was also partly reworked as a strike-slip fault by Caledonian deformation ( Herrevold *et al.*, 2009; Siedlecka & Siedlecki, 1967; Siedlecka, 1975).

### Post-Caledonian extension

In the Devonian–Carboniferous, the Caledonian Orogen started to collapse, which resulted in the formation of brittle normal faults along preexisting zones of weakness and reactivation of Caledonian thrusts (e.g., Koehl *et al.*, 2018b; Torgersen *et al.*, 2014; Figure 1b). For example, Neoproterozoic metasedimentary rocks of the Barents Sea and Tanafjorden–Varangerbotn regions were intruded respectively by NE–SW- to ENE–WSW- and N–S-striking Late Devonian dykes, which intruded along brittle faults in the eastern part of the Varanger Peninsula ( Guise & Roberts, 2002; Nasuti *et al.*, 2015a; Roberts, 2011) and adjacent area in Russia ( Roberts & Onstott, 1995). Analogously, Neoproterozoic–Silurian metasedimentary rocks of the Kalak Nappe Complex and Magerøy Nappe and Neoarchean–Paleoproterozoic basement rocks were truncated by WNW–ESE-striking brittle faults along which Mississippian dolerite dykes were intruded in Magerøya ( Lippard & Prestvik, 1997; Roberts *et al.*, 1991) and adjacent areas in Finnmark ( Braathen & Davidsen, 2000; Koehl *et al.*, 2019; Nasuti *et al.*, 2015a).

In northwestern Finnmark, Devonian–Carboniferous post–late-Caledonian extensional faulting is marked by fault complexes consisting of alternating ENE–WSW- and NNE–SSW-striking segments (e.g., Langfjorden–Vargsund fault complex; Koehl *et al.*, 2019; Lippard & Roberts, 1987; Roberts & Lippard, 2005; Figure 1b). These fault complexes also developed along preexisting folds and thrusts in Paleoproterozoic basement rocks ( Henderson *et al.*, 2015; Koehl *et al.*, 2019).

## Methods and data

**Figure 3.**
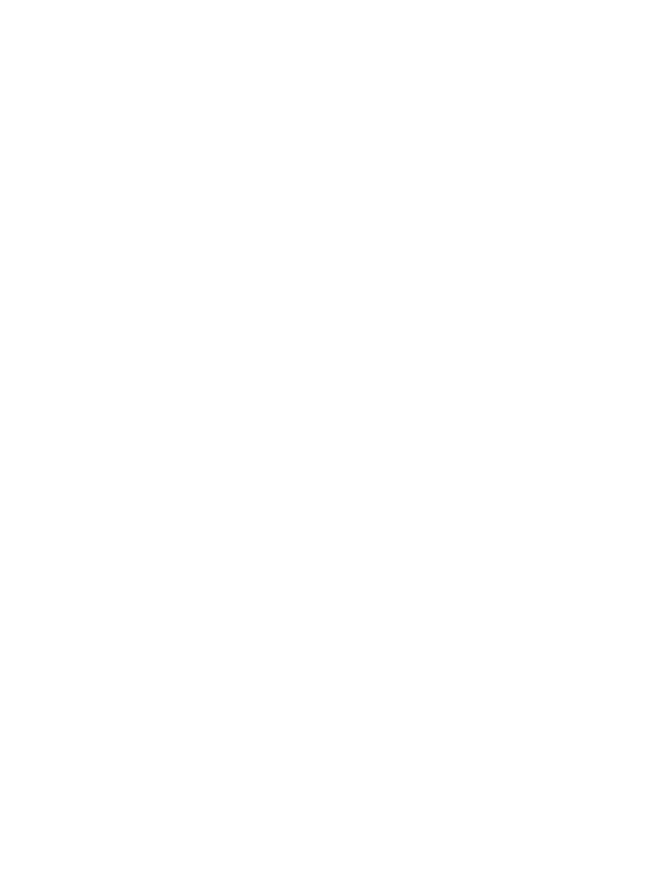
Overview of the ( **a**) 2D and ( **b**) 3D seismic reflection database uses in the present study. The maps also display the depth surfaces (in milliseconds TWT) of the main two Timanian thrusts mapped on the Finnmark Platform.

**Figure 4.**
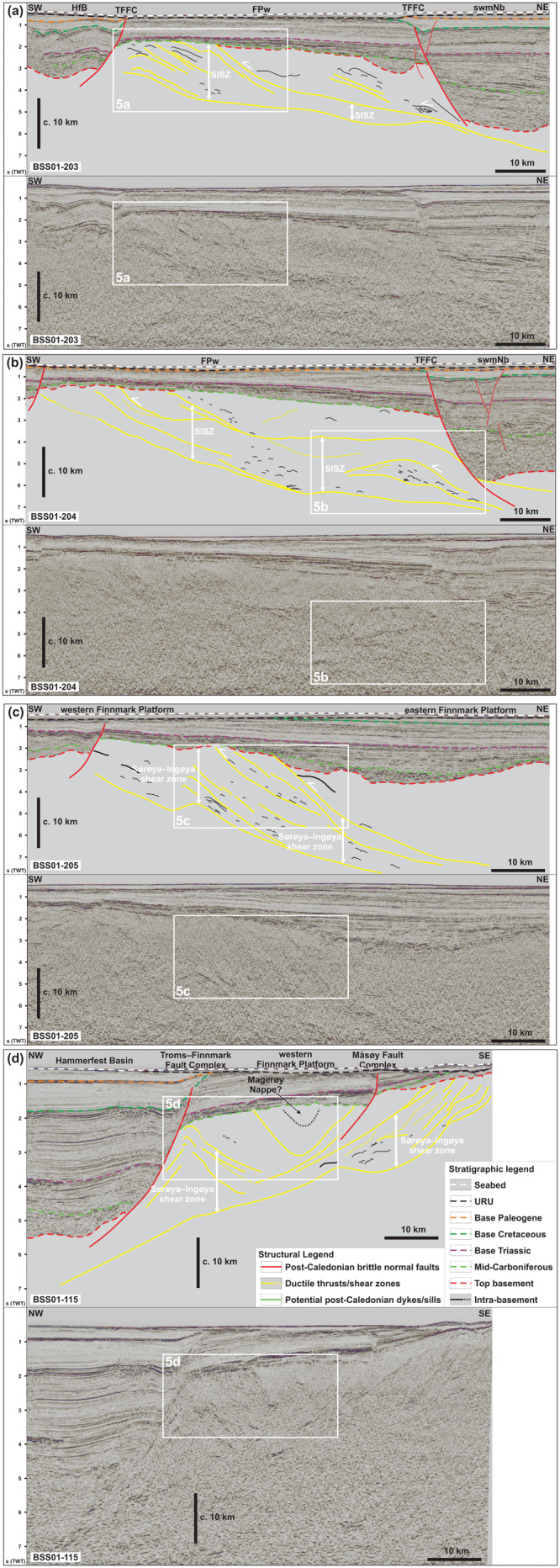
Interpreted (up) and uninterpreted (down) seismic reflection data on the western Finnmark Platform. The location of the data is shown in Figure 7. ( **a**– **c**) NE–SW-trending seismic lines illustrating the moderately NNE-dipping geometry of the Sørøya–Ingøya shear zone. ( **d**) NW–SE-trending seismic line showing major thickness variations of the Sørøya–Ingøya shear zone related to Devonian–Carboniferous core complex exhumation ( Koehl *et al.*, 2018a) and to Caledonian reworking into NE–SW-striking folds.

**Figure 5.**
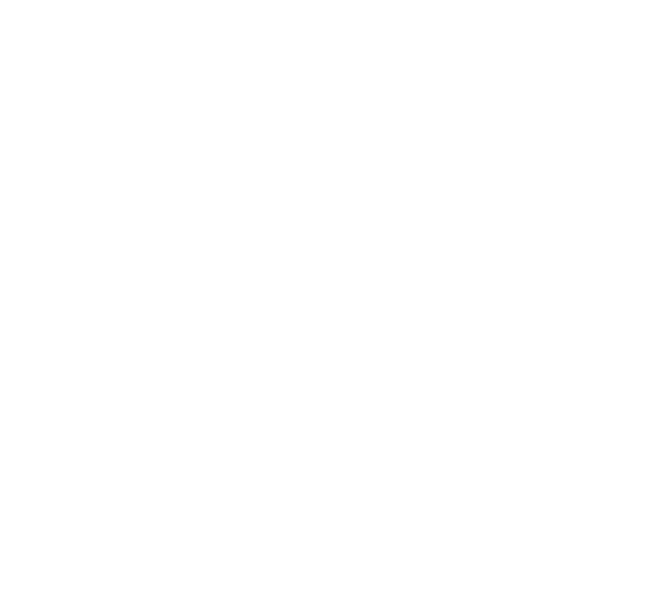
Zoom in interpreted (up) and uninterpreted (down) seismic data on the western Finnmark Platform. The location of the data is shown in Figure 6. ( **a**) Zoom in a NE–SW-trending seismic line showing numerous SSW-verging folds and associated brittle thrusts with top-SSW offsets within the western portion of the Sørøya–Ingøya shear zone. Triangular-shaped packages in the upper left corner are interpreted as deformed foreland basin deposits. ( **b**) Zoom in a NE–SW-trending seismic line showing sigmoidal packages of Z-shaped reflections respectively interpreted as antiformal thrust stacks and duplexes within the Sørøya–Ingøya shear zone. ( **c**) Zoom in a NE–SW-trending seismic line showing the eastern portion of the Sørøya–Ingøya shear zone, which bends back into a NNE-dipping orientation. Most folds within the shear zone display a vergence to the south-southwest indicating top-SSW movements. ( **d**) Zoom in a NW–SE-trending seismic line showing symmetric folds in shallow basement rocks in a major NE–SW-striking syncline possibly consisting of rocks equivalent to the Magerøy Nappe (MN; upper right corner), northwest-verging folds in the northwestern limb of the major syncline (left hand-side), and southeast-verging fold structures in the southeastern limb of the major syncline (lower right corner).

The present study uses 2D and 3D seismic reflection data from the Norwegian National Data Repository for Petroleum Data (DISKOS database), 2D data from the Norwegian Defense Research Establishment, and 3D data from TGS (FP13; Figure 3) to map hundreds of meters to tens of kilometers large structures offshore ( Figure 4a–b, Figure 5– Figure 7; Koehl, 2024). Notably, the study highlights major shear zones and thrusts and related folds on the Finnmark Platform, off the coast of northern Norway ( Figure 1a–b). The acquisition and reprocessing reports document that the smaller (hundreds of meters wide) structures reported by the present study are well within the horizontal and vertical resolution of the seismic data analyzed.

**Figure 6.**
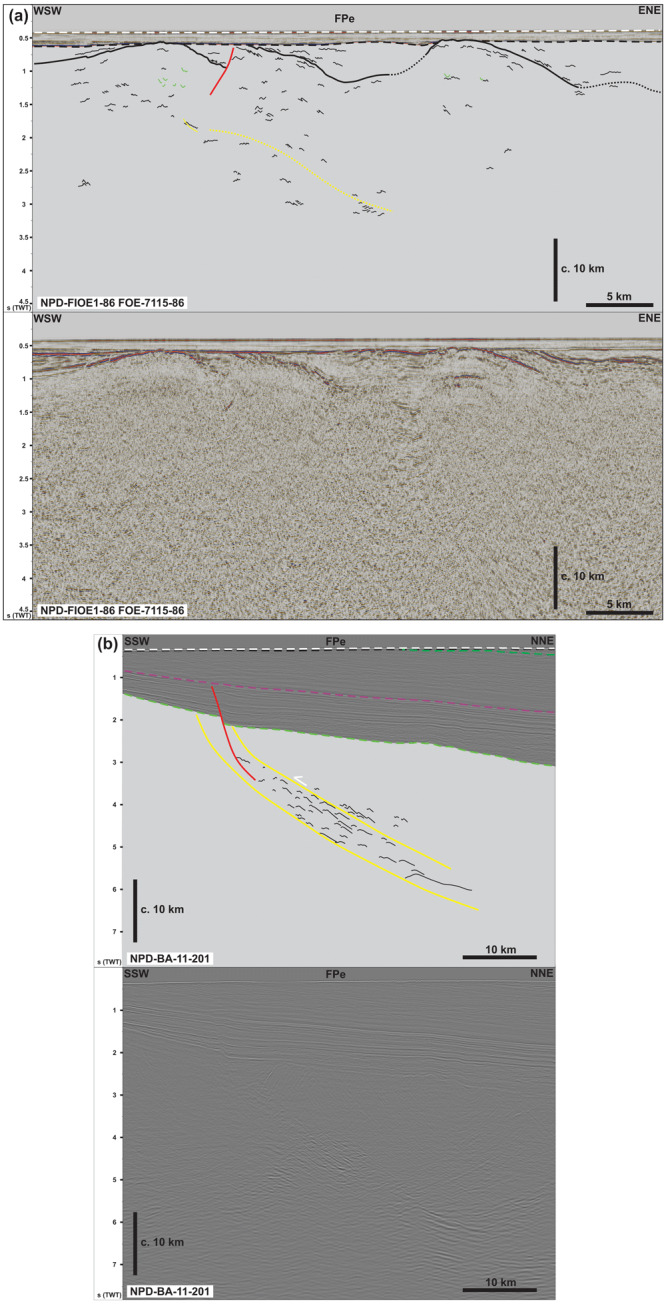
Interpreted (up) and uninterpreted (down) seismic reflection data on the eastern Finnmark Platform. The location of the data is shown in Figure 7. ( **a**) Intra-basement N–S- to NE–SW-striking Caledonian folds. The data show dominantly symmetric folds. Note that the URU reflection coincides with the Top-basement reflection here. ( **b**) NNE-dipping ductile shear zone consisting of planar mylonitic surfaces and SSW-verging folds indicating transport direction towards the south-southwest. Notice the mild reactivation of the shallow portion of the thrust by a late Paleozoic listric normal fault. A different color scheme was used to enhance the contrast and better highlight the structures described.

**Figure 7.**
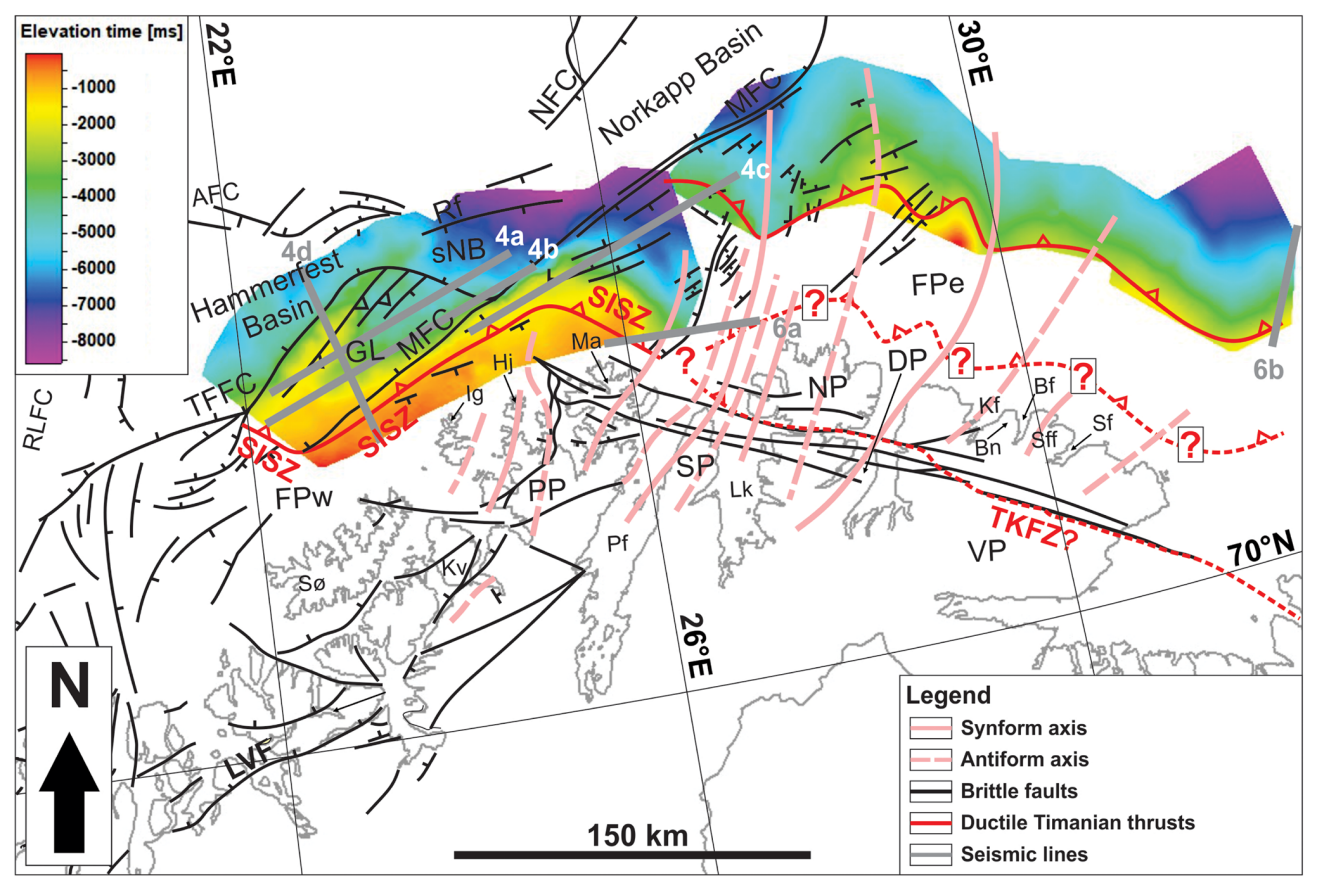
Map showing the depth surfaces (in milliseconds TWT) of the main two Timanian thrusts on the Finnmark Platform. Notice the correlation of major synforms and antiforms observed in the field onshore and on onshore–offshore magnetic data (plain and dashed pink lines) with folding of the Timanian thrusts on the Finnmark Platform in map view identified via seismic mapping (depth surfaces). The map includes major faults from Figure 1. For abbreviations, see Figure 1b.

The vertical resolution of the survey is ¼ of the wavelength, which is computed from the velocity of basement rocks in the study area, i.e., 6300 m.s-1 (from Gernigon *et al.*, 2018), and the frequency of the data, i.e., c. 40 Hz ( TGS, 2014 their Figure 12). In the present case, the vertical resolution of the data is c. 39 m (= 6300/40/4; Table 1). Note that this is a minimum estimate since a bandwidth enhancement was attempted with frequencies up to c. 60 Hz ( TGS, 2014 their Figure 13), i.e., an improved resolution of c. 26 m ( Table 1). The regularization stage of the reprocessing summary for 3D survey FP13 reports a bin size of 12.5 m x 25 m, i.e., a Fresnel Zone radius of c. 313 m ( TGS, 2014), which corresponds to the horizontal resolution of the survey at shallow depth. Deeper in the crust, the horizontal resolution worsens and is a function of depth and the wavelength ( Geldart & Sheriff, 2004). For example, the horizontal resolution of the survey at 5000 m depth is c. 627 m (= (5000 x (6300/40)/2) ^1/2^); Table 1).

The seismic data of survey BSS01 at shallow (0–3500 m depth) and high depth (3500–8000 m depth) respectively contain frequencies up to 60–80 Hz and 40 Hz ( TGS-NOPEC, 2001). A minimum estimate of the vertical resolutions at shallow (using 60 Hz frequency) and high depth (using 40 Hz frequency) are therefore c. 26 m and c. 39 m. The bin size of 2D survey BSS01 is 12.5 m x 25 m (respectively the cable group length and the shot point interval; TGS-NOPEC, 2001), hence giving a horizontal resolution of c. 313 m ( Table 1). Similarly to survey FP13, the horizontal resolution of the BSS01 survey at a depth of 5000 m using a frequency of 40 Hz is c. 627 m ( Table 1).

The structures studied, including notably > 500 m wide and > 150 m thick asymmetric folds are well within the horizontal and vertical resolution, both at shallow and high depth (≥ 5000 m) of the seismic data used in the present work ( Table 1). Noteworthy, the vertical resolution of seismic data may be down to 1/32 of the wavelength in places ( Kallweit & Wood, 1982; Li & Zhu, 2000), i.e., down to c. 5 m for a 40 Hz frequency (= 6300/40/32), thus further supporting the interpretation presented. The water depth in the study area, the Barents Sea, is about 200 m with little variations ( Jakobsson *et al.*, 2012) and, thus, has little influence on the structures mapped. Furthermore, seismic velocities for basement rocks in northern Norway from Gernigon *et al.* (2018) suggest that there is little to no vertical exaggeration in the basement rock interval ( Figure 4a–d and Figure 6a–b). Thus, the geometry of the basement-seated structures described in the present manuscript are in most likelihood similar to their actual geometry.

**Table 1.** Summary of the horizontal (for depths of 0 and 5000 m and using a frequency of 40 Hz) and vertical resolution (for frequencies of 40 and 60 Hz and at a depth of 5000 m) of the main seismic datasets used and a comparison with the size of investigated structures in the present study.

Survey	Horizontal resolution (0 m/5000 m depth)		Vertical resolution (40 Hz/60Hz)	
**FP13**	312.5 m	c. 627.495 m	39.375 m	26.25 m
**BSS01**	312.5 m	c. 627.495 m	39.375 m	26.25 m
**Asymmetric folds**	> 500 m		> 150 m	

The data were interpreted using Petrel (version 2021.3), but can also be interpreted using OpendTect (open source). All the structural direction refers to present-day coordinates.

**Figure 8.**
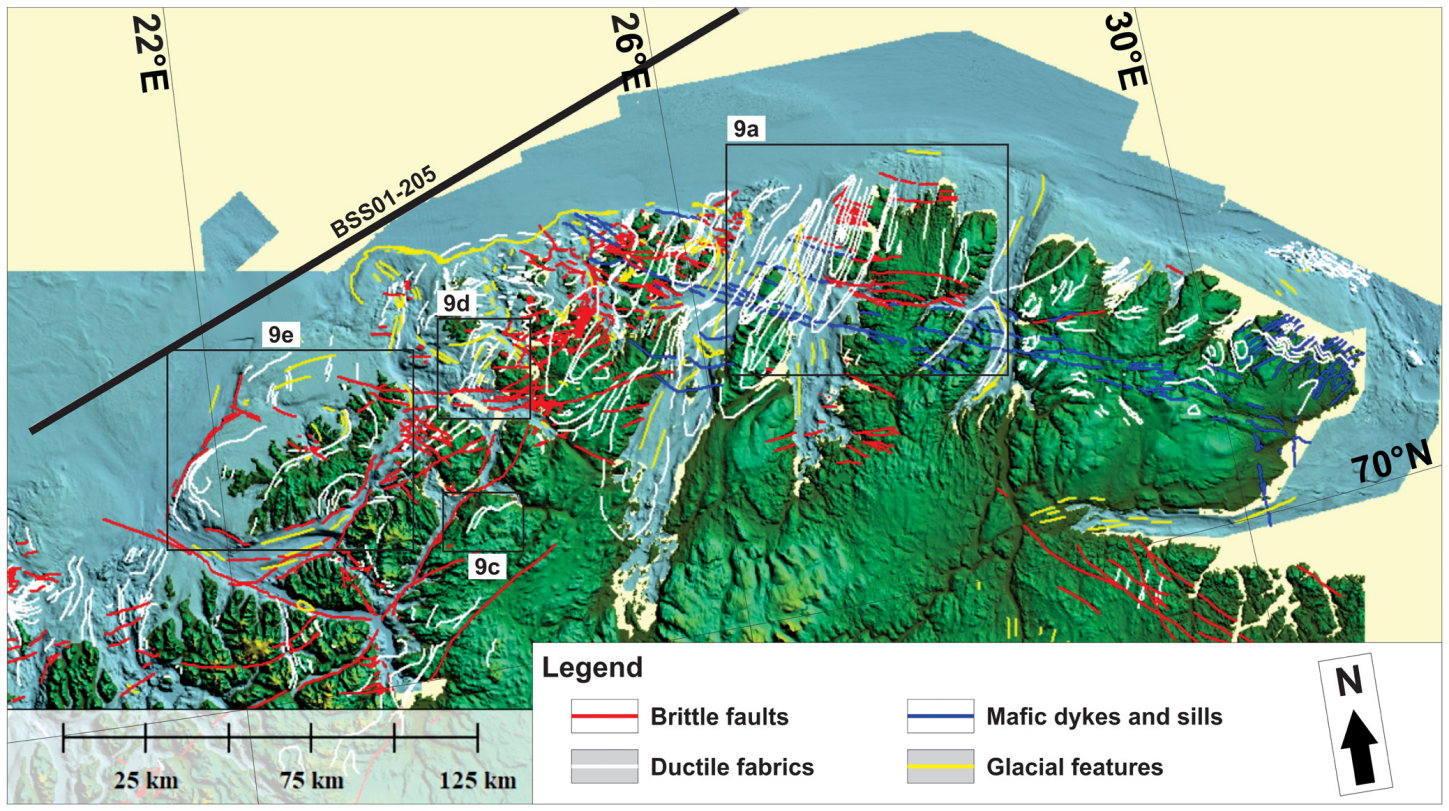
Bathymetric and topographic data onshore Finnmark and nearshore fjords. The map shows major brittle faults, mafic dykes and sills, ductile fabrics (notably folded bedding surfaces), and glacial features. The location of seismic line BSS01-205 ( Figure 4c) is shown as a thick black line. The location of Figure 9a and c–e is shown as black frames.

**Figure 9.**
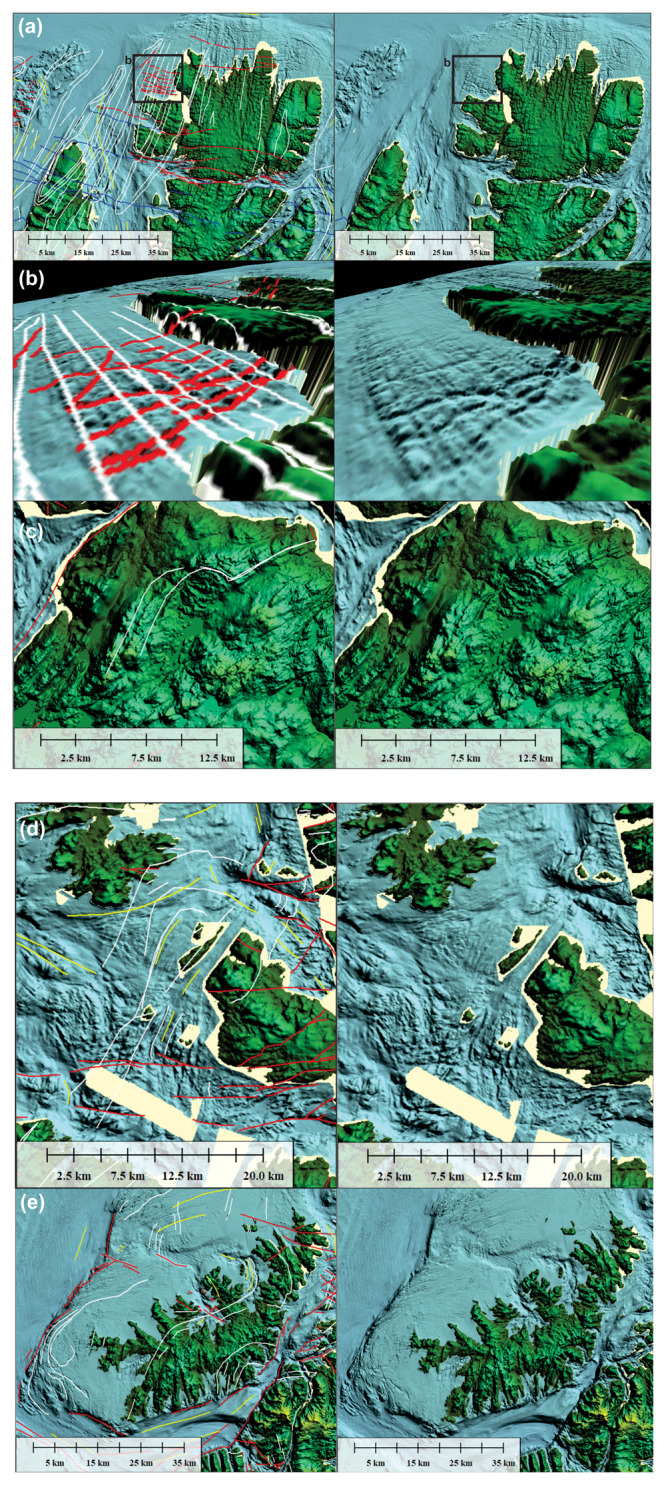
Interpreted (left) and uninterpreted (right) zoom in bathymetric data in ( **a**) the Nordkinn Peninsula, ( **b**) west of the Nordkinn Peninsula in 3D with view towards the northeast, ( **c**) the Repparfjord–Komagfjord tectonic window, ( **d**) southeast of Rolvsøya, and ( **e**) Sørøya. The map shows major brittle faults, mafic dykes and sills, ductile fabrics (notably folded bedding surfaces), and glacial features. The location of seismic line BSS01-205 ( Figure 4c) is shown as a thick black line. The location of Figure9a and c–e is shown as black frames.

Bathymetric and topographic data from the Norwegian Mapping Authority were used to map escarpments and lineaments ( Figure 8 and Figure 9a–e). We further use existing geological maps (e.g., Roberts, 1998; Siedlecki, 1980), structural datasets ( Koehl *et al.*, 2019), glacial feature studies (e.g., Ottesen *et al.*, 2008; Vorren *et al.*, 1986), and Digital Elevation Models such as Norgei3d and GoogleEarth (version 7.3.6.9345) to differentiate glacial features from tectonic structures, including ductile foliation and shear zones, brittle faults, and folds. See also further interpretation and discussion of the bathymetric data in Koehl *et al.* (2019).

**Figure 10.**
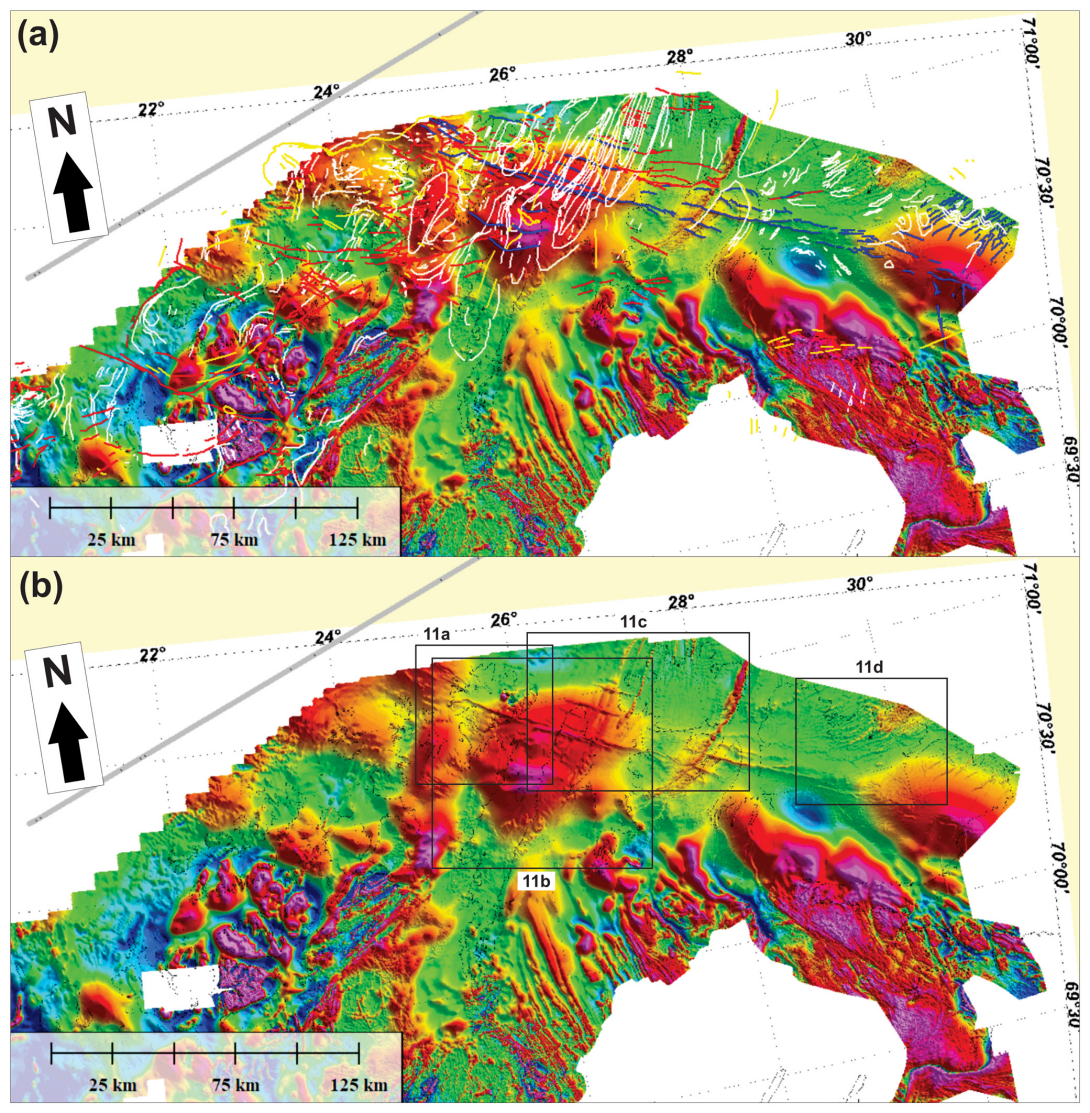
( **a**) Interpreted and ( **b**) uninterpreted magnetic data onshore–nearshore northern Norway. The location of Figure 11a–d is shown as black frames in ( **b**).

**Figure 11.**
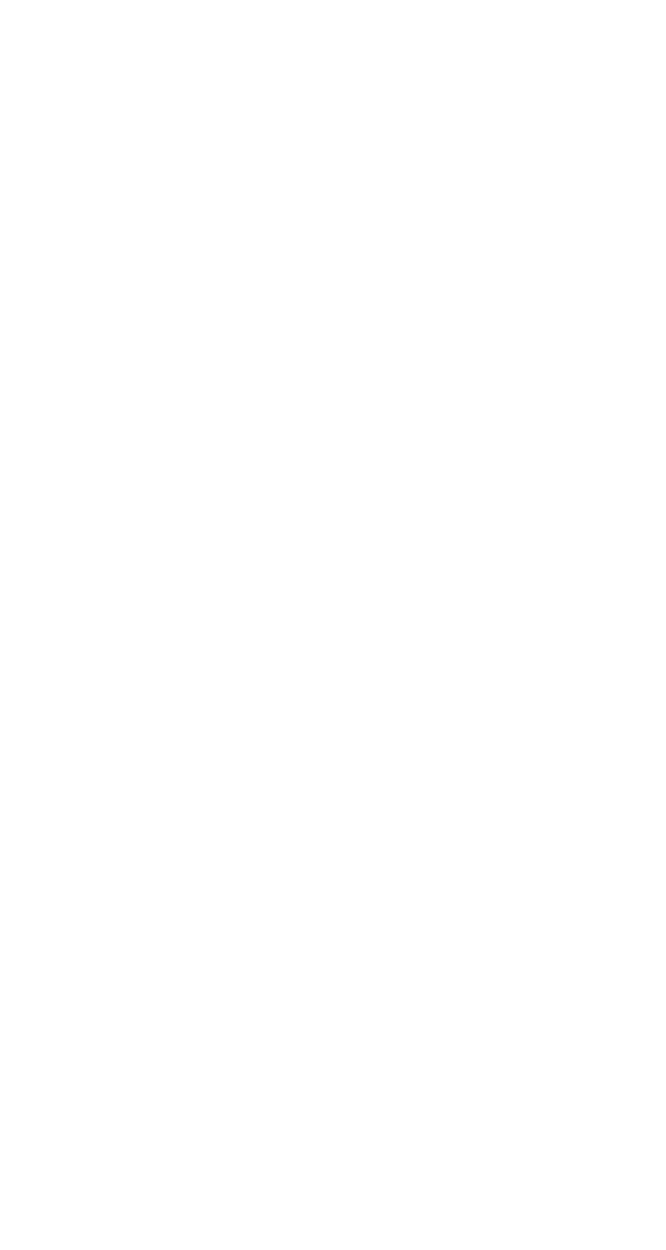
Zoom in interpreted (left) and uninterpreted (right) tilt derivative data from Nasuti *et al.* (2015a) in ( **a**) Magerøya, ( **b**) Porsangerfjorden and the Sværholt Peninsula, ( **c**) the Sværholt Peninsula and Nordkinn Peninsula, and ( **d**) in Syltefjorden and Syltefjordfjellet.

**Figure 12.**
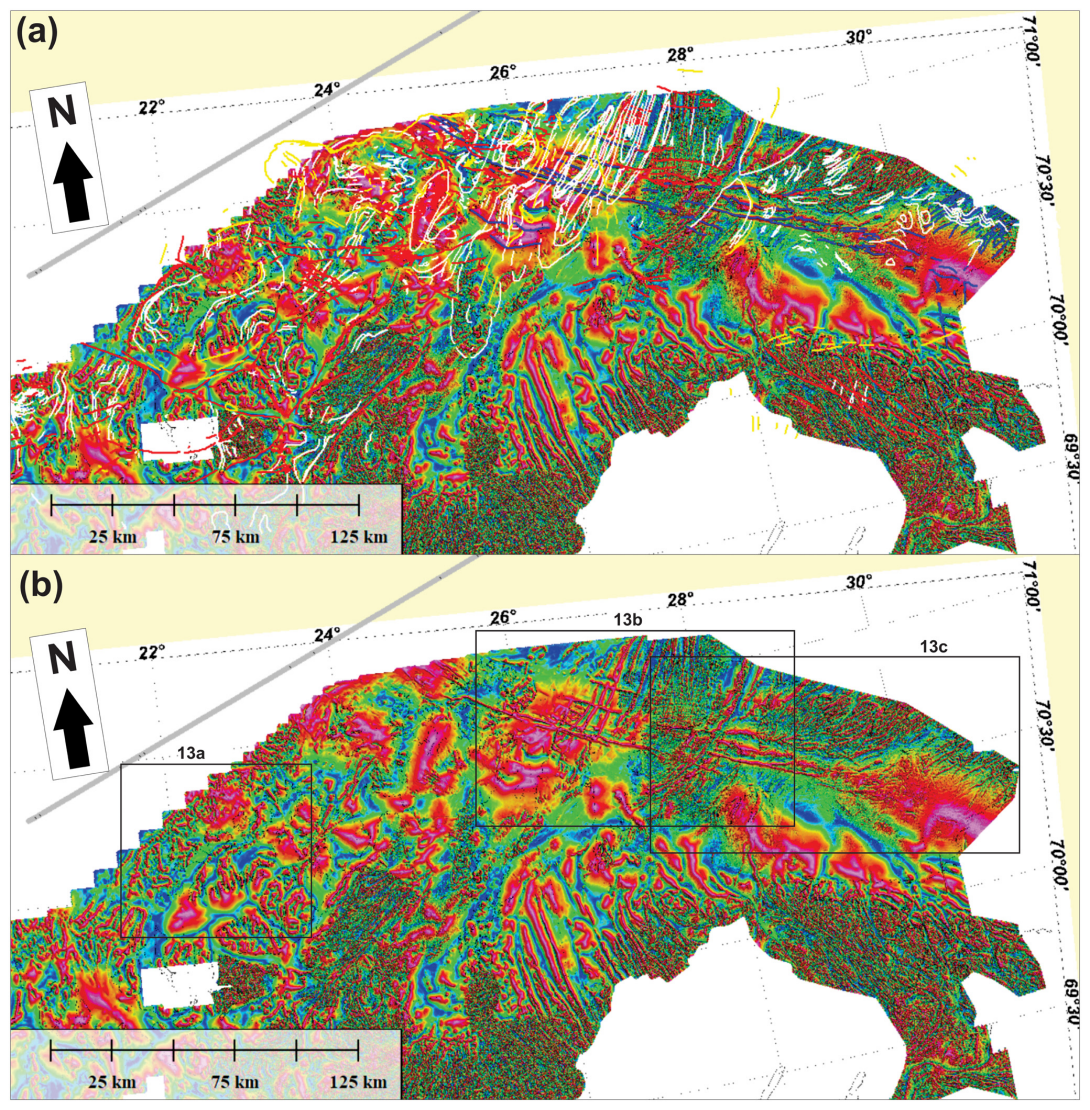
( **a**) Interpreted and ( **b**) uninterpreted tilt-derivative data onshore–nearshore northern Norway. The location of Figure 13a–c is shown as black frames in ( **b**).

**Figure 13.**
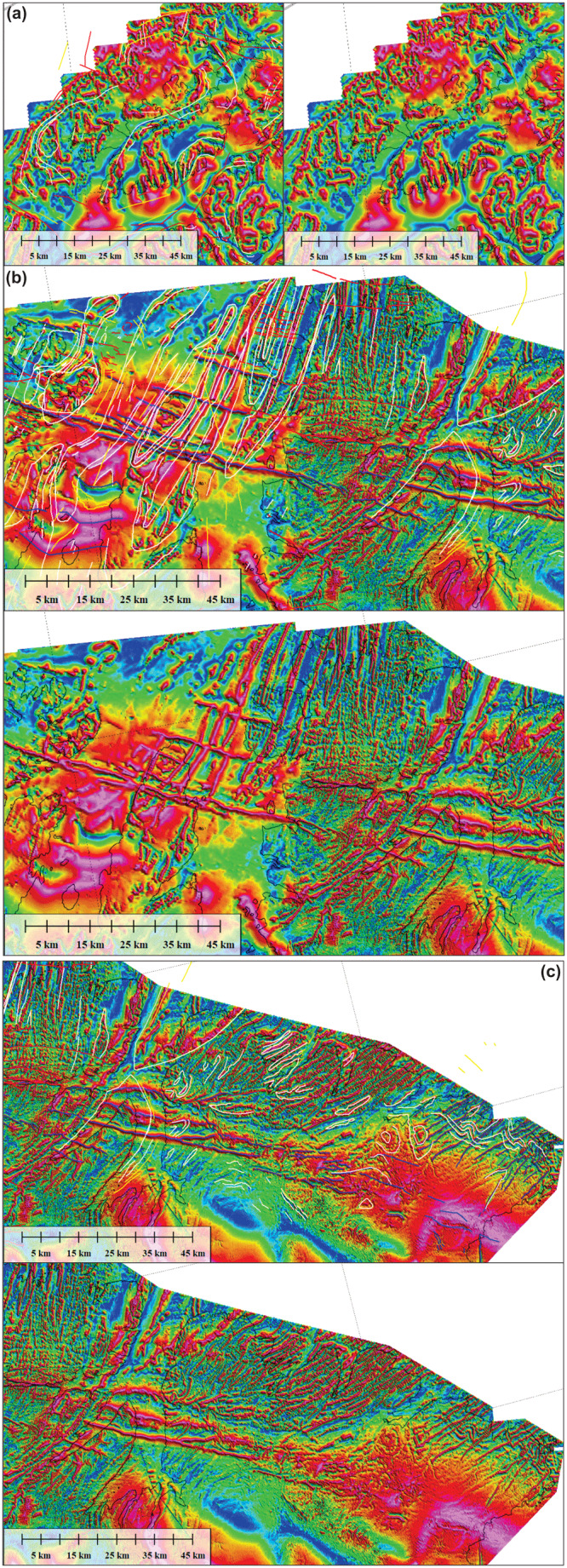
Zoom in interpreted (left in **a** and up in **b** and **c**) and uninterpreted (right in **a** and down in **b** and **c**) tilt derivative data from Nasuti *et al.* (2015a) in ( **a**) Sørøya, ( **b**) Porsangerfjorden, the Sværholt Peninsula, and the Nordkinn Peninsula, and ( **c**) the Digermulen Peninsula and the Varanger Peninsula.

**Figure 14.**
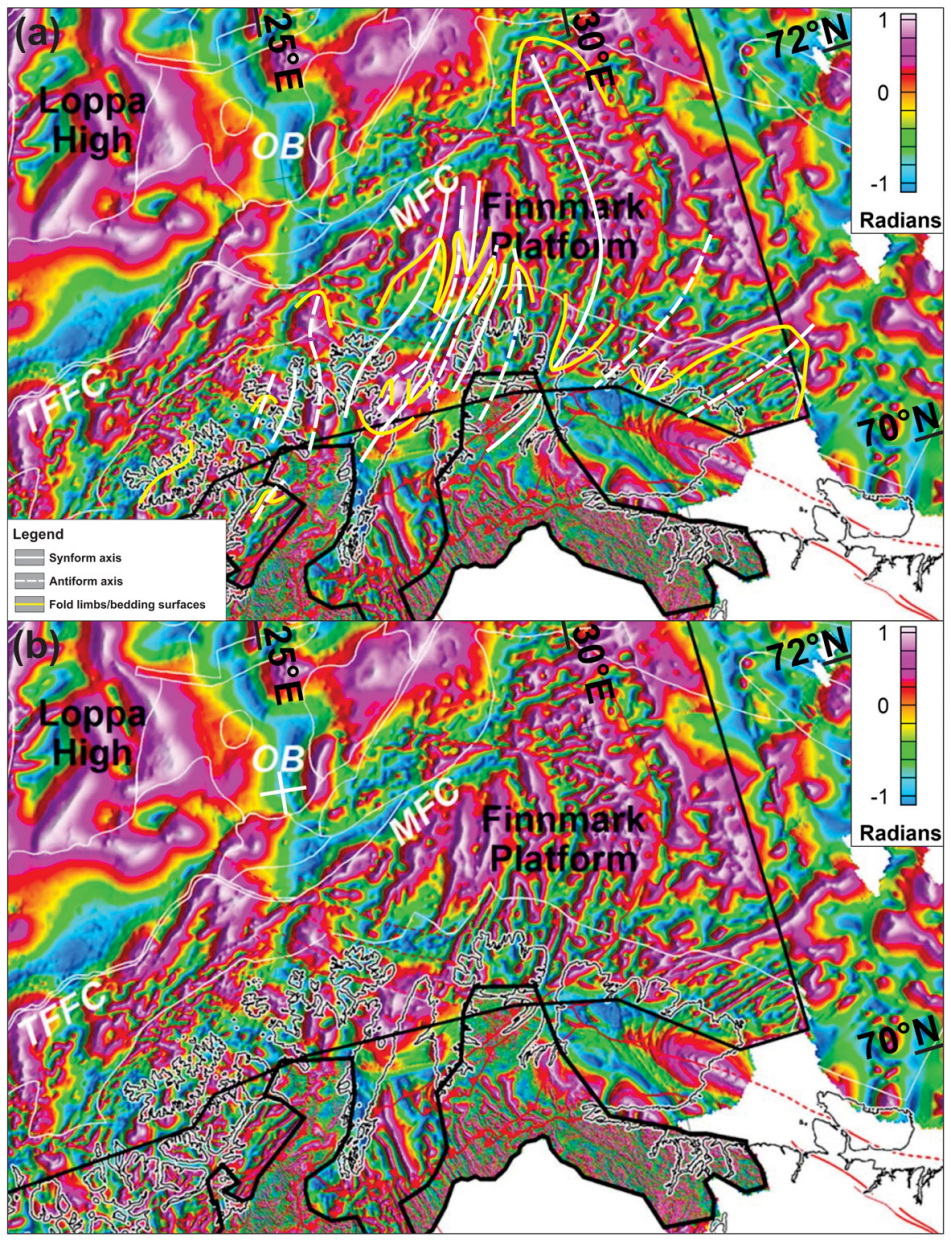
( **a**) Interpreted and ( **b**) uninterpreted tilt-derivative data from Gernigon *et al.* (2014). The data show a correlation between fold structures in the field and on seismic data with magnetic anomalies both onshore northern Norway and on the Finnmark Platform offshore.

**Figure 15.**
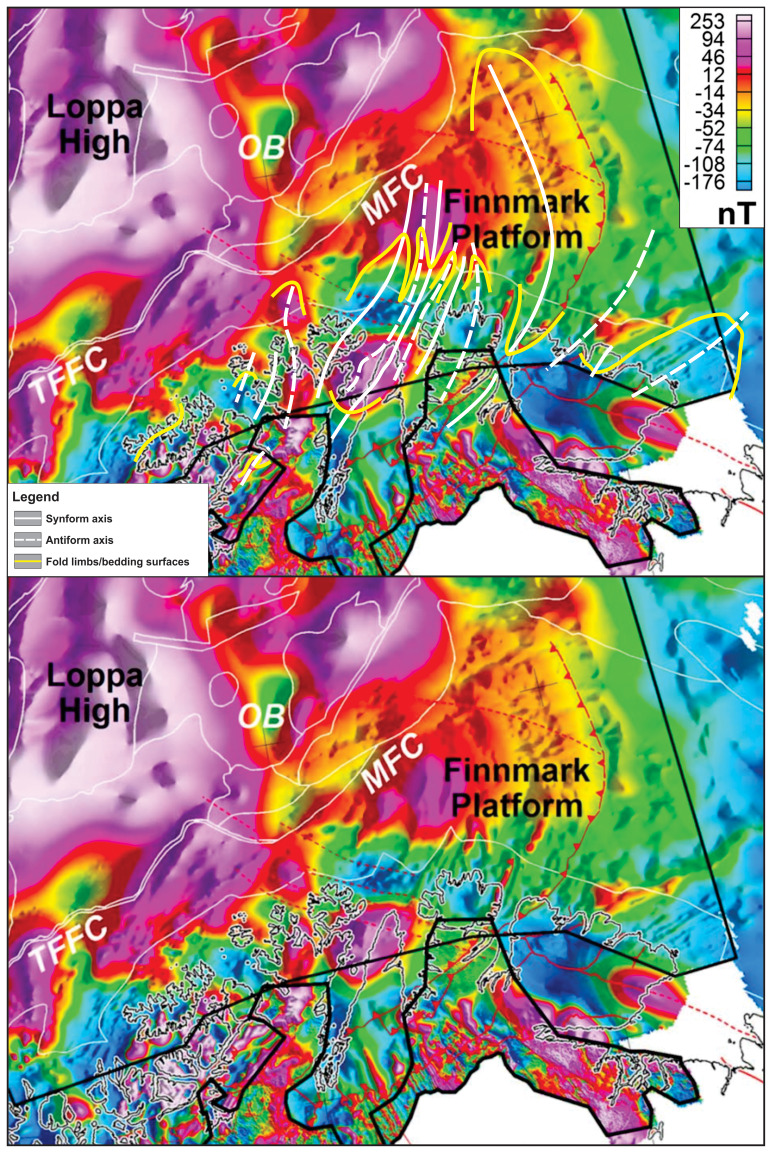
Interpreted (upper inset) and uninterpreted (lower inset) magnetic data from Gernigon *et al.* (2014) showing tens of kilometers wide anomalies interpreted as major folds.

Offshore structures are tied to onshore–nearshore structures using aeromagnetic data (including tilt-derivative) from the Geological Survey of Norway from the MINN ( Nasuti *et al.*, 2015b; Figure 10, Figure 11a–d, Figure 12, and Figure 13a–c; Koehl, 2024) and BASAR datasets ( Gernigon & Brönner, 2012; Gernigon *et al.*, 2014; Figure 14 and Figure 15; Koehl, 2024). Magnetic anomalies are used (1) to infer the presence of magmatic intrusions onshore and nearshore and to study their deformation character (e.g., undulating anomaly reflecting folded, pre-Caledonian dykes and linear anomalies representing post-Caledonian dykes intruded along brittle faults), (2) to map fold structures affecting highly magnetic metasedimentary beds, and (3) to identify highly magnetic rock units extensively intruded by dykes offshore.

Bathymetric, topographic, and magnetic data were interpreted in GlobalMapper (version 13.0), and the figures were designed with CorelDraw (version 2017). Alternative open-source software are QGIS and GIMP respectively.

The structures described and discussed in the present study include post-Caledonian brittle faults and dolerite dykes, and Timanian and Caledonian folds, ductile shear zones, and brittle thrusts. Note that the present study builds on the interpretations of 2D–3D seismic, aeromagnetic, and bathymetric–topographic data published in Koehl *et al.* ( 2018a; 2019). The reader is therefore referred to these works for more information on specific structures not discussed in the present contribution.

## Results

### Seismic data

***Western Finnmark Platform.*** The Sørøya–Ingøya shear zone and its overall geometry are described in Koehl *et al.* (2018a). In map view, the shear zone strikes overall NE–SW with a northwestern dip and bends into a NNE-dipping geometry just northwest of Magerøya ( Figure 1a–b). The shear zone consists of kilometer-thick, gently dipping, planar, moderate- to high-amplitude seismic reflections interpreted as mylonitic fabrics and thrusts ( Koehl *et al.*, 2018a). In the present section, we further highlight its overall geometry and focus on smaller structures within the shear zone.

We further constrained the overall geometry of the Sørøya–Ingøya shear zone to the southwest and identified a bend into a NNE-dipping geometry just southeast of the bend in the Troms–Finnmark Fault Complex in addition to the bend into a NNE-dipping geometry northwest of Magerøya ( Figure 1a–b and Figure 4a–c). In the northwest, southwest, and southeast, the shear zone is truncated by post-Caledonian faults such as the Troms–Finnmark and Måsøy fault complexes ( Figure 4a–d) indicating it is pre-Devonian. The shear zone is up to 3.0 seconds (TWT; i.e., possibly tens of kilometers) thick and dips moderately (c. 30–40°) to the north-northeast in the southwest and more gently (c. 10–15°) in the northeast in NE–SW-trending cross section ( Figure 4a–c), whereas it displays a curving up and down geometry with greater dip angle with portions displaying a dome-shaped geometries in NW–SE-trending cross section ( Figure 4d). Overall, the shear zone shows considerable thickness variations ranging from 3.0 seconds (TWT) thick in the footwall of major post-Caledonian fault complexes and in the southwest to 0.5–1.0 second (TWT) thick below the potential spoon-shaped Devonian basin on the western Finnmark Platform in the northeast ( Figure 4a–d; Koehl *et al.*, 2018a). These amount to c. 9 km and 1.5–3 km respectively using an average velocity of 6100 km.s ^-1^ for basement rocks ( Gernigon *et al.*, 2018 their table 1).

At smaller scale, planar moderate-amplitude reflection interpreted as mylonitic fabrics and thrusts alternate with asymmetric, wiggly to oscillating, low- to moderate-amplitude reflections both within the Sørøya–Ingøya shear zone and in adjacent basement rocks ( Figure 5a–d). These reflections can be traced for hundreds of meters to a few kilometers laterally (i.e., discontinuous to semi-continuous; Figure 5a–d). In NE–SW-trending cross section, these asymmetric reflections lean consistently towards the south-southwest ( Figure 5a–c), whereas they lean to the southeast and to the northwest respectively in the southeastern and northeastern part of the western Finnmark in NW–SE-trending cross section ( Figure 5d). These reflections typically show elongated, gently NNE-dipping edges and short, steeply NNE- or SSW-dipping edges resulting in the SSW-leaning geometry ( Figure 5a–c). In places, the long and short edges are parallel to each other with the long, gently dipping edge leaning over the recumbent short edge ( Figure 5a–c). These asymmetric reflections are truncated and offset in a reverse fashion by moderately to gently NNE-dipping, moderate-amplitude disruption surfaces ( Figure 5a–c). The asymmetric reflections are interpreted as SSW-, southeast-, and northwest-verging, in places isoclinal, overturned to recumbent folds in basement rocks and the disruption surfaces as minor top-SSW, top-northwest and top-southeast thrust faults. Other less common geometries include double-verging, box-shaped folds ( Figure 5a) and symmetric folds upright folds ( Figure 5c–d). Notably, upright folds occur mostly in basement rocks underlying and overlying the Sørøya–Ingøya shear zone, particularly within the hinge zone of folded portions of the shear zone ( Figure 5c–d).

Other interesting structures include packages or series of Z- and S-shaped, low- to moderate-amplitude reflections adjacent to and/or stacked onto each other ( Figure 5a–c). These packages are commonly bounded upwards and/or downwards by thrust surfaces. In places, individual S- and Z-shaped reflections are found at the abrupt termination of asymmetric folds against minor thrusts. We therefore propose that S- and Z-shaped reflections represent offset bedding surfaces, and, hence, packages of such reflections to correspond to forward- and backward-dipping duplexes ( Koehl, 2021).

The layering (bedding) and folding of pre-Carboniferous rocks on the western Finnmark Platform and the non-occurrence of the Late Devonian Svalbardian Orogeny in Norway ( Chauvet & Séranne, 1994; Fossen *et al.*, 2013; Osmundsen & Andersen, 1994) and Svalbard ( Koehl *et al.*, 2022b) suggests that they consist of pre-Devonian metasedimentary rocks. Furthermore, the high magnetic character of the rocks on the western Finnmark Platform ( Johansen *et al.*, 1994) suggests that they also partly consist of magmatic intrusions. Potential analogs are Neoproterozoic–lowermost Cambrian metasedimentary rocks of the Barents Sea and Tanafjorden–Varangerbotn regions ( Gorokhov *et al.*, 2001; Siedlecka & Siedlecki, 1967; Siedlecki & Levell, 1978) and of the latest Mesoproterozoic–earliest Neoproterozoic Kalak Nappe Complex ( Roberts, 1998), both of which are intruded by abundant Ediacaran dolerites and Precambrian pegmatites ( Kirkland *et al.*, 2006a; Nasuti *et al.*, 2015a; Ramsay *et al.*, 1979; Rice & Reiz, 1994; Rice *et al.*, 2004; Roberts, 1972; Siedlecka & Roberts, 1992; Figure 1b). Conversely, less magnetic pre-Devonian basement rocks in the Gjesvær Low ( Johansen *et al.*, 1994), which are located within a major Caledonian syncline ( Figure 4d and Figure 5d), likely correspond to post-Cambrian metasedimentary rocks, possibly time equivalents to Silurian metasedimentary rocks of the Magerøy Nappe ( Kirkland *et al.*, 2005; Ramsay & Sturt, 1976), which are only intruded by a few sub-vertical Carboniferous dykes on Magerøya ( Lippard & Prestvik, 1997; Roberts *et al.*, 1991). The dominantly metasedimentary character of the rock successions on the western Finnmark Platform is supported by the partly linear geometry (i.e., layered) of seismic reflections there. The main differences of these reflections with mylonitic thrust surfaces are that they are of lower seismic amplitude (i.e., less acoustic impedance contrast because metasedimentary rocks of more or less the same density, whereas mylonites are denser) and are in places deformed into asymmetric folds (i.e., relatively mild deformation), which is not typical of mylonitic fabrics because it reflects relatively intense deformation.

Above moderate-amplitude reflections of the Sørøya–Ingøya shear zone in the southwest, seismic data show (inverted) triangular packages consisting of poorly continuous to chaotic moderate- to low-amplitude reflections ( Figure 4a and Figure 5a). The poorly continuous reflections still show the same dip and geometries as reflections within the Sørøya–Ingøya shear zone. However, they are separated from underlying asymmetrically folded reflections by southwest-dipping, high-amplitude reflections with subtly undulating to rugose geometries, which are in places truncated by northeast-dipping thrust surfaces of the Sørøya–Ingøya shear zone ( Figure 4a and Figure 5c). The undulating to rugose reflections are interpreted as erosional unconformities later truncated by the Sørøya–Ingøya shear zone. Thus, the less-continuous to chaotic character of the inverted triangular packages indicates that they may represent broken up versions of folded and sheared nappes composing the Sørøya–Ingøya shear zone. The inverted triangular packages are therefore interpreted as syn-orogenic foreland/piggyback deposits ahead of the major thrust ( Figure 4a–c).

***Eastern Finnmark Platform.*** On the eastern Finnmark Platform near the coast of northern Finnmark, seismic data show the occurrence of a gently curving up and down, high-amplitude, intra-basement reflection defining major, 20–25 km wide, NNE–SSW-trending troughs and domes in E–W-trending cross section ( Figure 6a). In places, this high-amplitude reflection is truncated by the Upper Regional Unconformity (which corresponds with the Top-basement reflection in the area) and by post-Caledonian normal faults ( Figure 6a). We therefore interpreted the 20–25 km wide troughs and domes as major Caledonian synclines and anticlines. In E–W-trending cross section, upright fold geometries are as common as asymmetric folds and are typically found within the core of the 20–25 km wide folds. Note that previous works (e.g., Bugge *et al.*, 1995) interpreted the western flank of the eastern major anticline in Figure 6a as a Carboniferous normal fault. However, the apparent disruption of seismic reflections there is clearly related to seismic artifacts, including multiples and diffraction rays (see Figure 6a and Koehl *et al.*, 2018a their figure 6d).

The packages of seismic reflections above the folded high-amplitude seismic reflection defining the main Caledonian folds show more continuous and more mildly folded, moderate-amplitude seismic reflections that are truncated by the Upper Regional Unconformity upwards ( Figure 6a). Despite the less disrupted and more continuous character of these reflections, their folded character, locally including east- and west-verging, partly overturned folds ( Figure 6a), suggests that they are pre-Devonian in age. A possibility is that they belong to the well-layered, low-grade metasedimentary rocks of the Magerøy Nappe, which crop out to the south onshore Magerøya ( Figure 1b).

Other features of interest include asymmetric, U-shaped, high-amplitude reflections disrupting intra-basement bedding surfaces ( Figure 6a). The disruptive character of these reflections together with the asymmetric U-shaped geometry suggests that they represent saucer-shaped sills. The truncation of folded basement bedding surfaces by the sills suggests that they are post-Caledonian, and, therefore, may represent offshore equivalents to the Mississippian dolerite dykes onshore Magerøya ( Lippard & Prestvik, 1997; Roberts *et al.*, 1991).

Farther northeast in NNE–SSW-trending cross section, seismic data show a major, NNE-dipping, kilometer-thick package of moderate- to low-amplitude seismic reflections including asymmetric, SSW-leaning, low-amplitude reflections with poor lateral continuity separated by moderate-amplitude, planar, continuous reflections ( Figure 6b) similar to those on the western Finnmark Platform ( Figure 5a–c). Upwards, the NNE-dipping reflection package is truncated by the mid-Carboniferous unconformity, which corresponds with the Top-basement reflection in the area. In addition, this reflection package is offset by a Devonian–Carboniferous normal fault that dies out upwards below the Base Triassic unconformity and merges downwards with planar, continuous reflections within the NNE-dipping reflection package ( Figure 6b). The NNE-dipping reflection package is therefore interpreted as a major top-SSW ductile thrust consisting of SSW-verging folds and top-SSW mylonitic thrust surfaces. In E–W-trending cross section, this kilometer-thick thrust is folded into 15–35 km wide, NNE–SSW- to N–S-striking folds. The interaction of these NNE–SSW- to N–S-striking folds and the north-northeastern dip of the thrust results into NNE- to north-plunging fold axes in map view ( Figure 7). In the south and southwest, these folds correlate with onshore folds mapped during field studies on the Varanger Peninsula ( Roberts, 1996; Siedlecki, 1980), on the Nordkinn Peninsula ( Roberts, 1998; Roberts, 2008a; Roberts, 2008b; Roberts & Williams, 2013), and on Magerøy ( Andersen, 1981; Robins, 1990a; Robins, 1990b; Figure 7 and Figure 8).

### Bathymetric–topographic data

In the northeastern portion of the Kalak Nappe Complex, bathymetric and topographic data reveal sets of high-frequency, linear, smooth, gently dipping escarpments at rugose outcrops, which trend dominantly NNE–SSW ( Figure 9a–b), i.e., oblique to N–S-trending glacial lineations in the area ( Koehl *et al.*, 2019; Ottesen *et al.*, 2008). In the Nordkinn Peninsula, Sværholt Peninsula, Magerøya, and the Porsanger Peninsula, these escarpments can be tied with 5–15 kilometers wide, NNE–SSW-trending, partly overturned to recumbent Caledonian folds ( Figure 8; Geul, 1958 in Andersen, 1981; Krill *et al.*, 1988; Ramsay *et al.*, 1985; Roberts, 1987; Roberts, 1998; Roberts & Williams, 2013), which are also supported by the attitude of magnetic anomalies in the area ( Figure 10 and Figure 12). Typically, ductile fabrics on bathymetric and topographic data show rounded to oval geometries (e.g., at fold hinges), and gently to steeply dipping attitudes matching that the local folded foliation and bedding surfaces (e.g., Figure 9a–d). Note that the continuation of the western syncline on Magerøya to the Stikonjargga Peninsula in the south ( Figure 1b) is based on the correlation of rocks of the Hellefjord Group ( Ramsay *et al.*, 1985) to the Magerøy Nappe ( Kirkland *et al.*, 2006b; Gasser *et al.*, 2015; Figure 2). This correlation is further supported by the presence of a SSW-narrowing, NNE–SSW-trending, negative magnetic anomaly extending from Magerøya to the Stikonjargga Peninsula ( Figure 1b; Figure 11a).

Bathymetric data in northwestern Finnmark show major bends in the strike of the ductile fabrics from NNE–SSW- to WNW–ESE ( Figure 8), which are supported by onshore field mapping and magnetic anomalies. A concrete example is the gradual bend from a NNE–SSW strike in Magerøya where metasedimentary rocks of the Magerøy Nappe are deformed into tight NNE–SSW-striking folds ( Andersen, 1981; Robins, 1990a; Robins, 1990b) to ENE–WSW in rocks of the Kalak Nappe Complex in western Magerøya and WNW–ESE in Hjelmsøya and Rolvsøya (locations in Figure 1b) where overturned fold structures in the field strike WNW–ESE, and back to a NNE–SSW strike south and west of Rolvsøya ( Ramsay *et al.*, 1979; Roberts, 1981). There, glacial lineations trend WNW–ESE and ENE–WSW and are largely oblique to the ductile fabrics ( Figure 8; Koehl *et al.*, 2019; Ottesen *et al.*, 2008).

Comparable bends of dominantly NNE–SSW-striking ductile fabrics are also observed onshore the northeastern part of the Porsanger Peninsula ( Ramsay *et al.*, 1985; Roberts, 1998), Sørøya ( Appleyard, 1965; Krill *et al.*, 1988; Roberts, 1973; Speedyman, 1983; Figure 8), and the Repparfjord–Komagfjord tectonic window ( Pharaoh *et al.*, 1982; Pharaoh *et al.*, 1983; Reitan, 1963; Torgersen *et al.*, 2015; Figure 2). On the northeastern part of Porsanger Peninsula, gently northwest-dipping escarpments show undulating geometries alternating between NNE–SSW and ENE–WSW strikes. These correlate in the field with the southeastern flank of a major, open, upright fold with the hinge zone in the central part of the Porsanger Peninsula ( Gayer *et al.*, 1987; Ramsay *et al.*, 1985). These contrast with glacial lineations, which trend NNE–SSW-trending and WNW–ESE respectively in Porsangerfjorden and on the Porsanger Peninsula ( Figure 8; Koehl *et al.*, 2019; Ottesen *et al.*, 2008).

Onshore and nearshore Sørøya notably, gently dipping escarpments bend from a NNW–SSE strike in northeastern Sørøya to an ENE–WSW strike in central Sørøya and a N–S to NNE–SSW strike in the south ( Figure 9e). These variations coincide with the attitude of major, tens of kilometers wide, steeply plunging fold structures in the field ( Appleyard, 1965; Krill & Zwaan, 1987; Roberts, 1973; Speedyman, 1983) and are in places sub-orthogonal to glacial lineations ( Figure 8).

Yet another example is the bending of gently northwest-dipping escarpments in the Repparfjord–Komagfjord tectonic window and between Rolvsøya and the Porsanger Peninsula (locations in Figure 1b) from a NNE–SSW strike in the southwest to a WNW–ESE strike in the northeast ( Figure 9c–d). In the Repparfjord–Komagfjord tectonic window notably, previous studies mapped the occurrence of a several kilometers wide, gently northeast-plunging fold within Paleoproterozoic basement rocks showing a geometry comparable to that of the described escarpments ( Pharaoh *et al.*, 1982; Pharaoh *et al.*, 1983; Reitan, 1963; Torgersen *et al.*, 2015), which therefore likely reflect the attitude of ductile fabrics.

### Magnetic data

Regional aeromagnetic data in Finnmark and the Barents Sea were described and interpreted in various works ( Gernigon & Brönner, 2012; Gernigon *et al.*, 2014; Henderson *et al.*, 2015; Koehl *et al.*, 2019; Nasuti *et al.*, 2015a; Nasuti *et al.*, 2015b). The present section focuses on anomalies that delineate structures (e.g., folds and faults) in basement rocks and Caledonian nappes. Fold structures will be described from the west to the east.

***Folds.*** Overall, the tilt-derivative magnetic data show triangular- (where partly imaged) to wedge-shaped (where completely imaged) magnetic anomalies interpreted as gently NNE-plunging folds onshore northern Finnmark bending into NNW-plunging geometries northward onto the eastern Finnmark Platform ( Figure 14). The three-dimensional geometry of the folds likely results from top-SSW Timanian contraction in the latest Neoproterozoic and superimposed top-southeast Caledonian contraction in the Ordovician–Silurian, which is partly shown in Gabrielsen *et al.* (2022).

Metasedimentary rocks of the Kalak Nappe Complex in northern Sørøya consist of quartzite interbedded with marble, pelite and graphite schist (Falkenes and Åfjord groups) and garnet-rich micaschist (Storelv Group; Figure 2). In the field, these units are deformed into subhorizontal, bending, Caledonian, NNE–SSW- to ENE–WSW-striking folds. In tilt-derivative magnetic data, the marble, pelite, graphite schist and micaschist units correlate with high-positive anomalies ( Figure 13a). In map view, the bending of these Caledonian folds defines steep folds with NW–SE-striking axial planes.

Arcuate and (right-) curving geometries of dominantly NNE–SSW-striking magnetic anomalies between Rolvsøya and the Porsanger Peninsula mimic the geometry of anomalies in the Repparfjord–Komagfjord tectonic window associated with a gently northeast-plunging fold ( Koehl *et al.*, 2019; Pharaoh *et al.*, 1982; Pharaoh *et al.*, 1983; Torgersen *et al.*, 2015 their Figure 10e). The presence of a northeast-plunging fold is supported by bedding attitudes in southern Rolvsøya (north-dipping) and in Reinøykalven and Bjørnøya (southeast-dipping; Roberts, 1998; locations in Figure 1b), and by correlation with nearshore escarpments reflecting ductile fabrics ( Figure 8 and Figure 9d).

In the central part of the Porsanger Peninsula, a prominent 5–10 kilometers wide, NNE–SSW-trending, positive magnetic anomaly aligns with outcrops of the Repparfjord–Komagfjord tectonic window farther south, which are deformed into a 5–10 kilometers wide anticline that is also visible on magnetic data ( Koehl *et al.*, 2019; Pharaoh *et al.*, 1982; Pharaoh *et al.*, 1983). It is therefore possible that the two anomalies reflect related, NNE–SSW-striking anticlines.

The syncline observed on seismic data just north of Magerøya coincides with a magnetic low of comparable width ( Gernigon *et al.*, 2014; Figure 14) that extends from offshore areas to onshore areas of Magerøya where rocks of the Magerøy Nappe crop out in a c. 20–25 kilometers wide syncline. Thus, the synform on seismic data north of Magerøy is interpreted as the northeastward continuation of this syncline with rocks of the Magerøy Nappe in the core. It is also possible that the negative triangular magnetic anomaly represents Carboniferous collapse basins on the Finnmark Platform ( Koehl *et al.*, 2018a). However, the continuation of this negative anomaly below the island of Magerøy, where post-Caledonian sedimentary deposits are absent, suggests that the anomaly is at least partly due to the presence of the Magerøy Nappe in a wedge-shaped, NNE–SSW-striking Caledonian syncline. This interpretation is supported by the presence of NNE–SSW-striking folds in northeastern Magerøya in the field ( Andersen, 1981, e.g., his Figure 6), which also show as narrow magnetic anomalies on the onshore–nearshore tilt-derivative magnetic map ( Figure 11a).

The 5–8 km wide anticline at the mouth of Porsangerfjorden was mapped as a right-bending, dominantly positive anomaly on onshore–nearshore tilt-derivative magnetic data ( Figure 11b). In map view, the fold seems to affect 4–5 km wide, 10–20 km long positive magnetic anomalies, which bend mildly to the southwest and coincide with onshore occurrences of folded Neoproterozoic intrusions in the northeastern part of the Porsanger Peninsula ( Nystuen, 2013; Waltham, 2003).

Farther east, 7–12 km wide, NE–SW-striking, gently northeast-plunging syncline and anticline were mapped in the field on the Sværholt Peninsula ( Roberts, 1987; Roberts, 1998), and a similar, 3–4 km wide syncline in Vindhamran in the western part of the Nordkinn Peninsula ( Roberts, 1998; Roberts & Siedlecka, 2013; Roberts & Williams, 2013). The contours of these folds are delineated by narrow, oval-shaped, high positive anomalies on onshore–nearshore tilt-derivative magnetic data ( Figure 11b), which are explained by the presence of highly magnetic minerals (magnetite and titanite) in metasedimentary beds of the Kalak Nappe Complex ( Roberts, 2007; Roberts & Williams, 2013; Figure 11c).

Offshore magnetic and tilt-derivative data just north of the Nordkinn Peninsula on the eastern Finnmark Platform shows the presence of a major, 25–30 km wide, triangular, positive anomaly ( Figure 14 and Figure 15). Southwards, the triangular shape of the anomaly widens onshore the central and eastern parts of the Nordkinn Peninsula and continues as two positive magnetic anomalies ( Figure 11c and Figure 13b), one of which is correlated to metasedimentary beds enriched in highly magnetic minerals in Vindhamran ( Roberts, 2007). The central and eastern parts of the Nordkinn Peninsula are therefore believed to be deformed into a major, ≥ 30 km wide, NNE–SSW-trending anticline. The metasedimentary successions in the area include notably a c. 3 km thick succession of arkosic metasandstone overlain by a 3–5 km thick phyllite-dominated metasedimentary succession ( Roberts & Siedlecka, 2013; Roberts & Williams, 2013), itself overlain by the metasedimentary beds enriched in highly magnetic minerals of Roberts (2007). These successions are apparently repeated symmetrically across a NNE–SSW-trending axis running near the center of the peninsula as supported by the occurrence of a narrow, NNE–SSW-trending, positive magnetic anomaly in the easternmost part of the peninsula similar to that associated to the metasedimentary beds with highly magnetic minerals in the western part of the peninsula ( Figure 11c). Bedding surfaces in metasedimentary rocks both in the western and eastern limb of the major anticline dip moderately to steeply towards the west-northwest ( Roberts & Siedlecka, 2013; Roberts & Williams, 2013), thus suggesting that the eastern limb is overturned and giving the fold a south-southeast vergence.

In the northwesternmost part of the Varanger Peninsula, the outcrops of the Berlevåg Formation coincide with a 25 km wide, wedge-shaped, curving, NNE–SSW-elongated, dominantly positive anomaly on tilt-derivative magnetic data ( Figure 14 and Figure 13c). This anomaly extends well into Tanafjorden in the west (just east of the coastline of the Nordkinn Peninsula; location in Figure 1b) and onto the eastern Finnmark Platform to the north, where it broadens up to 50 km and bends into a NNW–SSE trend, but pinches out rapidly to the south/southwest. Since it is adjacent to the major anticline in the central and eastern part of the Nordkinn Peninsula, this magnetic anomaly is interpreted as a major wedge-shaped, NNE–SSW-striking (trough-shaped) syncline. This synclinal geometry of the Berlevåg Formation is confirmed by field studies and geological mapping in the area showing that the eastern limb of the fold involves northwest-dipping bedding surfaces ( Siedlecka & Siedlecki, 1967; Siedlecki, 1980). Farther southwest on the Digermulen Peninsula, field mapping by Siedlecki (1980) and Siedlecka *et al.* (2006) reveals the presence of an analogous, at least 15 km wide, wedge-shaped, NE–SW-striking syncline extending from the western shore of Langfjorden to Tanafjorden ( Figure 1b and Figure 13c). Due to their occurrence in the footwall of the Caledonian thrust at the base of the Kalak Nappe Complex and in a similar structure setting (wedge-shaped syncline of comparable dimensions), we correlate the Berlevåg Formation to the Ediacaran–Lower Ordovician ( Ebbestad *et al.*, 2018; Högström *et al.*, 2017; Jensen *et al.*, 2017; Siedlecki, 1980) Digermulen Group (Laksefjord Nappe Complex), i.e., in agreement with previous correlations (e.g., Corfu *et al.*, 2014; Kirkland *et al.*, 2008b; Figure 2). An Ediacaran–Lower Ordovician age for the Berlevåg Formation is in agreement with its position in a syncline, i.e., structurally (although the Kalak Nappe Complex is partly thrust over the Berlevåg Formation syncline) and stratigraphically above Tonian to lower Cryogenian rocks of the Barents Sea Group and of the upper Cryogenian Løkvikfjellet Group ( Roberts & Siedlecka, 2012; Siedlecki & Levell, 1978) and latest Mesoproterozoic–Tonian metasedimentary rocks of the Kalak Nappe Complex ( Kirkland *et al.*, 2006a; Kirkland *et al.*, 2007a; Figure 2). Nevertheless, since the Berlevåg Formation was affected by a hydrothermal event at 555 ± 15 Ma ( Kirkland *et al.*, 2008b), it is probable that it is mostly Ediacaran in age ( Figure 2).

In the northwestern part of the Varanger Peninsula, magnetic data show 1–4 km wide, NE–SW-trending, spike-shaped positive anomalies in Kongsfjorden ( Figure 13c). Since the bedrock there consists of Tonian to lower Cryogenian metasedimentary rocks of the Barents Sea Group (Båsnæring and Kongsfjord formations; Siedlecka & Siedlecki, 1967; Siedlecki, 1980; Figure 2) flanked in the northwest and southeast by younger metasedimentary strata of the upper Cryogenian Løkvikfjellet Group ( Roberts & Siedlecka, 2012; Siedlecki & Levell, 1978; Figure 1b) dipping respectively to the northwest between Sandfjord and Berlevåg and to the southeast in Syltefjordfjellet ( Siedlecka & Siedlecki, 1967; Siedlecki, 1980), the area is therefore believed to be deformed into a 15–20 km wide, NE–SW-striking anticline with the fold axis running within inner Kongsfjorden. The northeastwards narrowing of the spike-shaped magnetic anomaly suggests a northeast-plunging geometry for the anticline ( Figure 13c).

In Syltefjordfjellet, both onshore and offshore magnetic data show the occurrence of a c. 15 km wide, rhomboidal to oval positive anomaly ( Figure 14 and Figure 11d and Figure 13c; Koehl, 2024). In the southwest, the anomaly pinches out near the bottom of Båtsfjorden, whereas in the northeast it proceeds into the Russian Barents Sea ( Figure 14 and Figure 11d and Figure 13c). Since the positive magnetic anomaly in Syltefjordfjellet coincides with the occurrence of relatively younger, gently dipping rocks of the Løkvikfjellet Group, which are flanked to the west and southeast by relatively older rocks of the Barents Sea Group ( Siedlecka & Siedlecki, 1967; Siedlecki, 1980; Figure 1b), we interpret the anomaly to reflect the presence of a 20–25 km wide, NE–SW-striking syncline. Noteworthy, Siedlecki (1980) mapped two synclines centered (with fold axis running) along two NE–SW-elongated strips of outcrops of the Båtsfjord Formation. However, relatively younger rocks of the Løkvikfjellet Group crop out in between these two strips and, therefore, suggest the presence of a major, 20–25 km wide syncline ( Figure 1b). This is further supported by the presence of older rocks of the Båsnæring Formation in Båsnæringsfjellet in the northwest and on the southeastern shore of Syltefjorden in the southeast ( Siedlecka & Siedlecki, 1967; Siedlecki, 1980; location in Figure 1b). Hence, the two smaller (5–10 km wide) synclines mapped by Siedlecki (1980) may represent parasitic folds of the main 20–25 km wide syncline.

In the eastern part of the Varanger Peninsula, tilt-derivative magnetic data show a 45–65 km wide, ENE–WSW-trending negative anomaly extending from Syltefjorden to tens of kilometers east of the Varanger Peninsula ( Figure 14). Within this broad negative anomaly, onshore tilt-derivative magnetic data show narrow, hundreds of meters wide, curving positive magnetic anomalies with NNE-pointing spike shapes ( Figure 13c). These narrow anomalies coincide with and display similar geometries to 3–5 km wide, NNE-plunging synclines in the area ( Siedlecki, 1980), thus suggesting that they reflect north- to northeast-dipping bedding. Despite these 3–5 km wide synclines, the dominance of rocks of the Barents Sea Group in the area (older than rocks of the Løkvikfjellet Group) suggest that the eastern part of the Varanger Peninsula consist of a tens of kilometers wide anticline, which is supported by recent field studies (e.g., Gabrielsen *et al.*, 2022).

***Faults.*** Magnetic data in northern Finnmark also show prominent WNW–ESE-striking positive anomalies interpreted as Carboniferous dolerite dykes intruded along brittle splays following the trace of the Trollfjorden–Komagelva Fault Zone ( Koehl *et al.*, 2019; Nasuti *et al.*, 2015a). In places, these dykes show up to 2–3 km wide left bends ( Figure 11a) mimicking the local ductile fabrics attitude, e.g., in western Magerøya where it bends from a northeasterly strike in the east to an E–W strike in the west ( Robins, 1990a). Other occurrences are seen on the Sværholt Peninsula and in Porsangerfjorden ( Figure 11b).

The 45–65 km wide negative magnetic anomaly associated with the major anticline in the easternmost part of the Varanger Peninsula is transected by narrow, hundreds of meters wide, linear, ENE–WSW-trending positive anomalies correlated with Late Devonian dykes ( Nasuti *et al.*, 2015a; Roberts, 2011). Based on previous field mapping of ENE–WSW-striking Caledonian thrusts in the northeastern part of the Varanger Peninsula ( Gabrielsen *et al.*, 2022; Siedlecki, 1980), it is probable that the Devonian dykes in the area intruded along such structures as they seem to follow the Caledonian structural trend. This is supported by the reactivation of NNW- to northwest-dipping Caledonian thrusts on the Varanger Peninsula as post-Caledonian brittle normal faults ( Siedlecka & Roberts, 2012). The geometry of these dykes contrasts markedly with that of linear N–S-striking Devonian dykes intruded along right-stepping brittle fractures in the southeastern part of the Varanger Peninsula, which probably correspond to post-Caledonian extensional faults ( Guise & Roberts, 2002; Herrevold *et al.*, 2009; Nasuti *et al.*, 2015a).

## Discussion

The discussion focuses first on the timing of formation of the identified two major NNE-dipping thrust systems on the Finnmark Platform and on a possible correlation with onshore structures. Then, we review the implications of the present work for major tectono-magmatic events in northern Norway, including the intrusion of the Seiland Igneous Province, the deposition and thrusting of the Kalak Nappe Complex, the rifting and width of the Iapetus Ocean, the Porsanger Orogeny, and the Finnmarkian event. Finally, we summarize the findings of the present study into a model for the geological evolution of northern Norway from the Grenvillian–Sveconorwegian Orogeny in the latest Mesoproterozoic to the Caledonian Orogeny in the early–mid Paleozoic (i.e., from ca. 1050 to 420 Ma).

### Timing of formation of the top-SSW thrust systems on the Finnmark Platform

Seismic data on the Finnmark Platform show the occurrence of two major, overall NNE-dipping thrust systems, which are folded into 15–35 km wide, NNE-plunging folds and show evidence of top-SSW movement ( Figure 4a–c, Figure 6a, and Figure 7). The vergence of the major thrust systems and associated numerous minor structures, such as asymmetric folds and thrusts, strongly suggests that the two thrust systems formed during the Timanian Orogeny ( Herrevold *et al.*, 2009; Kostyuchenko *et al.*, 2006; Olovyanishnikov *et al.*, 2000; Siedlecka, 1975).

This is supported by the apparent reworking of the southern thrust system on the western Finnmark Platform previously interpreted as the Sørøya–Ingøya shear zone ( Koehl *et al.*, 2018a), where it is deformed into major, NE–SW-striking folds and shows indications for top-southeast movements in the southeast and top-northwest movements in the northwest ( Figure 4d), i.e., most likely subsequently to the episode top-SSW tectonism. A suitable event to have partially reworked a preexisting NNE-dipping thrust system into NE–SW-striking folds and to have generated top-southeast and top-northwest movements on the folded portions of the thrust is the Caledonian Orogeny ( Gayer *et al.*, 1985; Gayer *et al.*, 1987; Townsend *et al.*, 1986; Townsend, 1987). Our interpretation is in agreement with Gernigon *et al.* (2014) who interpreted the basement ridge in the footwall of the Troms–Finnmark Fault Complex on the Finnmark Spur as Caledonian back-thrusts, thus further supporting a Caledonian reworking of the top-SSW thrust system.

The (double-) folding patterns observed in metasedimentary rocks affected by the Timanian Orogeny on the Varanger Peninsula (e.g., dome-shaped Ragnarokk Anticline; Gabrielsen *et al.*, 2022; Roberts, 1996; Siedlecka & Siedlecki, 1971; Figure 1a) is similar to what was inferred for NNE-dipping Timanian thrust systems in Svalbard and the Barents Sea ( Koehl, 2020; Koehl *et al.*, 2022a; Koehl *et al.*, 2023). In addition, the NNE-plunging folds identified on magnetic data onshore–nearshore northern Finnmark ( Figure 14 and Figure 13c) correlate well with the NNE-plunging folding of the northern thrust system ( Figure 7 and Figure 8).

Notably, Koehl *et al.* (2018a) interpreted the significant thickness variations of the southern thrust system to arise from Devonian–Carboniferous core complex exhumation along inverted portions of the shear zone. Though this interpretation may still be partly valid, it does not explain the opposite vergence of contractional structures within the thrust system (top-northwest in the northwest and top-southeast in the southeast).

### The western continuation of the Trollfjorden–Komagelva Fault Zone offshore

A particularity of the southern thrust system is that it bends into a WNW–ESE strike in the northeast and can be traced up to c. 30 km off the Sværholt Peninsula and the Nordkinn Peninsula, near the possible western continuation of the Timanian thrust front, the Trollfjorden–Komagelva Fault Zone ( Gabrielsen, 1984; Gabrielsen & Færseth, 1989; Koehl *et al.*, 2018a; Koehl *et al.*, 2019; Lea, 2016; Roberts *et al.*, 2011). Based on previous studies in northwestern Russia, the Timanian Orogeny was associated with southwest-directed subduction as suggested by eclogite and blueschist metamorphism and related intrusions ( Beckholmen & Glodny, 2004; Dovzhikova *et al.*, 2004; Gee *et al.*, 2000; Glodny *et al.*, 2004; Kuznetsov *et al.*, 2007; Pease *et al.*, 2004; Remizov & Pease, 2004; Remizov, 2006). The sigmoidal geometry of structures in the southern thrust system (e.g., Figure 4a–c and Figure 5a–c) are comparable in shape and size to subduction-related complexes in Norway and the UK ( Freeman *et al.*, 1988; Péron-Pinvidic & Osmundsen, 2020). In addition, the observed bend of the southern thrust system on the western Finnmark Platform is comparable in size to that of major plate-boundary faults such as the San Andreas fault in southern California (e.g., Crowell, 1979; Janecke *et al.*, 2018; Koehl *et al.*, 2022c; Koehl *et al.*, 2022d). Considering the suitable location of the westernmost trace of the southern thrust system near the continuation of the Trollfjorden–Komagelva Fault Zone, we propose that it represents the western continuation of the Trollfjorden–Komagelva Fault Zone offshore rather than the commonly proposed WNW–ESE-striking brittle segment of the Troms–Finnmark Fault Complex ( Gabrielsen, 1984; Gabrielsen & Færseth, 1989; Lea, 2016; Roberts *et al.*, 2011). This correlation and partial reworking of the offshore shear zone into a top-southeast thrust during the Caledonian Orogeny on the western Finnmark Platform explains the observed major bend of ductile fabrics in the Kalak Nappe Complex from WNW–ESE in western Magerøya and Hjelmsøya to NE–SW in Ingøya and Rolvsøya in the field ( Roberts, 1981; Robins, 1990a) and on bathymetric data ( Figure 8). In this case, truncation of WNW–ESE-striking Timanian fabrics by a top-southeast Caledonian shear zone in the western Finnmark Platform (e.g., Koehl *et al.*, 2018a) is not required.

Regarding the fault-propagation tip model proposed by Koehl *et al.* (2019), this model focused on shallow brittle overprints of the Trollfjorden–Komagelva Fault Zone along which (Devonian?–) Carboniferous dykes were intruded ( Lippard & Prestvik, 1997; Roberts *et al.*, 1991). However, although they may involve shallow brittle segments, orogenic structures such as thrust fronts are generally dominantly ductile at depth. Thus, even if the shallow and younger (Carboniferous) brittle overprints of the Trollfjorden–Komagelva Fault Zone seem to die out westwards around the island of Magerøya ( Koehl *et al.*, 2019), these are distinct from the ductile portion of the Trollfjorden–Komagelva Fault Zone at depth.

Alternatively, the folded southern Timanian thrust offshore may represent the western continuation of another presumed Timanian thrust just off the northern coast of Finnmark ( Figure 1b and Figure 7). This is supported by the large proportion of graywacke in metasedimentary rocks of the Barents Sea Group ( Siedlecka, 1975; Siedlecki, 1980), which are typical of active continental margins and accretionary prisms (e.g., Dickinson, 1970), by the gradual transition of the Barents Sea Group with more fluvial rocks of the Tanafjorden–Varangerfjordbotn Group ( Rice, 1994) on the Varanger Peninsula, and by the interbedded character of the two groups at the speculated location of the Trollfjorden–Komagelva Fault Zone in the western part of the Varanger Peninsula ( Rice, 1994; Figure 2), which all suggest that the actual Timanian thrust front lies just north of the coast of Finnmark instead of onshore the Varanger Peninsula ( Figure 1b and Figure 7). This would imply that the Timanian subduction trench and suture are located below or north of the Varanger Peninsula depending on their vergence, which is possibly dipping to the south ( Pease *et al.*, 2004).

Regardless of the correlation with the Trollfjorden–Komagelva Fault Zone, it follows that other brittle–ductile structures in Finnmark, which yielded late Neoproterozoic–earliest Cambrian ages but strike NE–SW at present, may represent inherited Timanian thrusts, which were reworked during the Caledonian Orogeny. Notably, Torgersen *et al.* (2014) obtained 531.1 ± 11.1 and 654.8 ± 13.4 Ma K–Ar ages for synkinematic illite–muscovite along the Kvenklubben Fault, which they interpreted as a tectono-thermal event. The present study suggests that these ages may reflect early and late Timanian thrusting respectively.

In western Troms, U–Pb geochronological analyses of intensely deformed basement rocks in the West Troms Basement Complex yielded four 650–550 Ma ages that correspond with the time frame of the Timanian Orogeny ( Bergh *et al.*, 2015). Notably, the 643 Ma intercept they obtained for a pegmatite dyke in garnet–mica schist with marble lenses in Baltsfjorden, Senja, was interpreted as secondary lead loss in the Neoproterozoic because it did not show correspondence with any geological events known to have affected rocks of the West Troms Basement Complex ( Figure 2). However, considering the proximity of the Timanian front on the western Finnmark Platform (< 100 kilometers instead of > 250 kilometers; Figure 1b and Figure 4a–d) and related magmatic complex (Seiland Igneous Province; < 50 kilometers), it is conceivable that Timanian deformation and related magmatism (at least mildly) affected rocks in western Troms, thus leading to limited reactivation and/or overprinting and intrusions in adjacent basement rocks.

### Implications for folding in Finnmark

Bathymetric–topographic and magnetic data in Finnmark confirm the presence of NNE–SSW-striking folds in the northeastern portion of the Kalak Nappe Complex in the Nordkinn Peninsula, Laksefjorden, Sværholt Peninsula, Magerøya, Porsangerfjorden, and the Porsanger Peninsula, of gently-NNE-plunging folds in the Repparfjord–Komagfjord tectonic window and between the Porsanger Peninsula and Rolvsøya (locations in Figure 1b), and of overturned, WNW–ESE- to E–W-striking, moderately dipping to sub-vertical folds in the west on Hjelmsøya, the northwesternmost Porsanger Peninsula, Rolvsøya, and Sørøya ( Figure 8– Figure 12).

More specifically, NNE–SSW-striking anticlines in the northeastern Kalak Nappe Complex broaden where adjacent synclines become narrower or die out ( Figure 8). These width fluctuations notably occur along a WNW–ESE-trending axis extending from the Stikonjargga Peninsula to the Sværholt Peninsula (location in Figure 1b) and suggest the presence of WNW–ESE-striking folds in the eastern part of the Kalak Nappe Complex too, albeit with upright geometries ( Figure 8). Similarly, the gently-NNE-plunging anticline in the Repparfjord–Komagfjord tectonic window and in the central part of the Porsanger Peninsula appear to narrow and die out respectively to the northeast and the southwest ( Figure 8– Figure 12). The alignment of these two anticlines, their similar strike and width (5–10 km) suggest that they may correspond to the same structure, which appears mildly deformed into broad, WNW–ESE-striking folds ( Figure 8– Figure 12).

The change in attitude of WNW–ESE-striking folds from sub-horizontal in the east (eastern Porsanger Peninsula, Magerøya, Nordkinn Peninsula, Sværholt Peninsula, Stikonjargga Peninsula in Rykkelid, 1992; locations in Figure 1b) to moderately (western Magerøya) and steeply east-plunging (Hjelmsøya, western Porsanger), and to sub-vertical in the west (Rolvsøya–Ingøya, Sørøya) is proposed to be related to top-southeast Caledonian thrusting (and related folding) along the Sørøya–Ingøya shear zone segment of the Trollfjorden–Komagelva Fault Zone, which is located ca. 15–20 kilometers northwest of the coast of Rolvsøya, Ingøya, and Sørøya.

### Implications for the Seiland Igneous Province

Recent studies show that the Seiland Igneous Province likely reaches at least 9 km deep into the crust ( Larsen *et al.*, 2018; Pastore *et al.*, 2016). By contrast, thickness estimates for the Kalak Nappe Complex in the area are in the range of ≤ 7.5 km based on structural cross sections ( Gayer *et al.*, 1985; Ramsay *et al.*, 1985) and ≤ 1.7 km based on new high-resolution magnetic data inversion ( Nasuti & Roberts, 2023). This indicates that the Seiland Igneous Province reaches well into Baltican Archean–Proterozoic basement rocks at depth, onto which rocks of the Kalak Nappe Complex were thrust ( Figure 2). This is supported by the interpretation of magnetic data showing that Baltican basement rocks extend into western Troms (West Troms Basement Complex; Bergh *et al.*, 2010; Zwaan, 1995) and offshore onto the western Finnmark Platform as major NNW–SSE-striking folds ( Koehl *et al.*, 2019; their Figure 7a and paragraph 2 pp. 15). Together with the proximity (< 30 km) of the Timanian front thrust (Trollfjorden–Komagelva Fault Zone; Figure 1a–b and Figure 4a–d), this suggests that the Seiland Igneous Province was intruded on the Baltican margin of the Iapetus Ocean.

Most previous studies relate the 580–560 Ma and 530–520 Ma magmatism of the Seiland Igneous Province to rifting ( Bergström & Gee, 1985; Elvevold *et al.*, 1994; Krill & Zwaan, 1987; Kirkland *et al.*, 2008b; Larsen *et al.*, 2018; Roberts *et al.*, 2006; Roberts *et al.*, 2010), although backarc and collisional settings are equally possible based on current evidence ( Roberts *et al.*, 2006; Roberts *et al.*, 2010; Robins & Gardner, 1975). A continental backarc setting (e.g., Draut & Clift, 2001; Vasey *et al.*, 2021) is supported by calc-alkaline rocks in the Seiland Igneous Province ( Roberts *et al.*, 2006; Robins & Gardner, 1975; Speedyman, 1983; Stumpfl & Sturt, 1964; Sturt & Ramsay, 1965) and by the proximity of the Seiland Igneous Province with the Timanian thrust front in northern Norway (Trollfjorden–Komagelva Fault Zone; Siedlecka & Siedlecki, 1967; Siedlecka, 1975) and on the western Finnmark Platform (≤ 30 km; Figure 1a–b and Figure 4a–d). In addition, contemporaneous ages were obtained for collision- to subduction-related calc-alkaline and late–post-orogenic alkaline magmatic suites in Russia respectively dated to 700–515 Ma and 565–500 Ma ( Kuznetsov *et al.*, 2007). The former intruded in a continental-arc (to syn-collisional) setting with a southwest-dipping subduction zone (i.e., beneath Baltica) just north of the Timanian thrust front in Russia (Timan Range, Urals, and under the Pechora Basin; Dovzhikova *et al.*, 2004; Gee *et al.*, 2000; Kuznetsov *et al.*, 2007; Pease *et al.*, 2004), whereas the elongate shapes of the latter were interpreted to reflect the onset of late–post-orogenic extension ( Kuznetsov *et al.*, 2007; Larionov *et al.*, 2004). The composition of felsic to ultramafic rocks of the Seiland Igneous Province (e.g., Larsen *et al.*, 2018) does compare well with that of late–post-Timanian alkaline suites in northwestern Russia ( Larionov *et al.*, 2004). It is therefore proposed that the Seiland Igneous Province is part of a regional, extensional–transtensional igneous suite extending from the Russian Timanides to northern Norway and the Norwegian Barents Sea (foliated gabbro/basalt in well 7120/1-1). This implies that the 555 ± 15 Ma hydrothermal event in the Berlevåg Formation ( Kirkland *et al.*, 2008b) and the 577 ± 14 Ma metadolerite dykes on Varangerhalvøya ( Rice *et al.*, 2004; Figure 2) are also related to the Timanian Orogeny. A backarc setting was previously suggested for the Seiland Igneous Province and the 577 ± 14 Ma metadolerite dykes based on petrochemical analyses (Alexander Hugh Rice pers. comm., 2022; Rice *et al.*, 2004; Roberts, 1975; Robins & Gardner, 1975; Speedyman, 1983). Furthermore, thermochemical and dynamic modelling by Griffin *et al.* (2013) suggests that the Seiland Igneous Province is part of the last phase of melting of a rapidly ascending diapir of subducted oceanic lithosphere. Interestingly, the first calc-alkaline intrusions of the Seiland Igneous Province are coeval with the onset of Timanian contraction at ca. 650 Ma (653.2 ± 2.2 Ma granodiorite) and some intrusions show ages coeval with Timanian tectonism at ca. 650–550 Ma (e.g., 596.4 ± 5.1 Ma Øksfjord Gabbro; Roberts *et al.*, 2006; Figure 2).

Alternative interpretations include a link of the Seiland Igneous Province with the Central Iapetus Magmatic Province ( Fichler *et al.*, 2020; Grant *et al.*, 2016; Gumsley *et al.*, 2020; Larsen *et al.*, 2018; Tegner *et al.*, 2019). It is possible that the Seiland Igneous Province is the product of both the Central Iapetus Magmatic Province and subduction of oceanic crust below Baltica during the Timanian Orogeny, the latter potentially strengthening the former. In such model, the Central Iapetus Magmatic Province ( Bingen *et al.*, 1998; Gumsley *et al.*, 2020; Higgins & van Breemen, 1998; Kjøll *et al.*, 2019; Meert *et al.*, 2007; Pease *et al.*, 2008; Tegner *et al.*, 2019), which is oriented perpendicular to the Timanian Orogen and related subduction, may have partly formed as a back-arc basin, i.e., in a similar setting as the South China Sea with the Manila trench ( Ding *et al.*, 2018; Sun, 2016; Qin *et al.*, 2019), or as an impactogenic basin, i.e., extension enhanced by subperpendicular contraction ( Barberi *et al.*, 1982). However, Timanian-related southwest-dipping subduction under Baltica ( Pease *et al.*, 2004) and Laurentia ( Estrada *et al.*, 2018a; Rosa *et al.*, 2016) might be problematic if these blocks were indeed located above the edge of the Jason Large Low-Shear Velocity Province (e.g., Tegner *et al.*, 2019) because it would imply overlapping of major mantle downwelling (subduction) and upwelling processes (related to the Plume Generation Zone at the edges of Large Low-Shear Velocity Provinces; Torsvik *et al.*, 2006). If a southwest-dipping subduction zone indeed existed there in the late Neoproterozoic, then it is inconsistent with the presence of a Large Low Shear Velocity Province below Baltica at that time.

### Implications for the Kalak Nappe Complex and Iapetus Ocean

The rocks of the Kalak Nappe Complex are intruded by the Seiland Igneous Province, which was most likely intruded on the Baltican margin of Iapetus in the late Neoproterozoic. This indicates that the latest Mesoproterozoic–early Neoproterozoic sedimentary rocks of the Kalak Nappe Complex were deposited on the Baltican margin or nearby. This is consistent with the occurrence of late Neoproterozoic dolerite dykes in northeastern Norway ( Nasuti *et al.*, 2015a) both in rock units with presumed Baltican (e.g., Berlevåg Formation in Varangerhalvøya; Kirkland *et al.*, 2008a; Rice *et al.*, 2004) and Laurentian affinities (Kalak Nappe Complex in Nordkinnhalvøya; see red boxes in Nasuti *et al.*, 2015a their Figure 5b). It is also supported by deformation structures in a paragneiss unit in the Kalak Nappe Complex on Hjelmsøya (location in Figure 1b), which suggest three discrete phases of deformation, including (1) development of an intense gneissic foliation in Precambrian gneisses prior to the intrusion of pegmatite dykes, (2) further ductile deformation and shearing of the gneisses and pegmatite dykes and incorporation of the dykes into the foliation, and (3) top-south recumbent folding during Caledonian overprinting ( Ramsay *et al.*, 1979). Based on the 1025 ± 32 Ma age for the youngest detrital grain in the paragneiss on Hjelmsøya ( Kirkland *et al.*, 2008a), and since there are only three major episodes of contractional deformation recorded in Finnmark in the Neoproterozoic–early Paleozoic (possibly Grenvillian–Sveconorwegian, Timanian and Caledonian), the earlier two events recognized on Hjelmsøya may correspond to the Grenvillian–Sveconorwegian and/or Timanian events. Notably, the first event with intrusion of pegmatite dykes is suggested to reflect the Grenvillian Orogeny based on various ca. 980 Ma ages for intrusions and migmatites ( Kirkland *et al.*, 2006b; Kirkland *et al.*, 2008a; Figure 2), and the second event involving shearing of pegmatite dykes and their incorporation into the main foliation is proposed to reflect top-SSW Timanian thrusting along a ductile thrust/shear zone that was later folded into an E–W strike onshore Hjelmsøya during subsequent Caledonian contraction. The involvement of the Kalak Nappe Complex in the Timanian Orogeny is also suggested by the ca. 500 Ma ages obtained for greenschist metamorphism in this unit ( Kirkland *et al.*, 2008b), which imply that it was thrust over Ediacaran metasedimentary rocks of the Lomvatna Group ( Pharaoh *et al.*, 1983) sometime between 650 and 500 Ma. Because of the proximity of the Kalak Nappe Complex both to Laurentia and Baltica (e.g., Corfu *et al.*, 2007; Kirkland *et al.*, 2007a; Slagstad *et al.*, 2006; present study), the Iapetus Ocean was probably narrower than the several thousands of kilometers width typically proposed by paleogeographic reconstructions (e.g., Torsvik & Trench, 1991).

Kirkland *et al.* (2007a) and others argue for an exotic provenance of the Kalak Nappe Complex that would require hundreds of kilometers displacement of various sub-units of the Kalak Nappe Complex in different directions. The similarities of detrital zircons in the Sørøy Succession (upper Kalak Nappe Complex) and the Moine Supergroup (Scotland; provenance from the Grenvillian–Sveconorwegian Orogeny; Bonsor *et al.*, 2012; Glendinning, 1988; Spencer *et al.*, 2015) and in the Sværholt Succession (lower Kalak Nappe Complex) and the Krummedal supracrustal sequence (Greenland) are logical because (1) both Scotland and Greenland were parts of Laurentia in the Neoproterozoic ( Cawood *et al.*, 2004), (2) Baltica and Laurentia were adjacent to each other from the latest Mesoproterozoic to the break-up of Rodinia in the early–mid Neoproterozoic, which initiated at ca. 825 Ma in northern Norway ( Koehl *et al.*, 2018b), and (3) paleomagnetic data for Baltica and Laurentia suggest that these two plates moved together from 750 Ma to 550 Ma before they rifted apart during the opening of Iapetus ( Pease *et al.*, 2008). Hence, the similar detrital signatures may very well be explained by erosion of uplifted portions of the southern Baltican margin of the Grenvillian–Sveconorwegian Orogeny and transport northwards towards Scotland and north-northeastwards towards Finnmark during deposition of the Moine Supergroup and of the Sørøy Succession as suggested by paleocurrent data ( Roberts, 2007), and by transport to the southeast for the Sværholt Succession. In addition, U–Pb dating of detrital zircons show that metasedimentary rocks of the Kalak Nappe Complex have a Timanian signature (i.e., located near Baltica), displaying a clear peak at 570 Ma ( Andresen *et al.*, 2014; Gee *et al.*, 2017), thus further supporting a deposition of the Kalak Nappe Complex near Baltica and the Timanian Orogen.

### The Porsanger Orogeny

The intrusion of the Lillefjord Granite and Revsneshamn Granite at 840 ± 6 Ma and of related pegmatite at 828 ± 4 Ma was dated through U–Pb geochronological analyses on zircon ( Figure 2) and ascribed these magmatic intrusions to syn-kinematic deformation of the Porsanger Orogeny, which is constrained to 1600–840 Ma, i.e., potentially overlapping with the Grenvillian–Sveconorwegian Orogeny ( Corfu *et al.*, 2007; Daly *et al.*, 1991; Kirkland *et al.*, 2006a). However, recent K–Ar geochronological analysis of syn-kinematic illite along brittle faults in Proterozoic basement rocks in adjacent areas of Finnmark near Alta yielded average 1050 ± 15 Ma, 950 ± 15 Ma and 825–805 ± 15 Ma ( Koehl *et al.*, 2018b). The first two average ages directly correlate with contraction and late–post-orogenic collapse related to the Grenvillian–Sveconorwegian Orogeny ( Andersen *et al.*, 2007; Bingen *et al.*, 2008; Corfu *et al.*, 2011; Slagstad *et al.*, 2013; Viola *et al.*, 2011), whereas the latter average age coincides with the initial break-up of Rodinia ( Hartz & Torsvik, 2002; Koehl *et al.*, 2018b; Li *et al.*, 2008) and overlaps with the intrusion of the Lillefjord and Revsneshamn granites and pegmatites ( Kirkland *et al.*, 2006a).

Though the Kalak Nappe Complex is thought to be exotic to Baltica (Laurentian affinities; Corfu *et al.*, 2007; Kirkland *et al.*, 2007a; Slagstad *et al.*, 2006) in the Neoproterozoic, the geochronological ages obtained by Koehl *et al.* (2018b) are relevant for these rocks because, regardless of where they were located at 840–828 Ma, recent paleogeographic reconstructions show that Baltica and Laurentia where located close to each other in the latest Mesoproterozoic (Grenvillian–Sveconorwegian Orogeny; Cawood & Pisarevsky, 2017; Slagstad *et al.*, 2013) and in the latest Neoproterozoic (Timanian Orogeny; Estrada *et al.*, 2018a; Estrada *et al.*, 2018b; Estrada *et al.*, 2018c; Gumsley *et al.*, 2020; Koehl, 2020; Li *et al.*, 2008; Rosa *et al.*, 2016; Tegner *et al.*, 2019). Therefore, it does not matter on which side of Iapetus the rocks of the Kalak Nappe Complex were located because they must have been through the same tectonic events as Laurentia and Baltica throughout the Neoproterozoic, which would imply that the tectonothermal events recorded by Kirkland *et al.* (2007a) in the Kalak Nappe Complex are events of local significance, e.g., related to the Seiland Igneous Province at 570–560 Ma ( Krill & Zwaan, 1987; Roberts *et al.*, 2010), to the Timanian Orogeny at 650–550 Ma ( Olovyanishnikov *et al.*, 2000), and/or to regional plume and rifting events at 825–740 Ma ( Li *et al.*, 2008). Thus, it is more likely that the Lillefjord and Revsneshamn granites, whose age partly overlaps with the ca. 825–805 Ma normal faulting ages in Finnmark ( Koehl *et al.*, 2018b), are related to extensional tectonic processes (as also considered by Kirkland *et al.*, 2006a). For example, they may have formed in an arc-related setting if the subduction related to the Timanian Orogeny had already initiated. Subduction-related magmatism in the mid–late Neoproterozoic may also explain the presence of late Neoproterozoic (680 ± 10 Ma) migmatite in nearby areas of Kvaløya in Finnmark (Eidvågeid migmatite; Corfu *et al.*, 2007; Figure 2).

Another possibility is that the Porsanger Orogeny reflects intra-oceanic contraction during the onset of convergence between Baltica and Laurentia, thus explaining why it is recorded on both Laurentia and Baltica. However, an extensional setting is generally favored for tectonothermal events between 870 and 650 Ma, e.g., break-up of Rodinia ( Callegari *et al.*, 2023; Koehl *et al.*, 2018b; Li *et al.*, 2008). Notably, the Knoydartian/Moranian Orogeny was reinterpreted into an extensional event ( Corfu *et al.*, 2007; Cutts *et al.*, 2009; Soper & Harris, 1997). Thus, it is more likely that the sediments of the lower and upper Kalak Nappe Complex respectively dated to > 980 Ma ( Kirkland *et al.*, 2006a) and ca. 920–910 ± 15–20 Ma to ca. 840 Ma ( Kirkland *et al.*, 2007a), whose age overlaps that of tectonic events related to Grenvillian–Sveconorwegian contraction or collapse at 950 ± 15 Ma ( Koehl *et al.*, 2018b), were deposited in a Grenvillian–Sveconorwegian foreland basin and/or in a late–post Grenvillian–Sveconorwegian collapse basin.

### Implications for the Finnmarkian event

Mild diachronous deformation occurred in Finnmark between the Timanian and Caledonian (Scandian) orogenies at 555–460 Ma as shown by numerous Cambrian–Early Ordovician geochronological ages for metamorphism in rocks of the Kalak Nappe Complex (see references in Rice & Frank, 2003). However, quite a few of these ages were reinterpreted to reflect partial resetting of the K–Ar and Rb–Sr systems and, as concluded by Kirkland *et al.* ( 2006a; 2008b), there are no evidence of major tectonic movements during that period justifying a full orogenic event (Finnmarkian Orogeny *sensu stricto*). Based on the correlation of the Trollfjorden–Komagelva Fault Zone on the western Finnmark Platform and reinterpretation of the tectonic setting during intrusion of the Seiland Igneous Province, the hydrothermal event at 555 ± 15 Ma ( Kirkland *et al.*, 2008b) is more likely to be related to latest Neoproterozoic dyke intrusion in Finnmark ( Rice *et al.*, 2004) and therefore part of the backarc extension phase above the southwest-dipping Timanian subduction zone. Cambrian–Early Ordovician deformation in Finnmark is therefore likely the product of partial resetting of Timanian ages by Caledonian deformation. Alternatively if the ages are genuine, they may reflect the progressive transition from top-SSW Timanian contraction to top-southeast Caledonian (Scandian) contraction, including progressive bending and reorientation of the western portion of the Trollfjorden–Komagelva Fault Zone (Sørøya–Ingøya shear zone) from a NNE-dipping (e.g., in the Varanger Peninsula; Gabrielsen *et al.*, 2022; Herrevold *et al.*, 2009; Siedlecka & Siedlecki, 1967; Siedlecka, 1975) to a northwest-dipping geometry ( Figure 1a–b and Figure 4a–d; Koehl *et al.*, 2018a).

### Geological evolution of northern Norway from the latest Mesoproterozoic to the mid-Paleozoic

**Figure 16.**
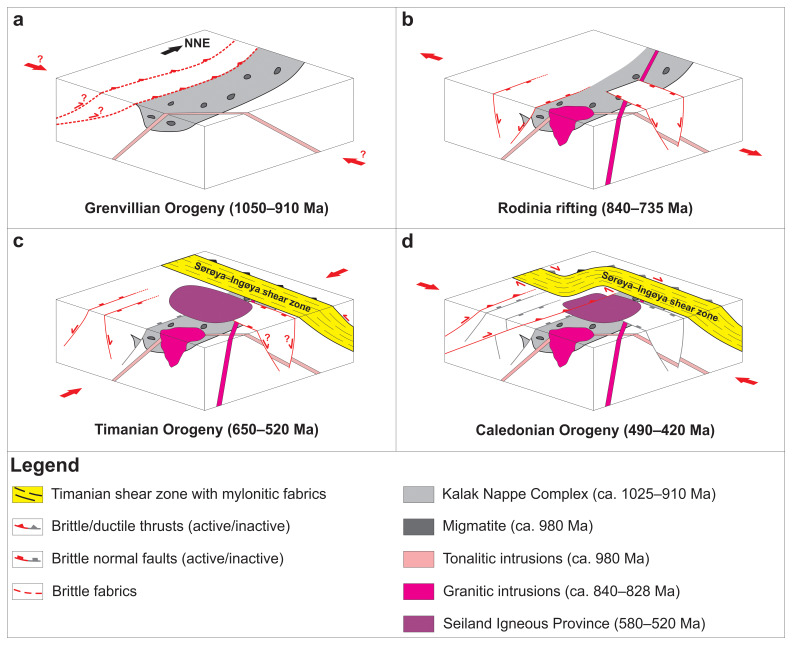
Summary model detailing the tectonic evolution of northern Norway and the southern Barents Sea from the latest Mesoproterozoic to the mid-Paleozoic. ( **a**) Deposition of sedimentary rocks of the Kalak Nappe Complex, possibly in a foreland basin associated with the Grenvillian–Sveconorwegian Orogeny at 1050–910 Ma and their partial migmatization and intrusion by tonalites at ca. 980 Ma. ( **b**) Normal faulting at 825–735 Ma and felsic magmatism at 840–828 Ma (granite and pegmatite, e.g., Lillefjord and Revsneshamn granites) in the Kalak Nappe Complex during the breakup of Rodinia. ( **c**) Top-SSW contraction during the Timanian Orogeny at 650–550 Ma and intrusion of the Seiland Igneous Province in a back-arc basin at 580–520 Ma. ( **d**) Top-southeast thrusting and reworking (folding) and partial reactivation of NNE-dipping Timanian thrusts during the Caledonian Orogeny in the early–mid Paleozoic.

The proximity of the Kalak Nappe Complex to northern Norway in the latest Mesoproterozoic to early Neoproterozoic when deposited between 1025–840 Ma ( Kirkland *et al.*, 2006a; Kirkland *et al.*, 2007a; Kirkland *et al.*, 2008a) and its truncation by intrusions and migmatization at ca. 980 ( Kirkland *et al.*, 2006b; Kirkland *et al.*, 2008a; Figure 2) indicate that northern Norway must also have been affected by the Grenvillian–Sveconorwegian event ( Figure 16a). This is also supported by possible latest Mesoproterozoic–earliest Neoproterozoic faulting ages (i.e., coeval with the Grenvillian–Sveconorwegian Orogeny) in parauthochthonous basement rocks in Finnmark ( Koehl *et al.*, 2018b). The position of Svalbard and of the Barents Sea at that time is still uncertain despite some promising Neoproterozoic paleomagnetic poles for rocks eastern Svalbard ( Michalski *et al.*, 2022). However, evidence of Grenvillian–Sveconorwegian-aged granitic intrusions in Svalbard ( Johansson *et al.*, 2004; Johansson *et al.*, 2005), eastern Greenland ( Leslie & Nutman, 2000; Leslie & Nutman, 2003), the Pearya Terrane ( Trettin, 1987; Trettin *et al.*, 1987), and Taimyr Peninsula ( Pease *et al.*, 2001) suggest that the whole Barents Sea and Svalbard might have already existed in their present configuration in the latest Mesoproterozoic.

In the mid-Tonian, 840–828 Ma granitic and pegmatitic intrusions in the Kalak Nappe Complex ( Kirkland *et al.*, 2006a; Figure 2), 870–840 Ma granitic and gabbroic intrusions in the Seve Nappe in northern Sweden ( Callegari *et al.*, 2023), and ca. 825–805 Ma normal faulting in parauthochthonous basement rocks ( Koehl *et al.*, 2018b) suggest that northern Baltica experienced rifting related to the break-up of Rodinia ( Li *et al.*, 2008; Figure 16b). Rifting must have continued through the late Tonian as suggested by 790–780 Ma and 740–735 Ma ages for brittle–ductile faults in Paleoproterozoic basement rocks in northern Norway ( Torgersen *et al.*, 2014).

In the late Neoproterozoic, northwestern Russia ( Dovzhikova *et al.*, 2004; Gee *et al.*, 2000; Glodny *et al.*, 2004; Kostyuchenko *et al.*, 2006; Kuznetsov *et al.*, 2007; Larionov *et al.*, 2004; Lorenz *et al.*, 2004; Olovyanishnikov *et al.*, 2000; Pease *et al.*, 2004; Remizov, 2006; Remizov & Pease, 2004; Siedlecka & Roberts, 1995), northern Norway ( Dallmeyer & Reuter, 1989; Gorokhov *et al.*, 2001; Siedlecka, 1975), the Barents Sea ( Klitzke *et al.*, 2019; Koehl, 2020; Koehl *et al.*, 2022a; Koehl *et al.*, 2023; Korago *et al.*, 2004; Lopatin *et al.*, 2001), Svalbard ( Birkenmajer, 1991; Bjørnerud, 1990; Bjørnerud *et al.*, 1991; Dallmeyer *et al.*, 1990; Faehnrich *et al.*, 2020; Koglin *et al.*, 2022; Majka *et al.*, 2008; Manecki *et al.*, 1998; Peucat *et al.*, 1989), and probably northern Greenland ( Estrada *et al.*, 2018a; Rosa *et al.*, 2016) and the Pearya Terrane ( Estrada *et al.*, 2018b) were involved in top-SSW contraction and southwest-directed subduction related to the Timanian Orogeny ( Figure 16c). Timanian subduction processes led to the intrusion of the Seiland Igneous Province from ca. 650 Ma to 530–520 Ma ( Krauskopf, 1954; Roberts *et al.*, 2006; Roberts *et al.*, 2010; Robins & Gardner, 1975; Speedyman, 1983; Sturt & Ramsay, 1965), of dolerite dykes on the Varanger Peninsula and in the Kalak Nappe Complex at 577 Ma and 550 Ma ( Andersen & Sundvoll, 1995; Nasuti *et al.*, 2015a; Rice *et al.*, 2004), and to the Finnmarkian hydrothermal event at 555 Ma ( Kirkland *et al.*, 2008b; Figure 2) in a backarc setting, and, eventually, to the rifting of the Iapetus Ocean ( Figure 16c; Bingen *et al.*, 1998; Gumsley *et al.*, 2020; Higgins & van Breemen, 1998; Kjøll *et al.*, 2019; Meert *et al.*, 2007; Pease *et al.*, 2008; Tegner *et al.*, 2019), although with a significantly narrower width than that proposed by previous studies (e.g., Torsvik & Trench, 1991).

Rifting continued through the early Paleozoic, until NW–SE-oriented convergence in the Middle Ordovician–Silurian resulted in the Caledonian Orogeny (e.g., Binns & Gayer, 1980; Corfu *et al.*, 2011; Kirkland *et al.*, 2007b; Townsend, 1987; Figure 16d). Caledonian contraction involved the reworking of Timanian thrusts in the Barents Sea, Svalbard, and northern Norway into NNE-plunging ( Figure 7– Figure 14) and into dome- and trough-shaped folds ( Gabrielsen *et al.*, 2022; Koehl, 2020; Koehl *et al.*, 2022a; Koehl *et al.*, 2023; Siedlecka & Siedlecki, 1971), and the reactivation of folded portion of Timanian thrusts (e.g., Trollfjorden–Komagelva Fault Zone on the western Finnmark Platform) as top-southeast thrusts ( Figure 4d and Figure 16d).

## Conclusions

- 1) Top-SSW Timanian thrust systems on the Finnmark Platform were folded into NNE-plunging and dome- and trough-shaped folds during the Caledonian Orogeny.
- 2) On the western Finnmark Platform, the NNE-dipping Timanian thrust front, potentially the Trollfjorden–Komagelva Fault Zone, bends into a northwest-dipping geometry and was locally reactivated as a top-southeast thrust during the Caledonian Orogeny. Alternatively, the Timanian thrust front is located just off the coast of Finnmark.
- 3) The Seiland Igneous Province most likely formed in a backarc setting near the Timanian thrust front (Trollfjorden–Komagelva Fault Zone).
- 4) The Kalak Nappe Complex is intruded by the Seiland Igneous Province, which reaches a depth superior to that of the maximum thickness of the Kalak Nappe Complex. Metasedimentary rocks of the Kalak Nappe Complex therefore deposited along the Baltican margin of the Iapetus Ocean.
- 5) The proximity of the Kalak Nappe Complex both to Baltica and Laurentia suggests that the Iapetus Ocean was much narrower than the several thousands of kilometers width commonly proposed.
- 6) Magmatism related to the Porsanger Orogeny is more likely to have occurred in an extensional setting during the initial breakup of Rodinia at ca. 840–805 Ma, and lowermost Neoproterozoic sedimentary rocks of the Kalak Nappe Complex probably deposited in basins formed during Grenvillian–Sveconorwegian contraction or related late–post-orogenic collapse.
- 7) The thermal event at the Neoproterozoic–Cambrian transition in eastern Finnmark initially associated with the Finnmarkian event is more likely related to backarc magmatism over the southwest-directed Timanian subduction zone. Alternatively, the Finnmarkian event may represent the transition from top-SSW Timanian to top-southeast Caledonian (Scandian) deformation.

## Data Availability

The seismic reflection data used in the present study are from the Norwegian National Data Repository for Petroleum Data (DISKOS database), the Norwegian Defense Research Establishment, and TGS (FP13). The data may be accessed for research purpose by contacting the Norwegian Offshore Directorate at https://www.sodir.no/om-oss/kontakt-oss/, the Norwegian Defense Research Establishment at https://www.ffi.no/en/about-ffi/contact-us, and TGS at https://www.tgs.com/contact-us. The interpreted magnetic data are from the Geological Survey of Norway. Access to the onshore data (MINN project) can be obtained by contacting the Geological Survey of Norway at https://www.ngu.no/om-ngu/kontakt-ngu. Regarding the offshore dataset, in addition to contacting the NGU, a small part of the data (over the Hammerfest Basin) is the property of Equinor, which may be contacted at https://www.equinor.com/about-us/contact-us to get access to the entire dataset (BASAR project). The onshore topographic and nearshore bathymetric data of the Norwegian Mapping Authority may be obtained by contacting the organization at https://www.kartverket.no/en/about-kartverket/contact-us. DataverseNO: Extended data for "Caledonian reactivation and reworking of Timanian thrust systems and implications for latest Mesoproterozoic to mid-Paleozoic tectonics and magmatism in northern Baltica", doi.org/10.18710/YQNSGQ ( Koehl, 2024) The project contains the following extended data: -00_ReadMe.txt -Koehl_Stokmo_(Extended_data).docx (high-resolution versions of the figures) -Koehl_Stokmo_(Extended_data).pdf (pdf version of the above-mentioned document) The data are available under the terms of the Creative Commons Zero “No rights reserved” data waiver (CC0 1.0 Public domain dedication).

## References

[ref-1] Agyei-Dwarko NY Augland LE Andresen A : The Heggmovatn supracrustals, North Norway—A late mesoproterozoic to early neoproterozoic (1050-930 Ma) terrane of Laurentian origin in the Scandinavian Caledonides. *Precambrian Res.* 2012;212–213:245–262. 10.1016/j.precamres.2012.06.008

[ref-6] Andresen A Agyei-Dwarko N Steltenpohl M : Detrital zircon data support a Timanian origin for the Kalak Nappe Complex, North Norwegian Caledonides.EGU General Assembly, 27 April-2 May, 2014, Vienna, Austria. *Geophysical research Abstracts.* 2014;16: EGU2014–4848. Reference Source

[ref-2] Andersen TB : The structure of the Magerøy Nappe, Finnmark, North Norway. *Norges geologiske undersøkelse.* 1981;363:1–23. Reference Source

[ref-3] Andersen TB : The stratigraphy of the Magerøy Supergroup, Finnmark, north Norway. *Norges geologiske undersøkelse.* 1984;395:25–37. Reference Source

[ref-5] Andersen T Graham S Sylvester AG : Timing and tectonic significance of Sveconorwegian A-type granitic magmatism in Telemark, Southern Norway: new results from laser-ablation ICPMS U-Pb dating of zircon. *Norge Geologiske undersøkelse bulletin.* 2007;447:17–31. Reference Source

[ref-4] Andersen TB Sundvoll B : Neodymium isotope systematics of the mantle beneath the Baltic shield: evidence for depleted mantle evolution since the Archaean. *Lithos.* 1995;35(3–4):235–243. 10.1016/0024-4937(94)00053-5

[ref-7] Appleyard EC : Preliminary description of the geology of the Donnesfjord area, Sørøy. *Norges geologiske undersøkelse.* 1965;231:144–164.

[ref-8] Augland LE Andresen A Corfu F : The Bratten-Landegode gneiss complex: a fragment of Laurentian continental crust in the uppermost allochthon of the Scandinavian Caledonides.In: *New Perspectives on the Caledonides of Scandinavia and Related Areas*. edited by: Corfu, F., Gasser, D. and Chew, D. M., Geol Soc Spec Publ. 2013;390:633–654. 10.1144/SP390.1

[ref-9] Barberi F Santacroce R Varet J : Chemical aspects of rift magmatism. *Continental and Oceanic Rifts Geodynamics Series.* 1982;8:223–258. Reference Source

[ref-10] Beckholmen M Glodny J : Timanian blueschist-facies metamorphism in the Kvarkush metamorphic basement, Northern Urals, Russia.In: *The Neoproterozoic Timanide Orogen of Eastern Baltica*. edited by: Gee, D. G. and Pease, V., Geological Society of London Memoirs, 2004;30(1):125–134. 10.1144/GSL.MEM.2004.030.01.11

[ref-13] Bergh SG Corfu F Priyatkina N : Multipple pst-Svecofennian 1750-1560 Ma pegmatite dykes in Archaean-Palaeoproterozoic rocks of the West Troms Basement Complex, North Norway: geological significance and regional implications. *Precambrian Res.* 2015;266:425–439. 10.1016/j.precamres.2015.05.035

[ref-12] Bergh SG Kullerud K Armitage PEB : Neoarchean to Svecofennian tectono-magmatic evolution of the West Troms Basement Complex, North Norway. *Norwegian Journal of Geology.* 2010;90(1–2):21–48. Reference Source

[ref-11] Bergh SG Torske T : Palaeovolcanology and tectonic setting of a proterozoic metatholeiitic sequence near the baltic shield margin, Northern Norway. *Precambrian Res.* 1988;39(4):227–246. 10.1016/0301-9268(88)90021-6

[ref-14] Bergström J Gee DG : The cambrian in scandinavia.In: *The Caledonide Orogen—Scandinavia and Related Areas.*edited by: Gee, D. G. and Sturt, B. A.1985;247–271. Reference Source

[ref-16] Bingen B Demaiffe D van Breemen O : The 616 Ma Old Egersund Dike Swarm, SW Norway, and Late Neoproterozoic Opening of the Iapetus Ocean. *J Geol.* 2008;106:565–574. 10.1086/516042

[ref-15] Bingen B Nordgulen Ø, Viola G : A four-phase model for the Sveconorwegian orogeny, SW Scandinavia. *Norw J geol.* 1998;88:43–72. Reference Source

[ref-17] Binns RE Gayer RA : Silurian or upper ordovician fossils at guolasjav’ri Troms, Norway. *Nature.* 1980;284:53–55. 10.1038/284053a0

[ref-18] Birkenmajer K : The Jarlsbergian unconformity (Proterozoic/Cambrian boundary) and the problem of Varangian tillites in South Spitsbergen. *Pol Polar Res.* 1991;12(3):269–278. Reference Source

[ref-19] Bjørnerud M : An upper proterozoic unconformity in northern wedel jarlsberg land, southwest Spitsbergen: lithostratigraphy and tectonic implications. *Polar Res.* 1990;8(2):127–139. 10.3402/polar.v8i2.6809

[ref-20] Bjørnerud M Decker PI Craddock C : Reconsidering Caledonian deformation in southwest Spitsbergen. *Tectonics.* 1991;10(1):171–190. 10.1029/90TC02396

[ref-21] Bonsor HC Strachan RA Prave AR : Sedimentology of the early neoproterozoic morar group in northern Scotland: implications for basin models and tectonic setting. *J Geol Soc.* London.2012;169(1):53–65. 10.1144/0016-76492011-039

[ref-22] Braathen A Davidsen B : Structure and stratigraphy of the Palaeoproterozoic Karasjok Greenstone Belt, north Norway - regional implications. *Nor Geol Tiddsskrift.* 2000;80(1):33–50. 10.1080/002919600750042663

[ref-250] Bugge T Mangerud G Elvebakk G : The upper paleozoic succession on the Finnmark platform, Barents Sea. *Norsk Geol Tidsskr.* 1995;75:3–30. Reference Source

[ref-230] Callegari R Kosminska K Barnes CJ : Early neoproterozoic magmatism and Caledonian metamorphism recorded by the mårma terrane, seve nappe complex, northern Swedish Caledonides. *J Geol Soc.* 2023;180:5. 10.1144/jgs2022-092

[ref-24] Cawood PA Nemchin AA Strachan RA : Laurentian provenance and tectonic setting for the upper moine supergroup, Scotland, constrained by detrital zircons from the Loch Eil and Glen Urquhart successions. *J Geol Soc.* London.2004;161:863–874. 10.1144/16-764903-117

[ref-23] Cawood PA Pisarevsky SA : Laurentia-Baltica-Amazonia relations during Rodinia assembly. *Precambrian Res.* 2017;292:386–397. 10.1016/j.precamres.2017.01.031

[ref-25] Chauvet A Séranne M : Extension-parallel folding in the Scandinavian Caledonides: implications for late-orogenic processes. *Tectonophysics.* 1994;238(1–4):31–54. 10.1016/0040-1951(94)90048-5

[ref-29] Corfu F Andersen TB Gasser D : The Scandinavian Caledonides: main features, conceptual advances and critical questions. In: *New Perspectives on the Caledonides of Scandinavia and Related Areas*. edited by: Corfu, F., Gasser, D. and Chew, D.M. Geological Society, London, Special Publications,2014;390:9–43. 10.1144/SP390.25

[ref-28] Corfu F Gerber M Andersen TB : Age and significance of grenvillian and silurian orogenic events in the Finnmarkian Caledonides, northern Norway. *Can J Earth Sci.* 2011;48(2):419–440. 10.1139/E10-043

[ref-26] Corfu F Ravna EJK Kullerud K : A late ordovician U-Pb age for the tromsø nappe eclogites, uppermost allochthon of the Scandinavian Caledonides. *Contrib Mineral Petrol.* 2003;145:502–513. 10.1007/s00410-003-0466-x

[ref-27] Corfu F Roberts RJ Torsvik TH : Peri-Gondwanan elements in the Caledonian nappes of Finnmark, northern Norway: implications for the paleogeographic framework of the Scandinavian Caledonides. *Am J Sci.* 2007;307(2):434–458. 10.2475/02.2007.05

[ref-225] Corfu F Torsvik TH Andersen TB : Early Silurian mafic-ultramafic and granitic plutonism in contemporaneous flysch, Magerøy, northern Norway: U-Pb ages and regional significance. *J Geol Soc.* London,2006;163:291–301. 10.1144/0016-764905-014

[ref-30] Crowell JC : The san andreas fault system through time. *J Geol Soc.* London.1979;136:293–302. 10.1144/gsjgs.136.3.0293

[ref-31] Cutts KA Hand M Kelsey DE : Orogenic versus extensional settings for regional metamorphism; knoydartian events in the moine supergroup revisited. *J Geol Soc.* London.2009;166:201–204. 10.1144/0016-76492008-015

[ref-33] Dallmeyer RD Peucat JJ Ohta Y : Tectonothermal evolution of contrasting metamorphic complexes in northwest Spitsbergen (Biskayerhalvøya): evidence from ^40^Ar/ ^39^Ar and Rb-Sr mineral ages. *GSA Bull.* 1990;102(5):653–663. 10.1130/0016-7606(1990)102<0653:TEOCMC>2.3.CO;2

[ref-32] Dallmeyer RD Reuter A : ^40^Ar/ ^39^Ar whole-rock dating and the age of cleavage in the Finnmark autochthon, northernmost Scandinavian Caledonides. *Lithos.* 1989;22(3):213–227. 10.1016/0024-4937(89)90057-1

[ref-34] Daly JS Aitcheson SJ Cliff RA : Geochronological evidence from discordant plutons for a late proterozoic orogen in the Caledonides of Finnmark, northern Norway. *J Geol Soc.* London.1991;148(1):29–40. 10.1144/gsjgs.148.1.0029

[ref-35] Dickinson WR : Interpreting detrital modes of graywacke and arkose. *J Sediment Res.* 1970;40(2):695–707. 10.1306/74d72018-2b21-11d7-8648000102c1865d

[ref-36] Ding W Sun Z Dadd K : Structures within the oceanic crust of the central South China Sea basin and their implications for oceanic accretionary processes. *Earth Planet Sci Lett.* 2018;488:115–125. 10.1016/j.epsl.2018.02.011

[ref-37] Dovzhikova E Pease V Remizov D : Neoproterozoic island arc magmatism beneath the pechora basin, NW Russia. *GFF.* 2004;126(4):353–362. 10.1080/11035890401264353

[ref-38] Draut AE Clift PD : Geochemical evolution of arc-magmatism during arc-continent collision, South Mayo, Ireland. *Geology.* 2001;29(6):543–546. 10.1130/0091-7613(2001)029<0543:GEOAMD>2.0.CO;2

[ref-39] Ebbestad JOR Högström AES Palacios T : Biostratigraphy and palaeontology of the lower cambrian duolbagáisá formation on the Digermulen Peninsula, Arctic Norway. *International Conference on Arctic Margins*, 11-14 June 2018, Stockholm, Sweden,2018. Reference Source

[ref-251] Eldholm O Ewing J : Marine geophysical survey in the southwestern Barents Sea. *J Geophys Res.* 1971;76(17):3832–3841. 10.1029/JB076i017p03832

[ref-40] Elvevold S Reginiussen H Krogh EJ : Reworking of deep-seated gabbros and associated contact metamorphosed paragneisses in the south-eastern part of the Seiland Igneous Province, Northern Norway. *J Metamorph Geol.* 1994;12(4):539–556. 10.1111/j.1525-1314.1994.tb00041.x

[ref-43] Estrada S Koglin N Gerdes A : Ediacaran (Timanian) island arc fragments of Baltica at the northcoast of Laurentia. *GeoBonn, 2–6 2018.* Bonn, Germany,2018c. 10.13140/RG.2.2.31192.11521

[ref-42] Estrada S Mende K Gerdes A : Proterozoic to cretaceous evolution of the western and central Pearya Terrane (Canadian High Arctic). *J Geodyn.* 2018b;120:45–76. 10.1016/j.jog.2018.05.010

[ref-41] Estrada S Tessensohn F Sonntag BL : A Timanian island-arc fragment in North Greenland: the midtkap igneous suite. *J Geodyn.* 2018a;118:140–153. 10.1016/j.jog.2018.01.015

[ref-44] Faber C : Mountain building processes in the northern Norwegian Caledonides.Ph.D. Thesis, UiT The Arctic University of Norway in Tromsø, Tromsø, Norway,2018. Reference Source

[ref-45] Faber C Stünitz H Gasser D : Anticlockwise metamorphic pressure–temperature paths and nappe stacking in the Reisa Nappe complex in the Scandinavian Caledonides, northern Norway: evidence for weakening of lower continental crust before and during continental collision. *Solid Earth.* 2019;10(1):117–148. 10.5194/se-10-117-2019

[ref-46] Faehnrich K Majka J Schneider D : Geochronological constraints on Caledonian strike-slip displacement in Svalbard, with implications for the evolution of the Arctic. *Terra Nova.* 2020;32(4):290–299. 10.1111/ter.12461

[ref-47] Fichler C Pouliquen G Pastore Z : Thought-provoking features in the SW Barents Sea from 3D geophysical modelling – from salt domes to crustal peridotites. *34 ^th^ Nordic Geological Winter Meeting, Oslo, Norway, Abstracts and Proceedings of the Geological Society of Norway*,2020.

[ref-48] Fossen H Teyssier C Whitney DL : Transtensional folding. *J Struct Geol.* 2013;56:89–102. 10.1016/j.jsg.2013.09.004

[ref-49] Freeman B Klemperer SL Hobbs RW : The deep structure of northern England and the Iapetus Suture zone from BIRPS deep seismic reflection profiles. *J Geol Soc.* London,1988;145:727–740. 10.1144/gsjgs.145.5.0727

[ref-50] Gabrielsen RH : Long-lived fault zones and their influence on the tectonic development of the southwestern Barents Sea. *J Geol Soc.* London,1984;141:651–662. 10.1144/gsjgs.141.4.0651

[ref-51] Gabrielsen RH Færseth RB : The inner shelf of North Cape, Norway and its implications for the Barents Shelf-Finnmark Caledonide boundary. A comment. *Norsk Geologisk Tidsskrift.* 1989;69:57–62. Reference Source

[ref-52] Gabrielsen RH Færseth RB Jensen LN : Structural elements of the Norwegian continental shelf, Part I: the Barents Sea Region. *Norwegian Petroleum Directorate Bulletin*.1990;6:33. Reference Source

[ref-53] Gabrielsen RH Roberts D Gjelsvik T : Double-folding and thrust-front geometries associated with the Timanian and Caledonian orogenies in the Varanger Peninsula, Finnmark, North Norway. *J Geol Soc.* London,2022;179(6). 10.1144/jgs2021-153

[ref-54] Gaidies F Heldwein OKA Yogi MTAG : Testing the equilibrium model: an example from the Caledonian Kalak Nappe Complex (Finnmark, Arctic Norway). *J Metamorph Geol.* 2021;40(5):859–886. 10.1111/JMG.12648

[ref-55] Gasser D Jerabek P Faber C : Bhaviour of geochronometers and timing of metamorphic reactions during deformation at lower crustal conditions: phase equilibrium modelling and U–Pb dating of zircon, monazite, rutile and titanite from the Kalak Nappe Complex, Northern Norway. *J Metamorph Geol.* 2015;33(5):513–534. 10.1111/jmg.12131

[ref-56] Gayer RA Hayes SJ Rice AHN : The structural development of the Kalak Nappe Complex of Eastern and Central Porsangerhalvøya, Finnmark, Norway. *Norges Geologiske Undersøkelse Bulletin.* 1985;400:67–87.

[ref-57] Gayer RA Rice AHN Roberts D : Restoration of the Caledonian Baltoscandian margin from balanced cross-secyions: the problem of excess continental crust. *Trans R Soc Edinb Earth Sci.* 1987;78(3):197–217. 10.1017/S026359330001110X

[ref-59] Gee DG Andréasson PG Li Y : Baltoscandian margin, Sveconorwegian crust lost by subduction during Caledonian collisional orogeny. *GFF.* 2017;139(1):36–51. 10.1080/11035897.2016.1200667

[ref-58] Gee DG Beliakova L Pease V : New single zircon (pb-evaporation) ages from vendian intrusions in the basement beneath the pechora basin, Northeastern Baltica. *Polarfoschung.* 2000;68:161–170. Reference Source

[ref-226] Geldart LP Sheriff RE : Problems in exploration seismology and their solutions. *Society of Exploration Geophysicists, Geophysical References Series.* 2004;181–220. 10.1190/1.9781560801733

[ref-60] Gernigon L Brönner M : Late Palaeozoic architecture and evolution of the southwestern Barents Sea: insights from a new generation of aeromagnetic data. *J Geol Soc.* London,2012;169:449–459. 10.1144/0016-76492011-131

[ref-61] Gernigon L Brönner M Roberts D : Crustal and basin evolution of the southwestern Barents Sea: from Caledonian orogeny to continental breakup. *Tectonics.* 2014;33(4):347–373. 10.1002/2013TC003439

[ref-227] Gernigon L Brönner M Dumais MA : Basement inheritance and salt structure in the SE Barents Sea: insights from new potential field data. *J Geodyn.* 2018;119:82–106. 10.1016/j.jog.2018.03.008

[ref-62] Geul JJC : Preliminary report on the geology of east Magerøy, with geological map of east Magerøy, scale 1: 50,000. Unpublished manuscript, Norges geologiske undersøkelse Archives, Trondheim.1958;20.

[ref-63] Glendinning NRW : Sedimentary structures and sequences within a late Proterozoic tidal shelf deposit: the Upper Morar Psammite Formation of Northwestern Scotland.In: Winchester JA, eds.: *Later Proterozoic Stratigraphy of the Northern Atlantic Regions*. Blackie, Glasgow,1988;14–31. 10.1007/978-1-4615-7344-9_2

[ref-64] Glodny J Pease V Montero P : Protolith ages of eclogites, Marun-Keu Complex, Polar Urals, Russia: implications for the pre- and early Uralina evolution of the Northeastern European continental margin.In: Gee DG, Pease V, eds.: *The Neoproterozoic Timanide Orogen of Eastern Baltica*. Geological Society of London, Memoirs,2004;30:207–232. 10.1144/GSL.MEM.2004.030.01.09

[ref-65] Gorokhov IM Siedlecka A Roberts D : Rb-Sr dating of diagenetic illite in Neoproterozoic shales, Varanger Peninsula, Northern Norway. *Geol Mag.* 2001;138(5):541–562. 10.1017/S001675680100574X

[ref-66] Grant TB Larsen RB Anker-Rasch L : Anatomy of a deep crustal volcanic conduit system; The Reinfjord Ultramafic Complex, Seiland Igneous Province, Northern Norway. *Lithos.* 2016;252–253:200–215. 10.1016/j.lithos.2016.02.020

[ref-67] Griffin WL Sturt BA O’Neill CJ : Intrusion and contamination of high-temperature dunitic magma: the Nordre Bumandsfjord pluton, Seiland, Arctic Norway. *Contrib Mineral Petrol.* 2013;165:903–930. 10.1007/s00410-012-0841-6

[ref-68] Guise PG Roberts D : Devonian ages from ^40^Ar/ ^39^Ar dating of plagioclase in dolerite dykes, eastern Varanger Peninsula, North Norway. *Norges geologiske undersøkelse.* 2002;440:27–37. Reference Source

[ref-69] Gumsley A Manby G Domanska-Siuda J : Caught between two continents: first identification of the Ediacaran Central Iapetus Magmatic Province in Western Svalbard with palaeogeographic implications during final Rodinia breakup. *Precambrian Res.* 2020;341: 105622. 10.1016/j.precamres.2020.105622

[ref-70] Hartz EH Torsvik TH : Baltica upside down: a new plate tectonic model for Rodinia and the Iapetus Ocean. *Geology.* 2002;30(3):255–258. 10.1130/0091-7613(2002)030<0255:BUDANP>2.0.CO;2

[ref-71] Hellmann FJ Gee DG Gjelsvik T : Provenance and tectonic implications of Palaeoproterozoic (c. 1740 Ma) quartz porphyry clasts in the basal Old Red Sandstone (Liljeborgfjellet Conglomerate Formation) of Northwestern Svalbard’s Caledonides. *Geological Magazine.* 1998;135(6):755–768. Reference Source

[ref-72] Henderson IHC Viola G Nasuti A : A new tectonic model for the Palaeoproterozoic Kautokeino Greenstone Belt, northern Norway, based on high-resolution airborne magnetic data and field structural analysis and implications for mineral potential. *Norwegian Journal of Geology.* 2015;95:1–26.

[ref-73] Henningsmoen G : Cambro-Silurian fossils in Finnmark, Northern Norway. *Norges geologiske undersøkelse.* 1961;213:93–95. Reference Source

[ref-74] Herrevold T Gabrielsen RH Roberts D : Structural geology of the southeastern part of the Trollfjorden-Komagelva Fault Zone, Varanger Peninsula, Finnmark, North Norway. *Norwegian Journal of Geology.* 2009;89:305–325. Reference Source

[ref-75] Higgins MD van Breemen O : The Age of the Sept Iles Layered Mafic Intrusion, Canada: Implications for the Late Neoproterozoic/Cambrian History of Southeastern Canada. *J Geol.* 1998;106:421–431. 10.1086/516033

[ref-76] Högström AES Jensen S Ebbestad JO : Expanding the Ediacaran biota on the Digermulen Peninsula, Arctic Norway. In: *International Symposium in the Edicaran-Cambrian Transition.*20-22 June 2017, St. John’s, Newfoundland, Canada,2017.

[ref-77] Indrevær K Bergh SG Koehl JB : Post-Caledonian brittle fault zones on the hyperextended SW Barents Sea margin: New insights into onshore and offshore margin architecture. *Norwegian Journal of Geology.* 2013;93:167–188. Reference Source

[ref-78] Jakobsson M Mayer L Coackley B : The International Bathymetric Chart of the Arctic Ocean (IBCAO) Version 3.0. *Geophys Res Lett.* 2012;39(12): L12609. 10.1029/2012GL052219

[ref-79] Janecke SU Markowski DK Evans JP : Durmid ladder structure and its implications for the nucleation sites of the next M >7.5 earthquake on the San Andreas fault or Brawley seismic zone in southern California. *Lithosphere.* 2018;10(5):602–631. 10.1130/L629.1

[ref-80] Jensen S Högström AES Høyberget M : Trace fossils across the Ediacaran-Cambrian boundary on the Digermulen Peninsula, Arctic Norway. In: *International Symposium in the Edicaran-Cambrian Transition.*20–22 June 2017, St. John’s, Newfoundland, Canada,2017.

[ref-81] Johansen SE Henningsen T Rundhovde E : Continuation of the Caledonides north of Norway: seismic reflectors within the basement beneath the southern Barents Sea. *Mar Pet Geol.* 1994;11(2):190–201. 10.1016/0264-8172(94)90095-7

[ref-82] Johansson Å Gee DG : The late Palaeoproterozoic Eskolabreen granitoids of southern Ny Friesland, Svalbard Caledonides - geochemistry, age and origin. *GFF.* 1999;121(2):113–126. 10.1080/11035899901212113

[ref-86] Johansson Å Gee DG Larionov AN : Greenvillian and Caledonian evolution of eastern Svalbard - a tale of two orogenies. *Terra Nova.* 2005;17:317–325. 10.1144/gsl.mem.2004.030.01.17

[ref-85] Johansson Å Larionov AN Gee DG : Greenvillian and Caledonian tectono-magmatic activity in northeasternmost Svalbard.In: Gee DG, Pease V, eds.: *The Neoproterozoic Timanide Orogen of Eastern Baltica*. Geological Society of London. Memoirs,2004;30:207–232. 10.1144/gsl.mem.2004.030.01.17

[ref-83] Johansson Å Larionov AN Tebenkov AM : Grenvillian magmatism of western and central Nordaustlandet, northeastern Svalbard. *Trans R Soc Edinb Earth Sci.* 2000;90(3):221–254. 10.1017/S0263593300002583

[ref-84] Johansson Å Maluski H Gee DG : Ar-Ar dating of Caledonian and Grenvillian rocks from northeasternmost Svalbard - evidence of two stages of Caledonian tectonothermal activity in the high Arctic. *Nor J Geol.* 2001;81:263–281. Reference Source

[ref-228] Kallweit RS Wood LC : The limits of resolution of zero-phase wavelets. *Geophysics.* 1982;47(7):1035–1046. 10.1190/1.1441367

[ref-92] Kirkland CL Daly JS Chew DM : The Finnmarkian Orogeny revisited: an isotopic investigation in eastern Finnmark, Arctic Norway. *Tectonophysics.* 2008a;460(1–4):158–177. 10.1016/j.tecto.2008.08.001

[ref-89] Kirkland CL Daly JS Eide EA : The structure and timing of lateral escape during the Scandian Orogeny: a combined strain and geochronological investigation in Finnmark, Arctic Norwegian Caledonides. *Tectonophysics.* 2006b;425(1–4):159–189. 10.1016/j.tecto.2006.08.001

[ref-90] Kirkland CL Daly JS Eide EA : Tectonic evolution of the Arctic Norwegian Caledonides from a texturally- and structurally-constrained multi-isotopic (Ar-Ar, Rb-Sr, Sm-Nd, U-Pb) study. *Am J Sci.* 2007a;307(2):459–526. 10.2475/02.2007.06

[ref-87] Kirkland CL Daly JS Whitehouse MJ : Early Silurian magmatism and the Scandian evolution of the Kalak Nappe Complex, Finnmark, Arctic Norway. *J Geol Soc.* London.2005;162:985–1003. 10.1144/0016-764904-124

[ref-88] Kirkland CL Daly JS Whitehouse MJ : Granitic magmatism of Grenvillian and late Neoproterozoic age in Finnmark, Arctic Norway—Constraining pre-Scandian deformation in the Kalak Nappe Complex. *Precambrian Res.* 2006a;145(1–2):24–52. 10.1016/j.precamres.2005.11.012

[ref-91] Kirkland CL Daly JS Whitehouse MJ : Provenance and terrane evolution of the Kalak Nappe Complex, Norwegian caledonides: implications for neoproterozoic paleogeography and tectonics. *J Geol.* 2007b;115(1):21–41. 10.1086/509247

[ref-93] Kirkland CL Daly JS Whitehouse MJ : Basement-cover relationships of the Kalak Nappe Complex, Arctic Norwegian Caledonides and constraints on Neoproterozoic terrane assembly in the North Atlantic region. *Precambrian Res.* 2008b;160(3–4):245–276. 10.1016/j.precamres.2007.07.006

[ref-94] Kjøll HJ Andersen TB Corfu F : Timing of breakup and thermal evolution of a pre-caledonian neoproterozoic exhumed magma-rich rifted margin. *Tectonics.* 2019;38(6):1843–1862. 10.1029/2018TC005375

[ref-95] Klitzke P Franke D Ehrhardt A : The paleozoic evolution of the olga basin region, northern barents sea: a link to the timanian orogeny. *Geochem Geophy Geosy.* 2019;20(2):614–629. 10.1029/2018GC007814

[ref-96] Koehl JBP : Impact of Timanian thrusts on the Phanerozoic tectonic history of Svalbard.Keynote lecture, EGU General Assembly, May 3 ^rd^-8 ^th^2020, Vienna, Austria,2020. 10.5194/egusphere-egu2020-2170

[ref-97] Koehl JBP : Early Cenozoic Eurekan strain partitioning and decoupling in central Spitsbergen, Svalbard. *Solid Earth.* 2021;12(5):1025–1049. 10.5194/se-12-1025-2021

[ref-252] Koehl JBP : Extended data for "Caledonian reactivation and reworking of Timanian thrust systems and implications for latest Mesoproterozoic to mid-paleozoic tectonics and magmatism in northern Baltica".DataverseNo.2024. 10.18710/YQNSGQ PMC11751614 39845433

[ref-98] Koehl JBP Bergh SG Henningsen T : Middle to Late Devonian-Carboniferous collapse basins on the Finnmark Platform and in the southwesternmost Nordkapp basin, SW Barents Sea. *Solid Earth.* 2018a;9(2):341–372. 10.5194/se-9-341-2018

[ref-100] Koehl JBP Bergh SG Osmundsen PT : Late Devonian-Carboniferous faulting and controlling structures and fabrics in NW Finnmark. *Nor J Geol.* 2019;99(3):459–499. 10.17850/njg99-3-5

[ref-103] Koehl JBP Bergh SG Sylvester AG : Tectonic evolution of the Indio Hills segment of the San Andreas fault in southern California, southwestern USA. *Solid Earth.* 2022c;13(8):1169–1190. 10.5194/se-13-1169-2022

[ref-99] Koehl JBP Bergh SG Wemmer K : Neoproterozoic and post-Caledonian exhumation and shallow faulting in NW Finnmark from K-Ar dating and *p/T* analysis of fault rocks. *Solid Earth.* 2018b;9(4):923–951. 10.5194/se-9-923-2018

[ref-101] Koehl JBP Magee C Anell I : Impact of Timanian thrust systems on the late Neoproterozoic–Phanerozoic tectonic evolution of the Barents Sea and Svalbard. *Solid Earth.* 2022a;13(1):85–115. 10.5194/se-13-85-2022

[ref-102] Koehl JBP Marshall JEA Lopes G : The timing of the Svalbardian Orogeny in Svalbard: a review. *Solid Earth.* 2022b;13(8):1353–1370. 10.5194/se-13-1353-2022

[ref-104] Koehl JBP Polonio I Rojo LA : The nature of basement rocks in the Loppa High revealed by new 3D seismic attribute and spectral decomposition.Friday Seminar, 13 May 2022, UiT The Arctic University of Norway in Tromsø, Tromsø, Norway,2022d. Reference Source

[ref-105] Koehl JBP Polonio I Rojo LA : Timanian Fold-and-Thrust Belt and Caledonian Overprint in the Selis Ridge Imaged by New 3D Seismic Attributes and Spectral Decomposition. *Tektonika.* 2023;1(1):75–100. 10.55575/tektonika2023.1.1.9

[ref-106] Koglin N Läufer A Piepjohn K : Paleozoic sedimentation and Caledonian terrane architecture in NW Svalbard: indications from U–Pb geochronology and structural analysis. *J Geol Soc.* 2022;179(4): jgs2021–053. 10.1144/jgs2021-053

[ref-107] Korago EA Kovaleva GN Lopatin BG : The Precambrian rocks of Novaya Zemlya.In: *The Neoproterozoic Timanide Orogen of Eastern Baltica*. edited by: Gee, D. G. and Pease, V., Geological Society of. London, Memoirs,2004; 30:135–143. 10.1144/GSL.MEM.2004.030.01.12

[ref-108] Kostyuchenko A Sapozhnikov R Egorkin A : Crustal structure and tectonic model of northeastern Baltica, based on deep seismic and potential field data.In: *European Lithosphere Dynamics*. edited by: Gee, D. G. and Stephenson, R. A., Geological Society of London, Memoirs,2006;32(1):521–539. 10.1144/GSL.MEM.2006.032.01.32

[ref-109] Krauskopf KB : Igneous and Metamorphic Rocks of the Øksfjord Area, Vest-Finnmark. *Norg Geol Unders.* 1954;188:29–50. Reference Source

[ref-111] Krill AG Rodgers J Sundvoll B : Alternative to the Finnmarkian–Scandian interpretation on Magerøya, northern Norway. *Norsk Geol Tidsskr.* 1988;68:171–185.

[ref-110] Krill AG Zwaan KB : Reinterpretation of Finnmarkian deformation on western Sørøy, northern Norway. *Norsk Geol Tidsskr.* 1987;67:15–24. Reference Source

[ref-112] Kuznetsov NB Soboleva AA Udoratina OV : Pre-Ordovician tectonic evolution and volcano–plutonic associations of the Timanides and northern Pre-Uralides, northeast part of the East European Craton. *Gondwana Res.* 2007;12(3):305–323. 10.1016/j.gr.2006.10.021

[ref-113] Larionov AN Andreichev VA Gee DG : The Vendian alkaline igneous suite of northern Timan: ion microprobe U–Pb zircon ages of gabbros and syenite.In: *The Neoproterozoic Timanide Orogen of Eastern Baltica*. edited by: Gee, D. G. and Pease, V., Geological Society of London, Memoirs,2004;30:69–74. 10.1144/GSL.MEM.2004.030.01.07

[ref-114] Larsen RB Grant T Sørensen BE : Portrait of a giant deep-seated magmatic conduit system: the Seiland Igneous Province. *Lithos.* 2018;296-299:600–622. 10.1016/j.lithos.2017.11.013

[ref-115] Lea H : Analysis of Late plaeozoic-Mesozoic brittle faults and fractures in West-Finnmark: geometry, kinematics, fault rocks and the relationship to offshore structures on the Finnmark Platform in the SW Barents Sea.Master’s Thesis, University of Tromsø,2016;129. Reference Source

[ref-116] Leslie AG Nutman AP : Episodic tectono-thermal activity in the southern part of the East Greenland Caledonides. *Geology of Greenland Survey Bulletin.* 2000;186:42–49. 10.34194/ggub.v186.5214

[ref-117] Leslie AG Nutman AP : Evidence for Neoproterozoic orogenesis and early high temperature Scandian deformation events in the southern East Greenland Caledonides. *Geol Mag.* 2003;140(3):309–333. 10.1017/S0016756803007593

[ref-118] Li ZX Bogdanova SV Collins AS : Assembly, configuration, and break-up history of Rodinia: a synthesis. *Precambrian Res.* 2008;160(1–2):179–210. 10.1016/j.precamres.2007.04.021

[ref-229] Li QC Zhu GM : Singularity detection of the thin bed seismic sifgnals with wavelet transform. *Acta Seismologica Sinica.* 2000;13(1):61–66. 10.1007/s11589-000-0082-z

[ref-119] Lippard SJ Prestvik T : Carboniferous dolerite dykes on Magerøy: new age determination and tectonic significance. *Norsk Geol Tidsskr.* 1997;77:159–163. Reference Source

[ref-120] Lippard SJ Roberts D : Fault systems in Caledonian Finnmark and the southern Barents Sea. *Norg Geol Unders B.* 1987;410:55–64.

[ref-121] Lopatin BG Pavlov LG Orgo VV : Tectonic Structure of Novaya Zemlya. *Polarforschung.* 2001;69:131–135. Reference Source

[ref-123] Lorenz H Gee DG Larionov AN : The Grenville-Sveconorwegian orogen in the high Artic. *Geol Mag.* 2012;149(5):875–891. 10.1017/S0016756811001130

[ref-122] Lorenz H Pystin AM Olovyanishnikov VG : Neoproterozoic high-grade metamorphism of the Kanin Peninsula, Timanide Orogen, northern Russia.In: *The Neoproterozoic Timanide Orogen of Eastern Baltica*. edited by: Gee, D. G. and Pease, V., Geological Society of London, Memoirs,2004;30:59–68. 10.1144/GSL.MEM.2004.030.01.06

[ref-124] Majka J Mazur S Manecki M : Late Neoproterozoic amphibolite-facies metamorphism of a pre-Caledonian basement block in southwest Wedel Jarlsberg Land, Spitsbergen: new evidence from U-Th-Pb dating of monazite. *Geol Mag.* 2008;145(6):822–830. 10.1017/S001675680800530X

[ref-125] Manecki M Holm DK Czerny J : Thermochronological Evidence for Late Proterozoic (Vendian) Cooling in Southwest Wedel Jarlsberg Land, Spitsbergen. *Geol Mag.* 1998;135(1):63–9. 10.1017/S0016756897008297

[ref-126] Mazur S Czerny J Majka J : A strike-slip terrane boundary in Wedel Jarlsberg Land, Svalbard, and its bearing on correlations of SW Spitsbergen with the Pearya Terrane and Timanide Belt. *J Geol Soc.* London.2009;166(3):529–544. 10.1144/0016-76492008-106

[ref-127] McClelland WC von Gosen W Piepjohn K : Tonian and Silurian magmatism in Nordaustlandet: Svalbard’s place in the Caledonian orogen. *Geol S Am S.* 2018;541:63–79. 10.1130/2018.2541(04)

[ref-128] Meert JG Walderhaug HJ Torsvik TH : Age and paleomagnetic signature of the Alnø carbonatite complex (NE Sweden): additional controversy for the Neoproterozoic paleoposition of Baltica. *Precambrian Res.* 2007;154(3–4):159–174. 10.1016/j.precamres.2006.12.008

[ref-129] Michalski K Manby GM Nejbert K : Palaeomagnetic investigations across Hinlopenstretet border zone: from Caledonian metamorphosed rocks of Ny Friesland to foreland facies of Nordaustlandet (NE Svalbard). *J Geol Soc.* London.2022;180:1. 10.1144/jgs2021-167

[ref-130] Nasuti A Roberts D : Using geophysics to follow and model the Precambrian basement terranes beneath the Caledonian nappes in Finnmark, northern Norway: a case study. *Precambrian Res.* 2023;384: 106934. 10.1016/j.precamres.2022.106934

[ref-132] Nasuti A Roberts D Dumais MA : New high-resolution aeromagnetic and radiometric surveys in Finnmark and North Troms: linking anomaly patterns to bedrock geology and structure. *Nor J Geol.* 2015b;95(3–4):217–244. 10.17850/NJG95-3-10

[ref-131] Nasuti A Roberts D Gernigon L : Multiphase mafic dykes in the Caledonides of northern Finnmark revealed by a new high-resolution aeromagnetic dataset. *Nor J Geol.* 2015a;95(3–4):285–298. 10.17850/njg95-3-02

[ref-133] Nystuen JP : Senprekambrium, fra Urtid til Oldtid; 850– 542 millioner år.In: *Landet Blir Til: Norges Geologi.*edited by: Ramberg, I.B., Bryhni, I., Nøttvedt, A. and Rangnes. K., Trondheim, Norway, Norsk Geologisk Forening,2013;2013:120–147.

[ref-134] Ohta Y Larionov AN : Grenvillian single-grain zircon Pb age of a granitic rock from the southern island of Hesteskoholmen, Liefdefjorden, northwestern Spitsbergen, Svalbard. *Polar Res.* 1998;17(2):147–154. 10.3402/polar.v17i2.6615

[ref-135] Ohta Y Larionov AN Tebenkov AM : Single-grain zircon dating of the metamorphic and granitic rocks from the Biscayarhalvøya–Holtedahlfonna zone, north-west Spitsbergen. *Polar Res.* 2003;22(2):247–265. 10.3402/polar.v22i2.6459

[ref-136] Olovyanishnikov VG Roberts D Siedlecka A : Tectonics and Sedimentation of the Meso- to Neoproterozoic Timan-Varanger Belt along the Northeastern Margin of Baltica. *Polarforschung.* 2000;68:267–274. Reference Source

[ref-137] Osmundsen PT Andersen TB : Caledonian compressional and late-orogenic extensional deformation in the Staveneset area, Sunnfjord, Western Norway. *J Struct Geol.* 1994;16(10):1385–1401. 10.1016/0191-8141(94)90004-3

[ref-235] Ottesen D Stokes CR Rise L : Ice-sheet dynamics and ice streaming along the coastal parts of northern Norway. *Quat Sci Rev.* 2008;27(9–10):922–940. 10.1016/j.quascirev.2008.01.014

[ref-138] Pastore Z Fichler C McEnroe SA : The deep crustal structure of the mafic-ultramafic Seiland Igneous Province of Norway from 3-D gravity modelling and geological implications. *Geophys J Int.* 2016;207(3):1653–1666. 10.1093/gji/ggw362

[ref-141] Pease V Daly JS Elming SÅ : Baltica in the Cryogenian, 850–630 Ma. *Precambrian Res.* 2008;160(1):46–65. 10.1016/j.precamres.2007.04.015

[ref-140] Pease V Dovzhikova E Beliakova L : Late Neoproterozoic granitoid magmatism in the basement of the Pechora Basin, NW Russia: geochemical constraints indicate westward subduction beneath NE Baltica.In: *The Neoproterozoic Timanide Orogen of Eastern Baltica.*edited by: Gee, D. G. and Pease, V., *Geological Society of London Memoirs*.2004;30:75–85. 10.1144/GSL.MEM.2004.030.01.08

[ref-139] Pease V Gee DG Vernikovsky V : Geochronological evidence for late-Grenvillian magmatic and metamorphic events in central Taimyr, northern Siberia. *Terra Nova.* 2001;13(4):270–280. 10.1046/j.1365-3121.2001.00351.x

[ref-142] Péron-Pinvidic G Osmundsen PT : From orogeny to rifting: insights from the Norwegian 'reactivation phase'. *Nature Sci Rep.* 2020;10(1): 14860. 10.1038/s41598-020-71893-z 32908226 PMC7481783

[ref-143] Pettersson CH Pease V Frei D : U–Pb zircon provenance of metasedimentary basement of the Northwestern Terrane, Svalbard: implications for the Grenvillian–Sveconorwegian orogeny and development of Rodinia. * Precambrian Res.* 2009a;175(1–4):206–220. 10.1016/j.precamres.2009.09.010

[ref-144] Pettersson CH Tebenkov AM Larionov AN : Timing of migmatization and granite genesis in the Northwestern Terrane of Svalbard, Norway: implications for regional correlations in the Arctic Caledonides. *J Geol Soc.* London,2009b;166(1):147–158. 10.1144/0016-76492008-023

[ref-145] Peucat JJ Ohta Y Gee DG : U-Pb, Sr and Nd evidence for Grenvillian and latest Proterozoic tectonothermal activity in the Spitsbergen Caledonides, Arctic Ocean. *Lithos.* 1989;22(4):275–285. 10.1016/0024-4937(89)90030-3

[ref-146] Pharaoh TC Macintyre RM Ramsay DM : K-Ar age determinations on the Raipas suite in the Komagfjord Window, northern Norway. *NORSK GEOL TIDSSKR.* 1982;62:51–57. Reference Source

[ref-147] Pharaoh TC Ramsay DM Jansen Ø : Stratigraphy and Structure of the Northern Part of the Repparfjord-Komagfjord Window, Finnmark, Northern Norway. *NORG GEOL UNDERS.* 1983;377:1–45. Reference Source

[ref-148] Qin X Zhao B Li F : Deep structural research of the South China Sea: Progresses and directions. *China Geology.* 2019;2(4):530–540. 10.31035/cg2018125

[ref-149] Ramsay DM Sturt BA : The syn-metamorphic emplacement of the Magerøy Nappe. *NORSK GEOL TIDSSKR.* 1976;56(3):291–307. Reference Source

[ref-150] Ramsay DM Sturt BA Andersen TB : The sub-Caledonian Unconformity on Hjlmsøy – New Evidence of Primary Basement/Cover Relations in the Finnmarkian Nappe Sequence. *Norges geol Unders.* 1979;351:1–12. Reference Source

[ref-151] Ramsay DM Sturt BA Jansen Ø : The tectonostratigraphy of western Porsangerhalvøya Finnmark, north Norway. In: *The Caledonides Orogen - Scandinavia and Related Areas*. edited by: D. G. Gee and B. A. Sturt, John Wiley & Sons Ltd,1985;611–619. Reference Source

[ref-152] Ravna EJK Roux MRM : Metamorphic Evolution of the Tønsvika Eclogite, Tromsø Nappe—Evidence for a New UHPM Province in the Scandinavian Caledonides. *Int Geol Rev.* 2006;48(10):861–881. 10.2747/0020-6814.48.10.861

[ref-153] Reitan PH : The geology of the Komagfjord tectonic window of the Raipas suite, Finnmark, Norway. *NORG GEOL UNDERS.* 1963;221:71. Reference Source

[ref-154] Rekant P Sobolev N Portnov A : Basement segmentation and tectonic structure of the Lomonosov Ridge, arctic Ocean: insights from bedrock geochronology. *J Geodyn.* 2019;128:38–54. 10.1016/j.jog.2019.05.001

[ref-155] Remizov D : Metabasite Basement of the Voikar Island Arc in the Polar Urals. *Polarforschung.* 2006;73(2/3):49–57. Reference Source

[ref-156] Remizov D Pease V : The Dzela complex, Polar Urals, Russia: a Neoproterozoic island arc. In: *The Neoproterozoic Timanide Orogen of Eastern Baltica*. edited by: Gee, D. G. and Pease, V., *Geological Society of London Memoirs*.2004;30:107–123. 10.1144/GSL.MEM.2004.030.01.10

[ref-157] Rice AHN : Stratigraphic overlap of the late Proterozoic Vadsø and Barents Sea Groups and correlation across the Trollfjorden-Komagelva Fault, Finnmark, North Norway. *NORSK GEOL TIDSSKR.* 1994;74:48–57. Reference Source

[ref-158] Rice AHN : Restoration of the External Caledonides, Finnmark, North Norway. In: *New Perspectives on the Caledonides of Scandinavia and Related Areas*. edited by: Corfu, F., Gasser, D. and Chew, D. M., Geological Society, London, Special Publications,2014;390:271–299. 10.1144/sp390.18

[ref-159] Rice AHN Frank W : The early Caledonian (Finnmarkian) event reassessed in Finnmark: ^40^Ar/ ^39^Ar cleavage age data from NW Varangerhalvøya, N. Norway. *Tectonophysics.* 2003;374(3–4):219–236. 10.1016/S0040-1951(03)00240-3

[ref-161] Rice AHN Ntaflos T Gayer RA : Metadolerite geochronology and dolerite geochemistry from East Finnmark, northern Scandinavian Caledonides. *Geol Mag.* 2004;141(3):301–318. 10.1017/S001675680300788X

[ref-160] Rice AHN Reiz W : The structural relations and regional tectonic implications of metadolerite dykes in the Kongsfjord Formation, North Varanger Region, Finnmark, N. Norway. *NORSK GEOL TIDSSKR.* 1994;74:152–165. Reference Source

[ref-162] Roberts D : Tectonic Deformation in the Barents Sea Region of Varanger Peninsula, Finnmark. *NORG GEOL UNDERS.* 1972;282:1–39. Reference Source

[ref-163] Roberts D : Geologisk kart over Norge, berggrunnskart, Hammerfest 1: 250 000. *NORG GEOL UNDERS.* 1973.

[ref-164] Roberts D : Geochemistry of Dolerite and Metadolerite Dykes from Varanger Peninsula, Finnmark, North Norway. *NORG GEOL UNDERS.* 1975;322:55–72. Reference Source

[ref-165] Roberts D : Geologisk kart over Norge, berggrunnskart NORKAPP 1: 250 000. *NORG GEOL UNDERS.* 1981.

[ref-166] Roberts D : SVÆRHOLT, berggrunnsgeologisk kart 2136 1 -1: 50000, foreløpig utgave. *NORG GEOL UNDERS.* 1987.

[ref-167] Roberts D : Caledonian and Baikalian tectonic structures on Varanger Peninsula, Finnmark, Norway, and coastal areas of Kola Peninsula, NW Finnmark. *NORG GEOL UNDERS B.* 1996;431:59–65. Reference Source

[ref-168] Roberts D : Berggrunnskart HONNINGSVÅG - Geologisk kart over Norge, M 1:250 000. *NORG GEOL UNDERS.* 1998.

[ref-169] Roberts D : The Scandinavian Caledonides: event chronology, palaeogeographic settings and likely modern analogues. *Tectonophysics.* 2003;365(1–4):283–299. 10.1016/S0040-1951(03)00026-X

[ref-170] Roberts D : Palaeocurrent data from the Kalak Nappe Complex northern Norway: a key element in models of terrane affiliation. *Nor J Geol.* 2007;87(3):319–328. Reference Source

[ref-171] Roberts D : Berggrunnskart SKJØTNINGBERG 2237 III, M 1:50000. *NORG GEOL UNDERS.* 2008a.

[ref-172] Roberts D : Berggrunnskart MEHAMN 2237 II, M 1:50000. *NORG GEOL UNDERS.* 2008b.

[ref-173] Roberts D : Age of the Hamningberg dolerite dyke, Varanger Peninsula, Finnmark: Devonian rather than Vendian - a revised interpretation. *NORG GEOL UNDERS B.* 2011;451:32–36. Reference Source

[ref-181] Roberts D Chand S Rise L : A half-graben of inferred Late Palaeozoic age in outer Varangerfjorden, Finnmark: evidence from seismic reflection profiles and multibeam bathymetry. *Nor J Geol.* 2011;91:191–200. Reference Source

[ref-182] Roberts RJ Corfu F Torsvik TH : Short-lived mafic magmatism at 560–570 Ma in the northern Norwegian Caledonides: U–Pb zircon ages from the Seiland Igneous Province. *Geol Mag.* 2006;143(6):887–903. 10.1017/S0016756806002512

[ref-174] Roberts D Lippard SJ : Inferred Mesozoic faulting in Finnmark: current status and offshore links. *NORG GEOL UNDERS.* 2005;443:55–60. Reference Source

[ref-180] Roberts D Mitchell JG Andersen TB : A post-Caledonian dolerite dyke from Magerøy North Norway: age and geochemistry. *Nor J Geol.* 1991;71:289–294. Reference Source

[ref-175] Roberts D Onstott TC : ^40^Ar/ ^39^Ar laser microprobe analyses and geochemistry of dolerite dykes from the Rybachi and Sredni Peninsulas, NW Kola, Russia. *NORG GEOL UNDERS.* Special Publication,1995;7:307–314.

[ref-176] Roberts D Siedlecka A : Provenance and sediment routing of Neoproterozoic formations on the Varanger, Nordkinn, Rybachi and Sredni peninsulas, North Norway and Northwest Russia: a review. *NORG GEOL UNDERS B.* 2012;452:1–19. Reference Source

[ref-177] Roberts D Siedlecka A : Berggrunnskart HOPSEIDET 2236-1, M 1:50 000, Foreløpig utgave. *NORG GEOL UNDERS.* 2013. Reference Source

[ref-178] Roberts D Siedlecka A : Revised stratigraphy and correlation of the Neoproterozoic successions of Varanger Peninsula, East Finnmark, northern Norway, and the Rybachi-Sredni peninsulas and Kildin Island in Northwest Russia. *NORG GEOL UNDERS B.* 2022;457:1–21. Reference Source

[ref-179] Roberts D Williams GD : Berggrunnskart Kjøllefjord 2236 IV, M 1: 50.000, foreløpig utgave. *NORG GEOL UNDERS.* 2013. Reference Source

[ref-183] Roberts RJ Corfu F Torsvik TH : Age of Alkaline rocks in the Seiland Igneous Province, Northern Norway. *J Geol Soc.* London,2010;167:71–81. 10.1144/0016-76492009-014

[ref-184] Robins B : Nordkapp berggrunnskart 2037 2 - 1: 50000, foreløpig utgave. *Norges geologiske undersøkelse.* 1990a.

[ref-185] Robins B : Skarsvåg berggrunnsgeologisk kart 2137 3 - 1: 50000, foreløpig utgave. *Norges geologiske undersøkelse.* 1990b.

[ref-186] Robins B Gardner PM : The magmatic evolution of the Seiland province, and Caledonian plate boundaries in northern Norway. *Earth Planet Sci Lett.* 1975;26(2):167–178. 10.1016/0012-821X(75)90084-9

[ref-187] Rosa D Majka J Thrane K : Evidence for Timanian-age basement rocks in North Greenland as documented through U-Pb zircon dating of igneous xenoliths from the Midtkap volcanic centers. *Precambrian Res.* 2016;275:394–405. 10.1016/j.precamres.2016.01.005

[ref-188] Rykkelid E : E69 “FATIMA” - Geologisk undersøkelse av tunnel under Magerøysundet. *Veglaboratoriet, Statens Vegvesen*.1992.

[ref-189] Siedlecka A : Late Precambrian Stratigraphy and Structure of the North-Eastern Margin of the Fennoscandian Shield (East Finnmark - Timan Region). *Norges geologiske undersøkelse.* 1975;316:313–348. Reference Source

[ref-196] Siedlecka A Reading HG Williams GD : Berggrunnskart LANGFJORDEN 2236 II, M 1: 50 000, foreløpig utgave. *Norges geologiske undersøkelse.* 2006.

[ref-191] Siedlecka A Roberts D : The bedrock geology of Varanger Peninsula, Finnmark, North Norway: an excursion guide.Norges geologiske undersøkelse Special Publication.1992;5:1–45. Reference Source

[ref-192] Siedlecka A Roberts D : Report from a visit to the Komi Branch of the Russian Academy of Sciences in Syktyvkar, Russia, and from fieldwork in the central Timans, August 1995. *Norges Geologiske Undersøkelse Report.* 1995;95(149):32. Reference Source

[ref-193] Siedlecka A Roberts D : Geology of the Båtsfjordfjellet drillcore, Varanger Peninsula, Finnmark, northern Norway. *Norges Geologiske Undersøkelse Bulletin.* 2012;452:20–29. Reference Source

[ref-194] Siedlecka A Siedlecki S : Some new aspects of the geology of Varanger peninsula (Northern Norway). *Norges Geologiske Undersøkelse.* 1967;247:288–306. Reference Source

[ref-195] Siedlecka A Siedlecki S : Late Precambrian sedimentary rocks of the Tanafjord-Varangerfjord region of Varanger Peninsula, Northern Norway. In: Roberts D, Gustavson M, eds: *The Caledonian Geology of Northern Norway*. Norges geologiske undersøkelse,1971; **269**:246–294. Reference Source

[ref-197] Siedlecki S : Geologisk kart over Norge, berggrunnskart Vadsø - M 1: 250 000. *Norges Geologiske Undersøkelse.* 1980.

[ref-198] Siedlecki S Levell BK : Lithostratigraphy of the Late Precambrian Løkvikfjell Group on Varanger Peninsula, East Finnmark, North Norway. *Norges Geologiske Undersøkelse.* 1978;343:73–85. Reference Source

[ref-199] Sirotkin AN Evdokimov AN : Vendian age of igneous rocks of the Chamberlain valley area (Northern part of the Wedel Jarlsberg Land, Svalbard Archipelago). *J Min Inst.* 2022;255:419–434. 10.31897/PMI.2022.20

[ref-200] Slagstad T Melezhik VA Kirkland CL : Carbonate isotope chemostratigraphy suggests revisions to the geological history of the West Finnmark Caledonides, northern Norway. *J Geol Soc.* London,2006;163:277–289. 10.1144/0016-764905-021

[ref-201] Slagstad T Roberts NMW Marker M : A non-collisional, accretionary Sveconorwegian orogen. *Terra Nova.* 2013;25(1):30–37. 10.1111/ter.12001

[ref-231] Slagstad T Saalmann K Kirkland CL : Late Neoproterozoic-Silurian tectonic evolution of the Rödingsfjället Nappe Complex, orogen-scale correlations and implications for the Scandian suture.In: *Pannotia to Pangaea: Neoproterozoic and Paleozoic Orogenic Cycles in the Circum-Atlantic Region.*Geological Society, London, Special Publications,2020;503:279–304. 10.1144/SP503-2020-10

[ref-202] Soper NJ Harris AL : Proterozoic orogeny questioned: a view from Scottish Highland field workshops, 1995–1996. *SCOT J GEOL.* 1997;33(2):187–190.

[ref-203] Spencer CJ Cawood PA Hawkesworth CJ : Generation and preservation of continental crust in the Grenville Orogeny. *Geosci Front.* 2015;6(3):357–372. 10.1016/j.gsf.2014.12.001

[ref-204] Speedyman DI : The Husfjord Plutonic complex, Sørøy, Northern Norway. *NGU.* 1983;378:1–48. Reference Source

[ref-205] Stumpfl EF Sturt BA : A preliminary account of the geochemistry and ore parageneses of some Caledonian basic igneous rocks from Sørøy, Northern Norway. *NGU.* 1964;234:196–230. Reference Source

[ref-207] Sturt BA Pringle IR Ramsay DM : The Finnmarkian phase of the Caledonian Orogeny. *J Geol Soc.* London,1978;135:597–610. 10.1144/gsjgs.135.6.0597

[ref-206] Sturt BA Ramsay DM : The alkaline complex of the Breivikbotn area, Sørøy, Northern Norway. *NGU.* 1965;231:165. Reference Source

[ref-208] Sun W : Initiation and evolution of the South China Sea: an overview. *Acta Geochim.* 2016;35(3):215–225. 10.1007/s11631-016-0110-x

[ref-209] Tegner C Andersen TB Kjøll HJ : A mantle plume origin for the Scandinavian Dyke complex: a “Piercing Point” for 615 Ma Plate reconstruction of Baltica. *Geochem Geophys Geosystems.* 2019;20(2):1075–1094. 10.1029/2018GC007941

[ref-232] TGS: Seismic processing report, FP13 Finnmark platform, May 2013 – October 2014, Project Number 724.2014.

[ref-233] TGS-NOPEC: Seismic data processing Barents Sea South. *Processing Project.* 2001;273:21.

[ref-211] Torgersen E Viola G Sandstad JS : Revised structure and stratigraphy of the Northwestern Repparfjord Tectonic Window, Northern Norway. *Nor J Geol.* 2015;95(3, 4):397–422. 10.17850/njg95-3-06

[ref-210] Torgersen E Viola G Zwingmann H : Structural and temporal evolution of a reactivated brittle-ductile fault - part II: timing of fault initiation and reactivation by K-Ar dating of synkinematic illite/muscovite. *Earth Planet Sci Lett.* 2014;407:221–233. 10.1016/j.epsl.2014.09.051

[ref-212] Torsvik TH Rehnström EF : Cambrian palaeomagnetic data from Baltica: implications for true polar wander and Cambrian palaeogeography. *J Geol Soc.* London,2001;158:321–329. 10.1144/jgs.158.2.321

[ref-214] Torsvik TH Smethurst MA Burke K : Large igneous provinces generated from the margins of the large low-velocity provinces in the deep mantle. *Geophys J Int.* 2006;167(3):1447–1460. 10.1111/j.1365-246X.2006.03158.x

[ref-213] Torsvik TH Trench A : The Ordovician history of the Iapetus Ocean in Britain: new palaeomagnetic constraints. *J Geol Soc.* London,1991;148(3):423–425. 10.1144/gsjgs.148.3.0423

[ref-215] Townsend C : Thrust transport directions and thrust sheet restoration in the Caledonides of Finnmark, North Norway. *J Struct Geol.* 1987;9(3):345–352. 10.1016/0191-8141(87)90057-5

[ref-216] Townsend C Roberts D Rice AHN : The Gaissa Nappe, Finnmark, North Norway: an example of a deeply eroded external imbricate zone within the Scandinavian Caledonides. *J Struct Geol.* 1986;8(3–4):431–440. 10.1016/0191-8141(86)90061-1

[ref-217] Trettin HP : Pearya: a composite terrane with Caledonian affinities in northern Ellesmere Island. *Can J Earth Sci.* 1987;24(2):224–245. 10.1139/e87-025

[ref-218] Trettin HP Parrish R Loveridge WD : U-Pb age determinations on proterozoic to Devonian rocks from Northern Ellesmere Island, Arctic Canada. *Can J Earth Sci.* 1987;24(2):246–256. 10.1139/e87-026

[ref-234] Vorren TO Kristoffersen Y Andreassen K : Geology of the inner shelf west of North Cape, Norway. *Norsk Geologisk Tidsskrift.* 1986;66:99–105. Reference Source

[ref-219] Vasey DA Cowgill E Cooper KM : A preliminary framework for magmatism in modern continental back-arc basins and its application to the Triassic-Jurassic tectonic evolution of the Caucasus. *Geochem Geophys Geosystems.* 2021;22(6): e2020GC009490. 10.1029/2020GC009490

[ref-221] Viola G Mattila J Zwingmann HM : Structural and K/Ar Illite Geochronoligcal Constraints on the Brittle Deformation History of the Olkiluoto Region. Southwest Finland,2011;174. Reference Source

[ref-220] Waltham T : Norway: the best of boudins. *Geology Today.* 2003;18(4):130–131.

[ref-222] Ziemniak G Kosminska K Petrik I : Th-U-total Pb monazite geochronology records Ordovician (444 Ma) metamorphism/partial melting and Silurian (419 Ma) thrusting in the Kåfjord Nappe, Norwegian Arctic Caledonides. *Geol Carpath.* 2019;70(6):494–511. 10.2478/geoca-2019-0029

[ref-223] Zwaan KB : Geology of the West Troms Basement complex, Northern Norway, with emphasis on the Senja Shear Belt: a preliminary account. *NGU Bulletin.* 1995;427:33–36. Reference Source

