# Supplementary material for: Caledonian reactivation and reworking of Timanian thrust systems and implications for latest Mesoproterozoic to mid-Paleozoic tectonics and magmatism in northern Baltica
Source: Open Res Eur. 2024 Oct 15;4:30. Originally published 2024 Feb 16. [Version 2] doi: 10.12688/openreseurope.17033.2 (PMC11751614; doi:10.12688/openreseurope.17033.2)
Supplement: Peer review history

# Reviewer report: Reviewer response for version 2

**DOI:** 10.21956/openreseurope.20071.r48581

**Published:** 2025-01-21

**Authors:** Ed Landing

**Affiliations:** 1 New York State Museum, Albany, New York, USA

**Overall summary: **After a thorough read through the Koehl and Stokmo paper I see that Dr. Martins’ review of the original manuscript was quite thorough, but, unfortunately, the revision did not “improve” the paper by implementing Dr. Martins’ suggestions. The article has been on line for almost a year now, and perhaps Koehl and Stokmo should be seen as having done a useful review that (this reader) sees as a useful trove of information. Let the existing revision be accepted.

 Koehl’s publications show that he is truly a master of this very thoroughly studied northern Norwegian region. But what is new seems relatively minor, with a new understanding of Caledonian modification of the KTFZ…..

 The seismic and magnetic study of the field area is a very interesting approach (and the figures are useful). But, as Dr. Martins noted and using a better adjective—the text is “exhausting” with the discussions chopped up and buried in line after line of cited references in sentences that sometimes go on for 15+ lines.

 The foreign reader is also boggled by the use of a huge number of localities (the major Varanger Peninsula; Nord Kinn, Svaerholt, Porsanger, Sredni, Rybuchi peninsulas; Svalbald is always cited but only Spitzbergen is on Fig. 1) and some lithologic units (Berlevag Fm.) that are not in any figure. If a place is cited, please put it on a figure. Perhaps Fig. 1a should be full page size. A small improvement of Fig. 1 would be surrounding the land in Fig. 1b with blue so that can be easily compared with Fig. 1a.

 The major “Conclusion” on page 2 of the geometry of the KTFZ somehow does not lead to the four “implications.” That of a narrow Iapetus is interesting, but this reader can’t find the discussion of this.

Is the study design appropriate and does the work have academic merit?

Partly

Is the work clearly and accurately presented and does it cite the current literature?

Yes

If applicable, is the statistical analysis and its interpretation appropriate?

Not applicable

Are all the source data underlying the results available to ensure full reproducibility?

Yes

Are the conclusions drawn adequately supported by the results?

Partly

Are sufficient details of methods and analysis provided to allow replication by others?

Yes

Reviewer Expertise:

Stratigraphy, biostratigraphy, and paleogeography of terminal Ediacaran-early Paleozoic

I confirm that I have read this submission and believe that I have an appropriate level of expertise to confirm that it is of an acceptable scientific standard, however I have significant reservations, as outlined above.

# Reviewer report: Reviewer response for version 2

**DOI:** 10.21956/openreseurope.20071.r45168

**Published:** 2025-01-16

**Authors:** Wenjiao Xiao

**Affiliations:** 1 University of Chinese Academy of Sciences, Beijing, China

Thanks. I have carefully read the revised manuscript and I am satisfied with the revision. I would like to recommend its acceptance for indexing.

Is the study design appropriate and does the work have academic merit?

Partly

Is the work clearly and accurately presented and does it cite the current literature?

Partly

If applicable, is the statistical analysis and its interpretation appropriate?

Partly

Are all the source data underlying the results available to ensure full reproducibility?

Partly

Are the conclusions drawn adequately supported by the results?

Partly

Are sufficient details of methods and analysis provided to allow replication by others?

Partly

Reviewer Expertise:

Geodynamics, tectonics

I confirm that I have read this submission and believe that I have an appropriate level of expertise to confirm that it is of an acceptable scientific standard.

# Reviewer report: Reviewer response for version 2

**DOI:** 10.21956/openreseurope.20071.r48931

**Published:** 2025-01-09

**Authors:** Gustavo Martins

**Affiliations:** 1 TGS, Singapore, Singapore

This interesting manuscript adds needed structural information that can be useful to future research such as Barents basement rocks, its challenging consolidation mechanisms, and similar. As stated by the authors, their work presents “a new, more realistic interpretation of the Trollfjorden–Komagelva Fault Zone off northwestern Norway.” The authors also state that the “aim is to explore the Finnmark Platform for traces of similarly folded Timanian structures and track their potential continuation onshore and in nearshore fjords.” They conclude that the Trollfjorden–Komagelva Fault Zone may continue offshore as a NE–SW-striking folded structure. Implications include from the development of the Finnmark structural area to challenging the traditional interpretation of a very extensive Iapetus Ocean.

 In my view, this paper could be indexed after a revision that includes shortening and simplification. I will try to expose my main observations below as succinct as possible.

**Text organization and figures**

- Although informative, the text is rather exhaustive. I do not say this in a negative sense, as the text clearly shows the author’s familiarity with the area. However, a reader who is unfamiliar with this area will likely have a hard time reading and absorbing this paper. Even though the Barents shelf and adjacent areas have much structural complexity and many officially named structures, I feel that there is an overuse of nomenclature. Such overuse may make it very difficult for readers who are unfamiliar with the area to follow the narrative. Because I believe the authors have interest in having their paper being exposed and used beyond the “local” audience, I strongly recommend that they “tighten up” the text and attempt reducing the amount of nomenclature. 
- If the authors are decisive in maintaining this excessive use of nomenclature, then at least a table listing the main structures and their location may be beneficial in helping the readers to better navigate the figures and text.
- One example of such overuse is Figure 1. Are structural elements such as Bjørnøya Basin, Sørvestsnaget Basin, Bjarmeland Platform, etc, necessary? Conversely, why are no structural elements named in the Russian side of the Barents shelf in Fig. 1 (e.g., Kola Monocline area)? In Fig. 1., most of "proven" Timanian thrusts, as well as the continuation of the target fault, are in the Russian side. If you include only the nomenclature needed in the paper, it will make Fig. 1, its caption, and the text easier to follow and/or to refer back to.
- Please indicate where your tectonostratigraphic columns are located in Figure 1 or include a mini map in Figure 2. You may also include orogenic timing in your columns.
- I am not sure if it is the journal’s style, but I find it odd that seismic profiles are in the Methods section. Why not move them to Results?
- In the seismic profiles, a mini map showing line location is beneficial.
- In the first paragraph of the introduction, is Fig. 4C-D supposed to be of Svalbard or of something else? Please re-write the sentence.
- Apparently, the location of the seismic profiles is only illustrated in Figure 7. Why not in Fig. 1 or Fig. 3, for example?
- In the tilt derivative maps, some white lines representing, if I am not mistaken, folded Caledonian dykes, are difficult to visualize in the uninterpreted figures. It is beneficial if you shortly clarify. For example, are there resolution issues? Processing? Are the structures naturally too subtle? Clarifying these will help the reader to better understand your data analysis and interpretation.
- You should avoid making interpretations in your results chapter. For example, on page 24, you ambitiously interpret “syn-orogenic foreland/piggy-back deposits ahead of the major thrust.” Please move this and similar interpretations to the discussion.
- Similarly, main seismic features such as rugose reflections, U-shaped reflections, and others, could be compiled and introduced in a “seismic facies” table.

**Introduction**

- You state that the Timanian orogeny is Neoproterozoic. However, in your block diagram (and elsewhere), you include Cambrian ages as well. Please correct the Timanian age as Neoproterozoic–Cambrian in the introduction, insert a reference, and correct elsewhere.
- Clarify what do you mean by Timanian “footprints.” Geochronology? Structures?  

**Geological Setting**

- The Grenvillian orogeny is key to your study, but you did not introduce this orogeny well. For example, you say that the Porsanger orogeny is a contractional event in northern Norway. Similarly, there could be at least 2-3 sentences providing a better general introduction of the Grenvillian orogeny.
- For the Timanian orogeny, please include Cambrian ages.
- The geological setting could use a sub-header addressing Timanian structures separately. Moreover, a paleogeographic map showing the Timanian orogen and its main structures could significantly help the reader. Do you happen to have any photos showing Timanian structures in outcrops? If you do, and considering your goal of possible continuation onshore, it would be great to see some of these Timanian structures in rocks.
- You should avoid wording such as “rifting of Iapetus” as this can cause confusion. Instead, it is better to use wording such as “continental rifting followed by opening of the Iapetus Ocean,” or similar.
- The authors should consider describing the Timanian orogeny similarly to their Caledonian introduction. Timanian structures should be in a sub-header instead.
- There is not much mention of post-Timanian extension as done for the Caledonides. For example, did the Timanian orogen collapsed? If Timanian post-extension is unknown, simply insert that more research is needed.

**Methods**

- The authors should consider moving data figures to the Results section
- Please do not list software such as QGIS or CorelDraw.

**Results**

-  This section is massive! The authors should try to simplify or at least reduce use of structural nomenclature, clarify or better introduce observed seismic features (e.g., a table), and better mention these in their figure captions. Terminology such as Upper Regional Unconformity should be introduced or removed. Similarly, interpretations should be moved to the Discussion section.

**Discussion**

- This is another massive section, and it is rather challenging to follow the author’s line of thought and relate it to their goals.
- The hypothesis of a narrower Iapetus Ocean seems ambitious. If the authors decide to put this hypothesis as a main implication/goal, then it would be beneficial to include a paleogeographic figure illustrating the traditional Iapetus interpretation and their interpretation side by side. The visualization of the author’s hypothesis becomes “lost” in the wall of text presented in the manuscript as it currently is.
- I found the discussion heavily based in seismic and I wonder if more “concrete” datasets such as wells or hopefully outcrop material could be included. As useful as seismic is, it is not an absolute dataset.
- Although very informative, the discussion reads complex. Use of subheadings and simplification of the writing and nomenclature usage should enhance the section significantly.

**Conclusions**

- It may be beneficial for the authors to rephrase some of their conclusions and mention to what extent their interpretations may have included speculation. Some of the conclusions give the impression that the authors are uncertain. For example, the use of “suggests” in #5. Hence, it is beneficial for the authors to indicate whether and which areas may need further research (e.g., width of the Iapetus Ocean).

**Limitations**

- Is there room for limitations in your study and analysis? Given that your goals are challenging, may lack data, and at times  contrast with traditional interpretations, it is beneficial to insert a short section or at least a few sentences in the discussion clarifying possible pitfalls, open questions, what complementary datasets future research should/could focus on that this study did not manage to include, and/or similar considerations.

I hope that the above feedback is helpful to the authors.

 Best regards,

 Gustavo Martins

Is the study design appropriate and does the work have academic merit?

Yes

Is the work clearly and accurately presented and does it cite the current literature?

Partly

If applicable, is the statistical analysis and its interpretation appropriate?

Not applicable

Are all the source data underlying the results available to ensure full reproducibility?

No

Are the conclusions drawn adequately supported by the results?

Partly

Are sufficient details of methods and analysis provided to allow replication by others?

Partly

Reviewer Expertise:

Tectonostratigraphy

I confirm that I have read this submission and believe that I have an appropriate level of expertise to confirm that it is of an acceptable scientific standard, however I have significant reservations, as outlined above.

# Reviewer report: Reviewer response for version 1

**DOI:** 10.21956/openreseurope.18407.r40249

**Published:** 2024-07-09

**Authors:** Wenjiao Xiao

**Affiliations:** 1 University of Chinese Academy of Sciences, Beijing, China

The results of this manuscript challenge the existing models regarding the extension of the Trollfjorden–Komagelva Fault Zone, offering a clearer and more definitive understanding of its continuation through reinterpreting previous geophysical results including seismic reflection, bathymetric, topographic, and magnetic data analysis. I think the novelty of the manuscript is stated very obviously. However, it needs a major revision. There are some reasons as outlined below:

 Major concerns:

 1. The authors stated that the seismic data were interpreted using Petrel. Did the authors use seismic data sourced from relevant database and utilize open-source software for data processing, or did they merely reinterpret seismic reflection profiles that were originally derived from previous studies conducted by other researchers? Please clarify this in the main text. If the geophysical findings presented in this manuscript originate from previous research publications by other scholars, please give a reference to relevant literature.

 2. I am uncertain if the reflection profiles presented in Figures 2 to 4 can be consistently reproduced through repetition of the same methodology or experiments.

 3. Another main problem of the manuscript is the introduction of the results and data is in a mess, such as some of the locations cannot be found in the figure, and the introduction of the results is not presented in temporal and spatial order to describe, making the paper difficult to read.

 Minor comments:

 1) The abstract should be presented as a paragraph and should introduce the background, data and results of the manuscript and the conclusion. However, the present manuscript does not meet this standard.

 2) The conclusions in the “abstract” are not based on the data of this study. Therefore, it is not acceptable.

 3) There are too many keywords for a published paper. In general, a published paper has less than 5-6 keywords.

 4) The fourth and fifth paragraphs of the “Introduction” should be presented in the Discussion section.

 5) The legends in the Figure 1 are too small to read.

 6) The structural legend for possible post-Caledonian dykes/sills is not shown in Figure 2.

 7) The background of the regional geology is too much. Shorten it.

Is the study design appropriate and does the work have academic merit?

Partly

Is the work clearly and accurately presented and does it cite the current literature?

Partly

If applicable, is the statistical analysis and its interpretation appropriate?

Partly

Are all the source data underlying the results available to ensure full reproducibility?

Partly

Are the conclusions drawn adequately supported by the results?

Partly

Are sufficient details of methods and analysis provided to allow replication by others?

Partly

Reviewer Expertise:

Geodynamics, tectonics

I confirm that I have read this submission and believe that I have an appropriate level of expertise to confirm that it is of an acceptable scientific standard, however I have significant reservations, as outlined above.

# Response

**Published:** 2024-09-13

**Authors:** Jean-Baptiste Koehl

**Affiliations:** Geosciences, Universitetet i Oslo, Oslo, Oslo, Norway

Dear Prof. Xiao, thank you very much for your input on the manuscript, it is highly appreciated. Here is our reply to your comments. We hope the changes we implemented improve the shortcomings of the manuscript highlighted by your comments and suggestions. Please do not hesitate to contact us shall this not be the case for some comments.  

**Comments by the reviewer**

Comment 1: 1. The authors stated that the seismic data were interpreted using Petrel. Did the authors use seismic data sourced from relevant database and utilize open-source software for data processing, or did they merely reinterpret seismic reflection profiles that were originally derived from previous studies conducted by other researchers? Please clarify this in the main text. If the geophysical findings presented in this manuscript originate from previous research publications by other scholars, please give a reference to relevant literature.

Response: As stated in the method chapter, the authors of the present manuscript interpreted existing (private) seismic reflection data from TGS and public data from the Norwegian Defense Research Establishment and the DISKOS database. Changes: None required. Comment 2: 2. I am uncertain if the reflection profiles presented in Figures 2 to 4 can be consistently reproduced through repetition of the same methodology or experiments. Response: Agreed. See response to comment 22 by Dr. Simon Stewart. Changes: See response to comment 22 by Dr. Simon Stewart.  

Comment 3: 3. Another main problem of the manuscript is the introduction of the results and data is in a mess, such as some of the locations cannot be found in the figure, and the introduction of the results is not presented in temporal and spatial order to describe Response: Agreed. Changes: Added location of Bjørnøya (Bj), Båsnæringsfjellet (Bn), Berlevåg (Bv), Ragnarokk Anticline (RA), Reinøykalven (Rk), Rolvsøya (Rv), Stikonjargga (Sk), and Tanafjorden (Tn) to Figure 1b.  

Comment 4: 1) The abstract should be presented as a paragraph and should introduce the background, data and results of the manuscript and the conclusion. However, the present manuscript does not meet this standard.

Response: Agreed. The present format of the abstract follows the standard of the journal. Changes: None.  

Comment 5: 2) The conclusions in the “abstract” are not based on the data of this study. Therefore, it is not acceptable.

Response: Partly agreed. However, the anchoring of the Seiland Igneous Province and of the metasedimentary rocks of the Kalak Nappe Complex, which they intrude, is indeed a conclusion from the present study, and so is the probable overestimation of the width of the Iapetus Ocean.

 \ Changes: Deleted “The NNE-dipping Trollfjorden–Komagelva Fault Zone merges with a recently identified northwest-dipping brittle–ductile thrust, the Sørøya–Ingøya shear zone, which was previously thought to have formed during the Caledonian Orogeny.” from the “result” section in the abstract because redundant with the following sentence. Also replaced “present study suggests” by “Trollfjorden–Komagelva Fault Zone may continue offshore as a NE–SW-striking folded structure. This has the following implications:”, and added “possibly” and “likely” in the following phrases.  

Comment 6: 3) There are too many keywords for a published paper. In general, a published paper has less than 5-6 keywords.

Response: Open Research Europe seems to allow high numbers of keywords. Thus, unless the journal editorial team recommends reducing the number of keywords, the authors of the present manuscript would prefer to keep them all. Changes: None. Awaiting feedback from the editorial team.  

Comment 7: 4) The fourth and fifth paragraphs of the “Introduction” should be presented in the Discussion section.

Response: Partly agreed regarding the fourth paragraph. However, the fifth paragraph must remain in the introduction since it presents some of the broad implications of the study, which is relevant information to add to the introduction of a scientific article. Having said that, it is only logical that the fourth paragraph, which is a direct implication of the results of the present study, also is mentioned in the introduction. However, the authors of the present manuscript concede that the models mentioned in paragraph four of the introduction should be better discussed and set in contrast to the present results in the discussion.

Changes: Added “bends into a WNW–ESE strike in the northeast and” and “rather than the commonly proposed WNW–ESE-striking segment of the Troms–Finnmark Fault Complex (Gabrielsen, 1984; Gabrielsen & Færseth, 1989; Lea, 2016; Roberts *et al.*, 2011” in the first paragraph of the “The western continuation of the TKFZ offshore” section of the discussion. Added “in the field”, “and on bathymetric data (Figure 6)”, and “In this case, truncation of WNW–ESE-striking Timanian fabrics by a top-southeast Caledonian shear zone (e.g., Koehl et al., 2018a) is not required.” in the second paragraph. Also added a new paragraph thereafter about the model by Koehl et al. (2019).  

Comment 8: 5) The legends in the Figure 1 are too small to read.

Response: Agreed.

Changes: The authors have asked the journal to enlarge Figure 1a and 1b as much as possible.  

Comment 9: 6) The structural legend for possible post-Caledonian dykes/sills is not shown in Figure 2.

Response: Disagreed.

Changes: The legend for post-Caledonian dykes/sills is included in Figure 2d.  

Comment 10: 7) The background of the regional geology is too much. Shorten it.

Response: Disagreed.

Changes: The authors of the present manuscript concede that the geological setting section is relatively long. However, it is necessary to acknowledge relevant previous works in the study area and around to set the stage for the issues approached in the discussion.

# Reviewer report: Reviewer response for version 1

**DOI:** 10.21956/openreseurope.18407.r38897

**Published:** 2024-04-28

**Authors:** Simon Stewart

**Affiliations:** 1 Saudi Aramco, Dhahran, Saudi Arabia

Please see the review in the separate PDF document linked here.

Is the study design appropriate and does the work have academic merit?

Yes

Is the work clearly and accurately presented and does it cite the current literature?

Partly

If applicable, is the statistical analysis and its interpretation appropriate?

Not applicable

Are all the source data underlying the results available to ensure full reproducibility?

No

Are the conclusions drawn adequately supported by the results?

Partly

Are sufficient details of methods and analysis provided to allow replication by others?

No

Reviewer Expertise:

Structural geology, reflection seismic interpretation.

I confirm that I have read this submission and believe that I have an appropriate level of expertise to state that I do not consider it to be of an acceptable scientific standard, for reasons outlined above.
